# Electrochemical studies of tris(cyclopentadienyl)thorium and uranium complexes in the +2, +3, and +4 oxidation states†

**DOI:** 10.1039/d1sc01906f

**Published:** 2021-05-07

**Authors:** Justin C. Wedal, Jeffrey M. Barlow, Joseph W. Ziller, Jenny Y. Yang, William J. Evans

**Affiliations:** Department of Chemistry, University of California Irvine California 92697 USA j.yang@uci.edu wevans@uci.edu

## Abstract

Electrochemical measurements on tris(cyclopentadienyl)thorium and uranium compounds in the +2, +3, and +4 oxidation states are reported with C_5_H_3_(SiMe_3_)_2_, C_5_H_4_SiMe_3_, and C_5_Me_4_H ligands. The reduction potentials for both U and Th complexes trend with the electron donating abilities of the cyclopentadienyl ligand. Thorium complexes have more negative An(iii)/An(ii) reduction potentials than the uranium analogs. Electrochemical measurements of isolated Th(ii) complexes indicated that the Th(iii)/Th(ii) couple was surprisingly similar to the Th(iv)/Th(iii) couple in Cp′′-ligated complexes. This suggested that Th(ii) complexes could be prepared from Th(iv) precursors and this was demonstrated synthetically by isolation of 
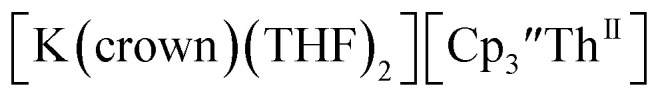
 directly from 
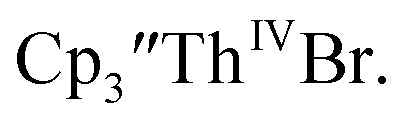
 UV-visible spectroelectrochemical measurements and reactions of 
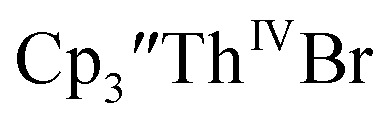
 with elemental barium indicated that the thorium system undergoes sequential one electron transformations.

## Abstract 2

Electrochemical determination of the reduction potentials for a variety of tris(cyclopentadienyl)uranium and thorium complexes, including data on U(ii) and Th(ii) complexes.

## Graphical abstract

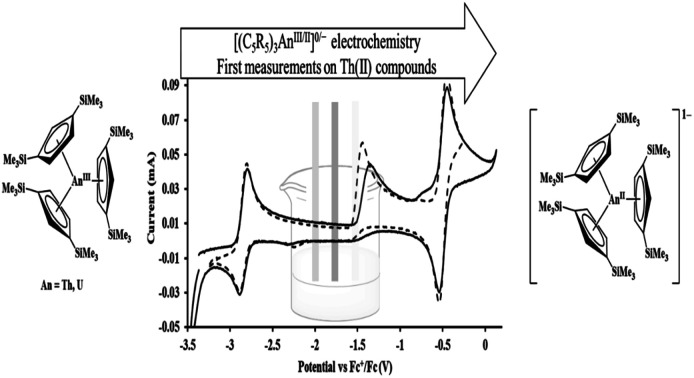

## Introduction

The redox chemistry of the actinide elements has recently undergone a significant change: the range of oxidation states available in crystallographically-characterizable molecular complexes has been extended to +2. The discovery of the first molecular example of U(ii) involved potassium graphite reduction of the tris(cyclopentadienyl) complex 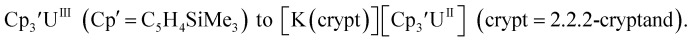
^1^ Subsequently, the tris(cyclopentadienyl) complexes 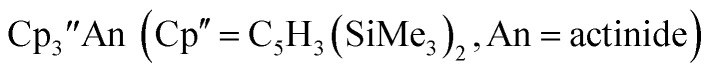 proved to be good precursors for the first examples of crystallographically-characterizable molecular compounds containing Th(ii),^2^ Np(ii),^3–5^ and Pu(ii),^6^eqn (1). Examples of U(ii) are now known in different coordination environments beyond the tris(cyclopentadienyl) ligand sets of eqn (1).^7–9^1
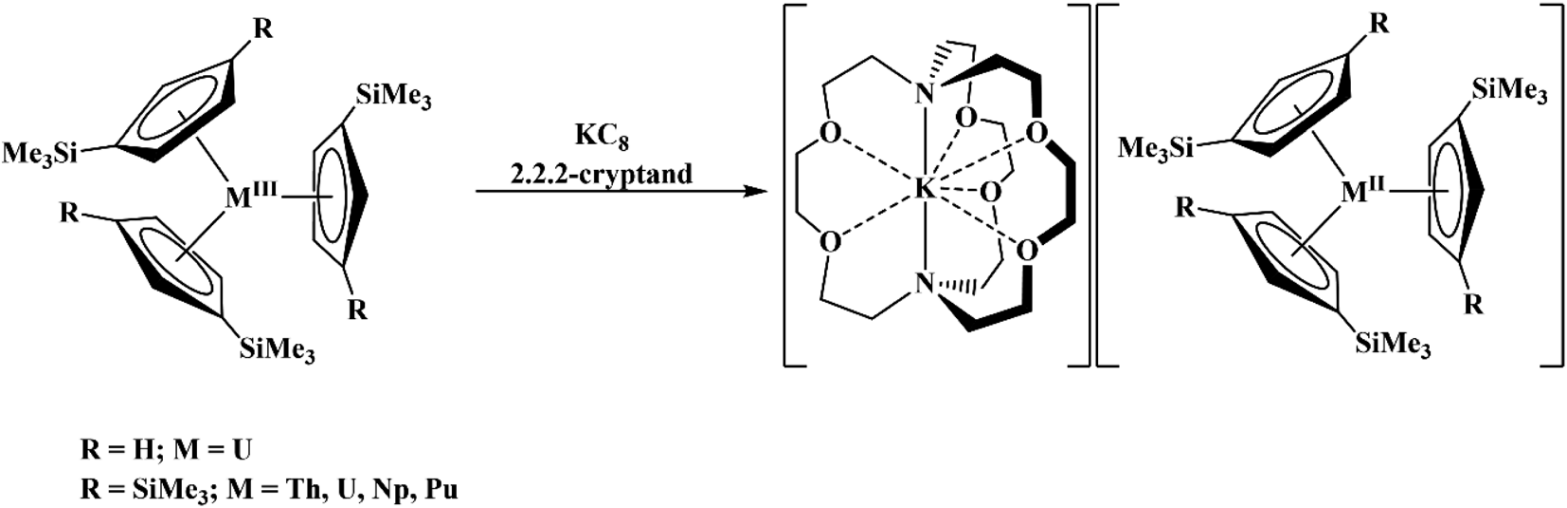


Despite the rapid development of synthetic An(ii) chemistry, there have been few electrochemical studies of these low valent systems, although extensive electrochemistry has been reported for the higher oxidation states of the actinides.^10–13^ This is due in part to the high reactivity of the divalent and trivalent complexes. In addition, actinide electrochemical studies have been challenging because the +3 and +4 metal precursor complexes can react with supporting electrolytes. For example, Inman and Cloke found problems studying (C_5_Me_5_)Th^IV^[C_8_H_6_(SiMe_2_^*t*^Bu)_2_]Cl using [^*n*^Bu_4_N][PF_6_] as supporting electrolyte^14^ as well as with 
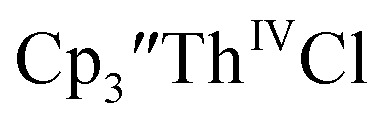
 using [^*n*^Bu_4_N][B(C_6_F_5_)_4_] as supporting electrolyte.^15^

Although electrochemical data have been reported on two U(ii) systems,^9,16^ analogous studies on Th(ii) complexes and on the tris(cyclopentadienyl) systems that led to the first molecular examples of U(ii) have been absent. Meyer and coworkers identified the U(iii)/U(ii) couple in [(^Ad,Me^ArO)_3_mes]U^III^ at −2.495 V *vs.* Fc^+/0^,^16^ that guided synthetic efforts and allowed isolation of [K(crypt)]{[(^Ad,Me^ArO)_3_mes]U^II^}.^7^ More recently, Layfield and coworkers reported the U(iii)/U(ii) couple of (C_5_^i^Pr_5_)_2_U^II^ to be −2.33 V *vs.* Fc^+/0^.^9^ Inman and Cloke studied Th(iv)/Th(iii) redox couples and found that [^*n*^Bu_4_N][BPh_4_] was a good supporting electrolyte for their complexes.^15,17^ Encouraged by their results, we utilized this supporting electrolyte to obtain electrochemical data in this study and on 
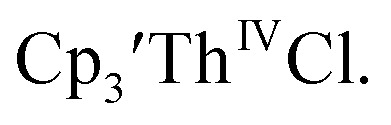
^18^

**Scheme 1:**
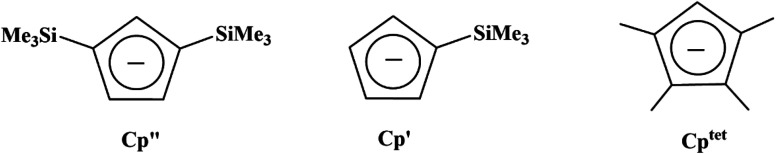
Chemical structures of Cp′′, Cp′, and Cp^tet^ ligands used in this study.

Due to the importance of the tris(cyclopentadienyl) ligand set in the development of low oxidation state actinide chemistry,^19,20^ the electrochemistry of a variety of tris(cyclopentadienyl)uranium and thorium complexes using Cp′′, Cp′, and Cp^tet^ ligands (Cp^tet^ = C_5_Me_4_H), Scheme 1, is reported here as well as the first reported electrochemical measurements on isolated Th(ii) complexes.^2^ Also reported are spectroelectrochemical studies on the Th(ii) compounds that led to the discovery of new synthetic routes to Th(ii) compounds. The results are compared with cyclopentadienyl ligand effects previously examined electrochemically with titanium and zirconium complexes^21^ and with rare-earth metal reaction chemistry.^22–24^

## Results

### Electrochemical protocol

**Fig. 1:**
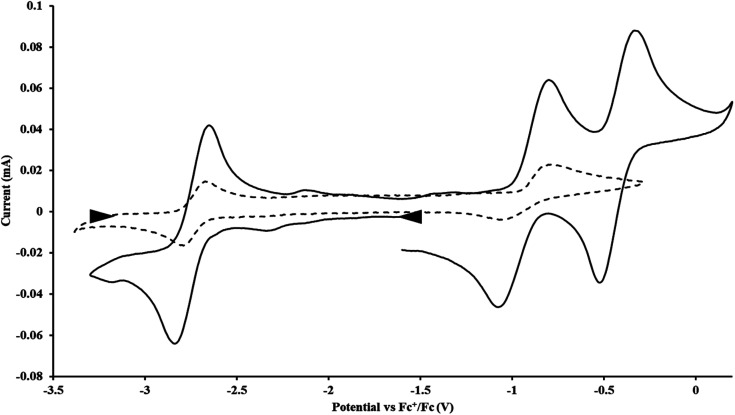
Voltammogram of 4.6 mM 
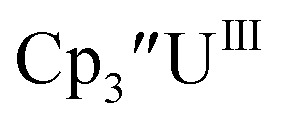
 (solid) and 3.0 mM 
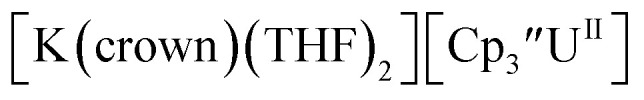
 (dashed) at *ν* = 200 mV s^−1^, in 100 mM [^*n*^Bu_4_N][BPh_4_]/THF. The event centered at −0.495 V is due to internal standard (C_5_Me_5_)_2_Fe.

**Fig. 2:**
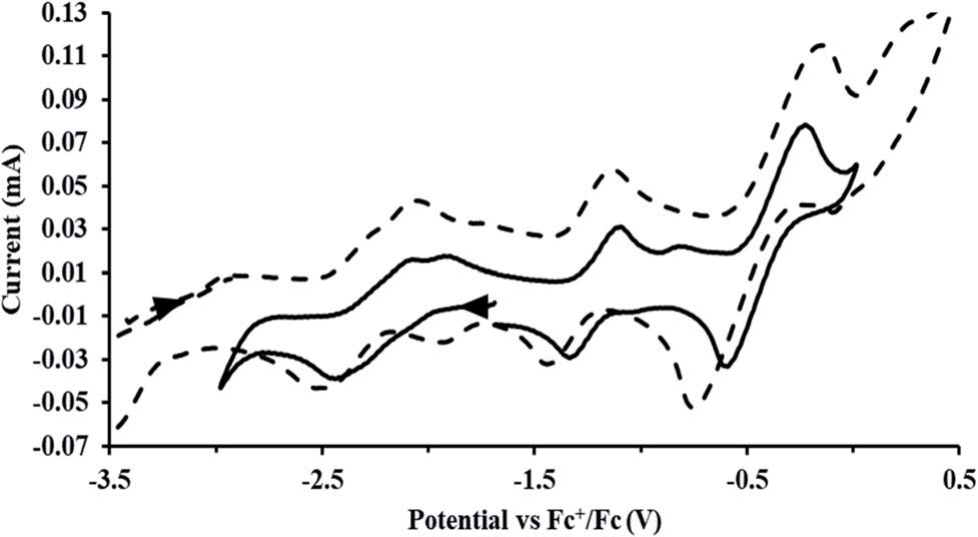
Voltammogram of 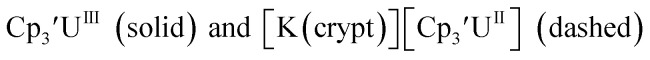 at *ν* = 200 mV s^−1^, in 50 mM [^*n*^Bu_4_N][BPh_4_]/THF. The event centered at −0.495 V is due to internal standard (C_5_Me_5_)_2_Fe.

**Fig. 3:**
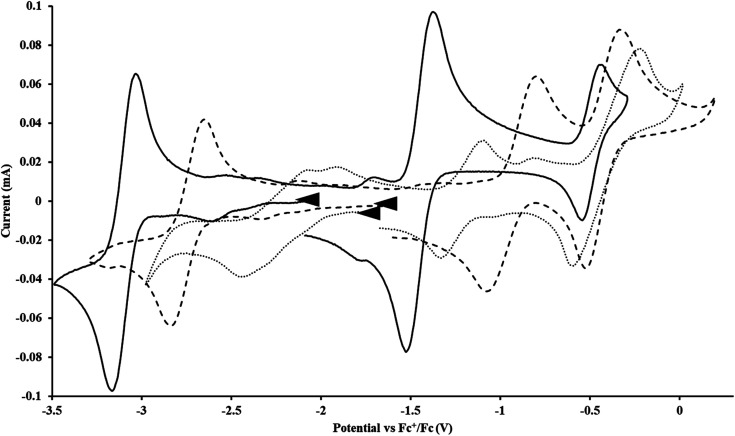
Voltammogram of 7.2 mM Cp^tet^_3_U^III^ (solid, 100 mM [^*n*^Bu_4_N][BPh_4_]/THF) compared to voltammograms of 4.6 mM 
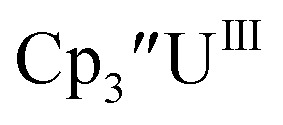
 (dashed, 100 mM [^*n*^Bu_4_N][BPh_4_]/THF) and 11 mM 
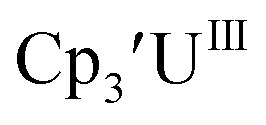
 (dotted, 50 mM [^*n*^Bu_4_N][BPh_4_]/THF) at *ν* = 200 mV s^−1^. The events centered at −0.495 V are due to internal standard (C_5_Me_5_)_2_Fe^II^.

**Fig. 4:**
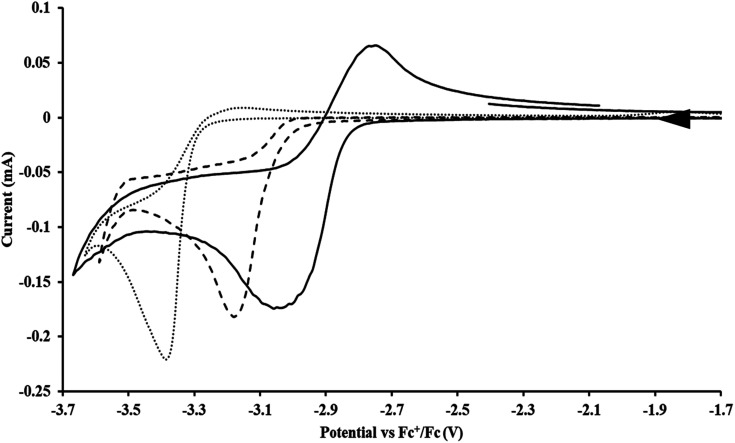
Voltammogram of 7.4 mM 
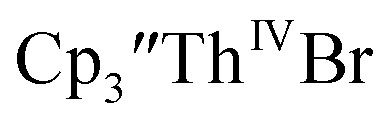
 (solid), 15 mM 
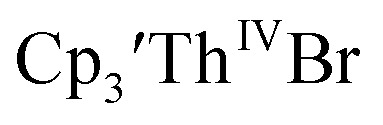
 (dashed), and 12 mM Cp^tet^_3_Th^IV^Br (dotted) at *ν* = 200 mV s^−1^, in 100 mM [^*n*^Bu_4_N][BPh_4_]/THF.

**Fig. 5:**
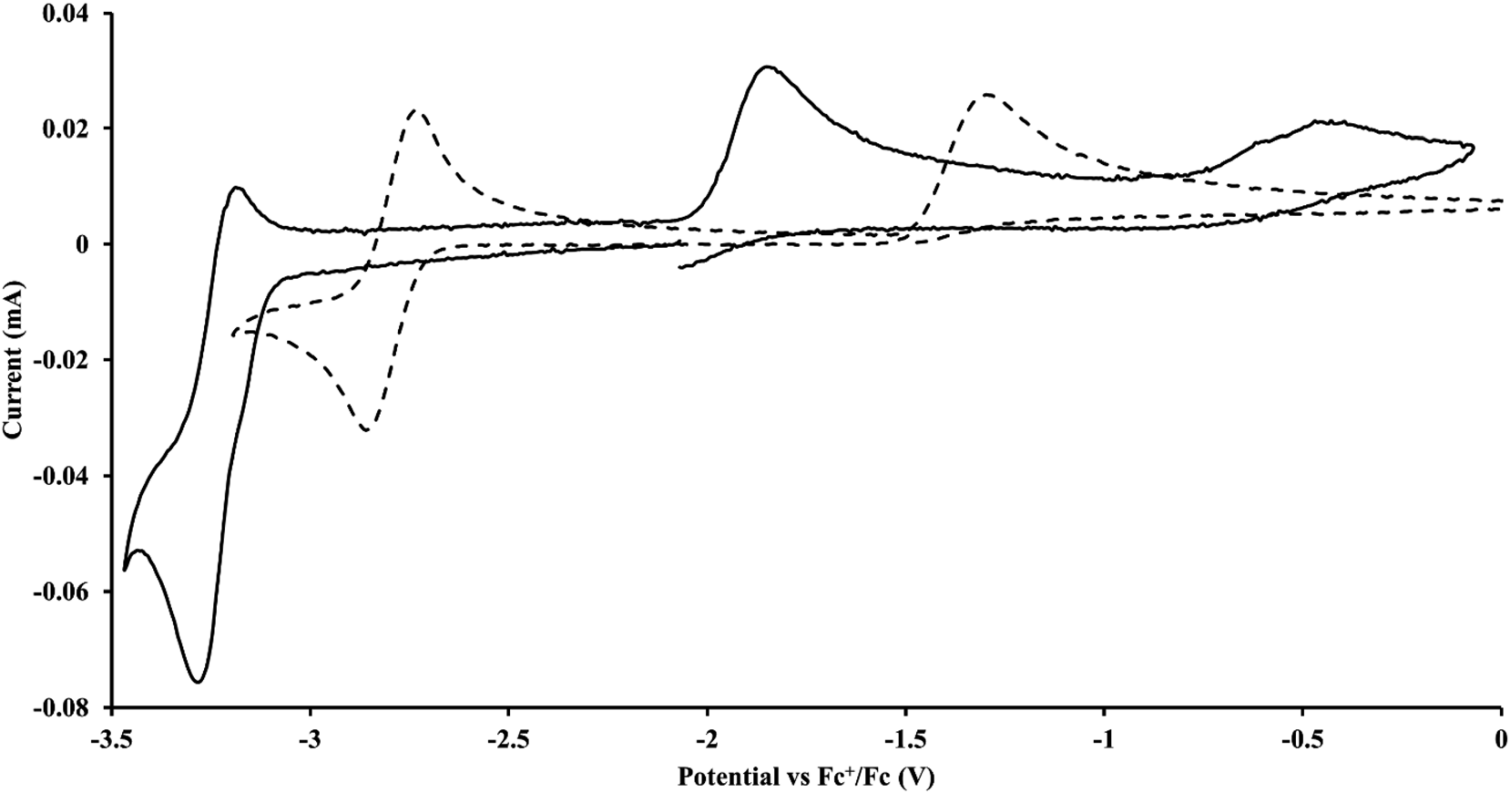
Voltammogram of 4.9 mM 
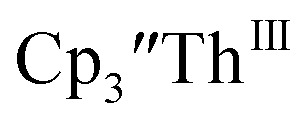
 (solid) at *ν* = 200 mV s^−1^ and 6.7 mM Cp^tet^_3_Th^III^ (dashed) at *ν* = 400 mV s^−1^ in 100 mM [^*n*^Bu_4_N][BPh_4_]/THF.

**Fig. 6:**
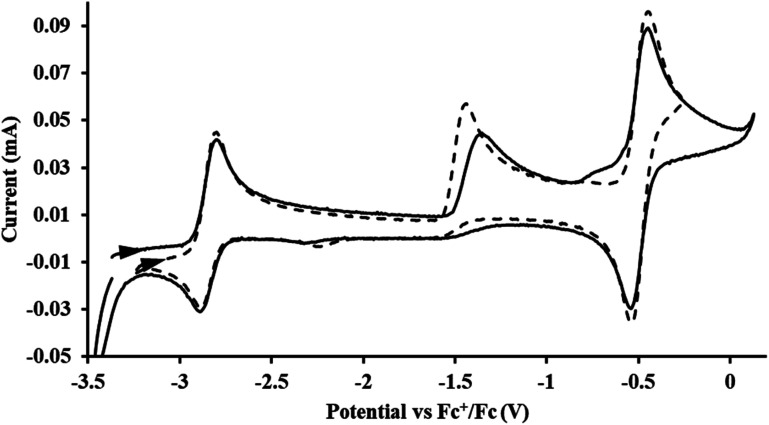
Voltammogram of 4.6 mM 
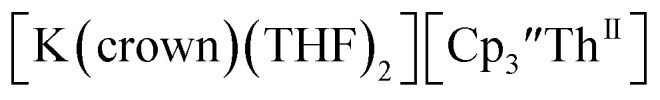
 (solid) and 3.1 mM 
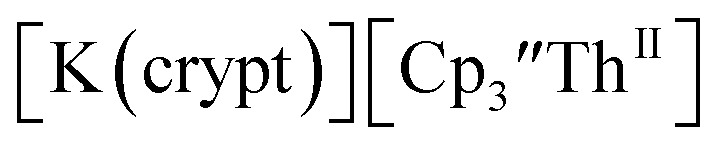
 (dashed) with internal standard (C_5_Me_5_)_2_Fe at *ν* = 200 mV s^−1^ in 100 mM [^*n*^Bu_4_N][BPh_4_]/THF.

All data were collected in THF using 100 mM [^*n*^Bu_4_N][BPh_4_] or 200 mM [^*n*^Bu_4_N][PF_6_] supporting electrolyte concentrations. Both [^*n*^Bu_4_N][BPh_4_] and [^*n*^Bu_4_N][PF_6_] were recrystallized three times prior to use. The low polarity of THF leads to large internal resistance in the electrochemical cell with peak separations over 200 mV often observed.^15,16^ Unless specifically stated, all potentials are referenced to the ferrocenium/ferrocene couple with (C_5_Me_5_)_2_Fe as an internal standard, Fig. S12 and S13.† All electrochemical data were collected with a glassy carbon disc working electrode, platinum wire counter electrode, and silver wire pseudo-reference electrode. All scans were recorded in the cathodic direction except for the isolated U(ii) and Th(ii) compounds which were recorded in the anodic direction. Representative cyclic voltammograms are shown in Fig. 1–6 and complete details are in the ESI.†

### Uranium complexes

Initially, U(iii) complexes known to undergo chemical reduction and oxidation were examined to determine if both the U(iv)/U(iii) and U(iii)/U(ii) redox events could be observed electrochemically. Indeed, both redox couples were observed in the voltammograms for the U(iii) complexes 
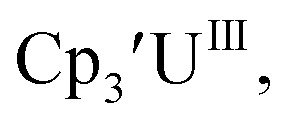
^25^
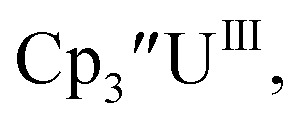
^26^ and Cp^tet^_3_U^III^,^26^ and for the isolated U(ii) complexes 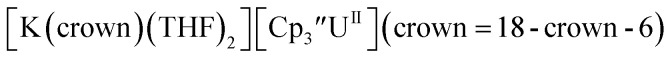
^27^ and 
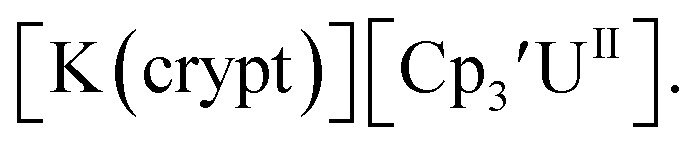
^1^ These values are summarized in Tables 1 and 2 and highlights are described in the following paragraphs.

**Table 1:** Reduction potentials assigned to U(iv)/U(iii) couples in this study and the literature

	*E*_PA_ (V)	*E*_PC_ (V)	U(iv)/U(iii) *E*_1/2_ (V)	Δ*E*_pp_ (C_5_Me_5_)_2_Fe (V)
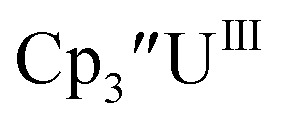	−1.04	−0.83	−0.94^a^	0.20
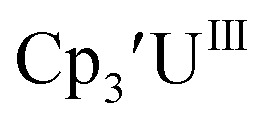	−1.33	−1.20	−1.26^b^	0.36
Cp^tet^_3_U^III^	−1.54	−1.39	−1.46^a^	0.12
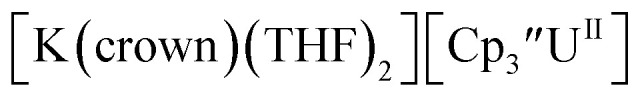	−1.09	−0.37	−0.73^a^	0.15
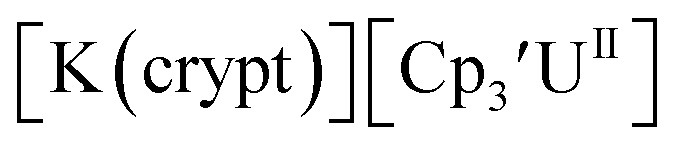	−1.45	−1.12	−1.28^a^	0.57
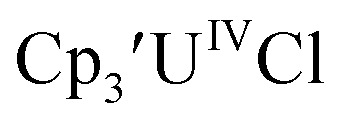			−1.83 (ref. 28)^c^	
(C_5_H_5_)_3_U^IV^Cl			−1.87 (ref. 28 and 29)^c^	
(C_5_MeH_4_)_3_U^IV^Cl			−1.88(ref. 28)^c^	
(C_5_^*t*^BuH_4_)_3_U^IV^Cl			−1.93(ref. 28)^c^	

a 100 mM [^*n*^Bu_4_N][BPh_4_]/THF.

b 50 mM [^*n*^Bu_4_N][BPh_4_]/THF.

c 130 mM [^*n*^Bu_4_N][PF_6_]/THF.

**Table 2:** Reduction potentials assigned to U(iii)/U(ii) couples in this study and the literature

	*E*_PA_ (V)	*E*_PC_ (V)	U(iii)/U(ii) *E*_1/2_ (V)	Δ*E*_pp_ (C_5_Me_5_)_2_Fe (V)
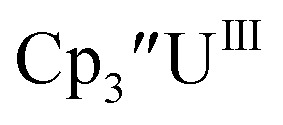	−2.79	−2.67	−2.73^a^	0.20
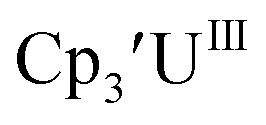	−2.43	−2.08	−2.26^b^	0.36
Cp^tet^_3_U^III^	−3.18	−3.04	−3.11^a^	0.12
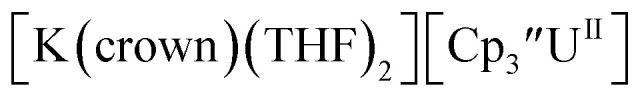	−2.77	−2.65	−2.71^a^	0.15
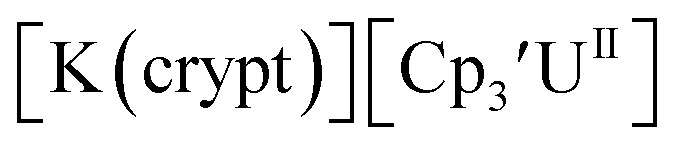	−2.50	−2.03	−2.27^b^	0.57
[(^Ad,Me^ArO)_3_mes]U^III^			−2.495 (ref. 16)^d^	
(C_5_^i^Pr_5_)_2_U^II^			−2.33 (ref. 9)^c^	

a 100 mM [^*n*^Bu_4_N][BPh_4_]/THF.

b 50 mM [^*n*^Bu_4_N][BPh_4_]THF.

c 60 mM [^*n*^Bu_4_N][BPh_4_]/THF.

d 100 mM [^*n*^Bu_4_N][PF_6_]/THF.

#### Cp′′

With the bis(trimethylsilyl)cyclopentadienyl ligand, redox couples assigned to U(iv)/U(iii) and U(iii)/U(ii) are observed at −0.94 V and −2.73 V, respectively, for 
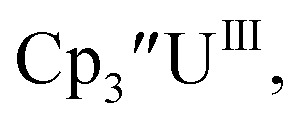
Fig. 1 and S14.† In comparison, the isolated U(ii) complex 
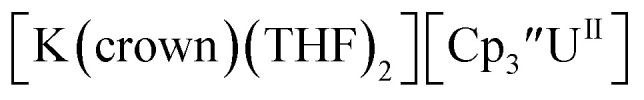
^27^ displays two redox events at −0.73 V and −2.71 V, Fig. 1 and S25.† The *E*_1/2_ values for the U(iii)/U(ii) couple are nearly identical in both systems and the event centered at −2.71 V only appears when scanning anodically for 
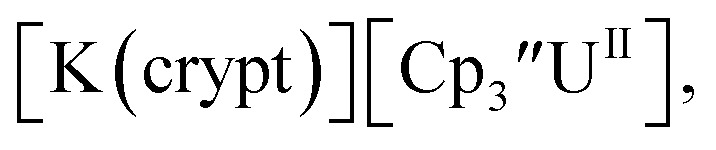
 which supports the assignment as the U(iii)/U(ii) couple.

#### Cp′

Similar reproducible data were obtained with the mono(trimethylsilyl)cyclopentadienyl ligand with U(iv)/U(iii) and U(iii)/U(ii) couples at −1.26 V and −2.26 V, respectively, for 
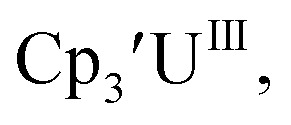
Fig. 2 and S17.† Likewise, the U(iv)/U(iii) and U(iii)/U(ii) couples were observed at −1.28 V and −2.27 V for the U(ii) complex 
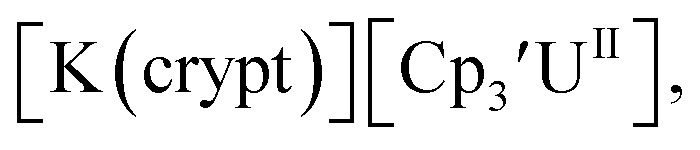
Fig. 2 and S24.† These data were obtained with 50 mM [^*n*^Bu_4_N][BPh_4_] because decomposition occurred at higher electrolyte concentrations. The event at −2.27 V for 
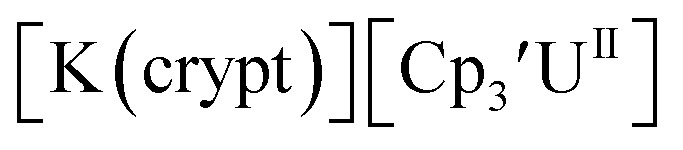
 only appears when scanning anodically. The −2.27 V *E*_1/2_ value for 
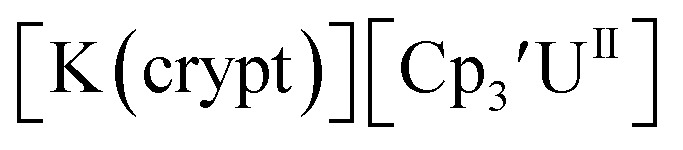
 was less negative than the −2.71 V value for 
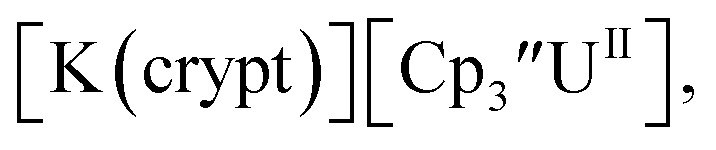
 but it is similar to the two previously reported U(iii)/U(ii) couples for [(^Ad,Me^ArO)_3_mes]U^III^ and (C_5_^i^Pr_5_)_2_U^II^.^9,16^ The minor unassigned events at about −1.9 V in Fig. 2 and S24† attest to the complexity of the system. They were observed across multiple runs and do not disappear after repeated recrystallization of substrate and electrolyte.

#### Cp^tet^

With the tetramethylcyclopentadienyl ligand, the U(iv)/U(iii) and U(iii)/U(ii) couples in Cp^tet^_3_U^III^ were more negative than in 
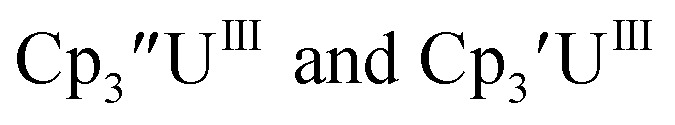
: −1.46 V and −3.11 V, Fig. 3 and S20.† However, data could not be obtained from the isolated U(ii) compound [K(crypt)][Cp^tet^_3_U^II^] because contact with the supporting electrolyte led to immediate decomposition. The voltammogram obtained from the resulting solution displayed at least five redox events, Fig. S29.† This reactivity is consistent with the more strongly reducing nature of the Cp^tet^ complexes as shown by the data in Tables 1 and 2. A third, minor event at −1.7 V was present and cannot be assigned with confidence.

### Thorium complexes

Electrochemical data were collected on all the thorium compounds in this study using both [^*n*^Bu_4_N][PF_6_] and [^*n*^Bu_4_N][BPh_4_] despite multiple reports that electrochemical data on organothorium complexes are difficult to obtain using [^*n*^Bu_4_N][PF_6_].^11,14,15,30–32^ Since the voltammograms do not differ drastically between electrolytes, only the data using [^*n*^Bu_4_N][BPh_4_], Table 3, are discussed below (data with [^*n*^Bu_4_N][PF_6_] are in Table S1†).

**Table 3:** Reduction potentials of tris(cyclopentadienyl)thorium complexes with 100 mM [^*n*^Bu_4_N][BPh_4_] supporting electrolyte

	Th(iv)/Th(iii)			Th(iii)/Th(ii)			
	*E*_PC_ (V)	*E*_PA_ (V)	*E*_1/2_ (V)	*E*_PC_ (V)	*E*_PA_ (V)	*E*_1/2_ (V)	Δ*E*_pp_ Fc (V)
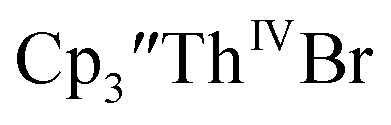	−3.00	−2.77	−2.89				0.14
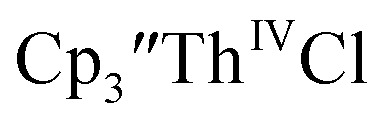	−3.04	−2.81	−2.93				0.22
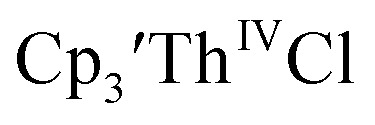	−3.38	−2.90	−3.14				0.16
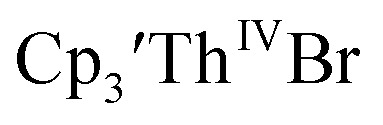	−3.17						
Cp^tet^_3_Th^IV^Br	−3.48	−3.19	−3.34				0.18
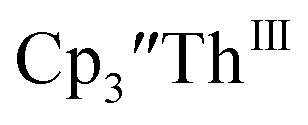				−2.92	−2.78	−2.85	0.19
Cp^tet^_3_Th^III^				−3.33	−3.23	−3.28	0.16
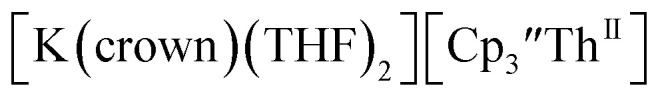				−2.89	−2.79	−2.84	0.09
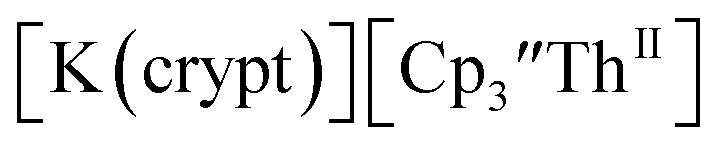				−2.90	−2.81	−2.85	0.09

### Thorium(iv) complexes

#### Cp′′

Initially, 
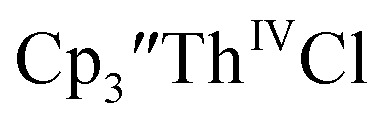
 was examined to compare with the values previously reported by Cloke *et al.*^15^ The cyclic voltammogram of 
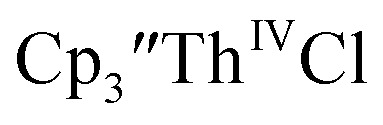
 under our conditions shows the Th(iv)/Th(iii) couple at −2.93 V, Fig. S34,† which is close to the value of −2.96 V reported for 
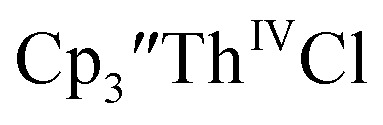
 and 
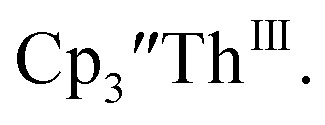
^15^ Similarly, the cyclic voltammogram of 
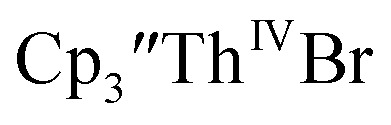
 (ref. 2) shows a Th(iv)/Th(iii) redox couple at −2.89 V, Fig. 4 and S30.† This suggests that the identity of halide does not significantly affect the reduction potential in this system. This is also consistent with bulk synthetic studies that show that 
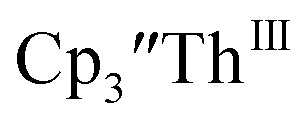
 can be synthesized from both 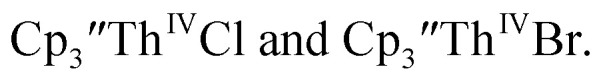
^2,33,34^

#### Cp′ and Cp^tet^


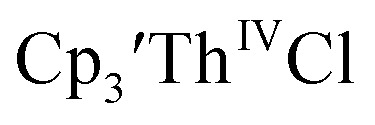
 ^35^ and Cp^tet^_3_Th^IV^Br (ref. 36) were also examined as each these complexes can be chemically reduced to form tris(cyclopentadienyl)Th(iii) species.^18,36^ The cyclic voltammogram of 
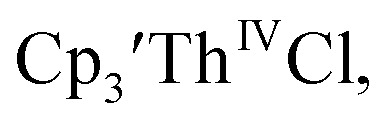
^35^ Fig. S38,† exhibited a cathodic event at −3.14 V that is 0.21 V more negative than that of 
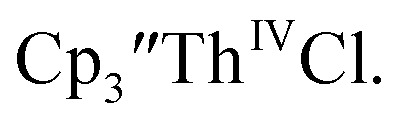
 Similarly, the voltammogram of 
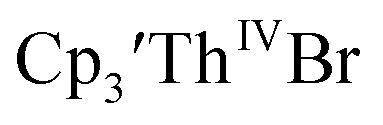
 had a cathodic event at −3.17 V, Fig. 4 and S63.† This event was determined to be a one electron process by comparing the current passed to that of the internal standard, Fig. S65.† The voltammogram of Cp^tet^_3_Th^IV^Br had a cathodic event at −3.48 V, Fig. 4 and S44.† The events in the voltammograms of 
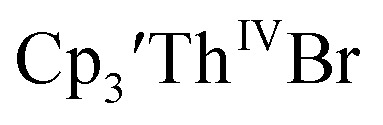
 and Cp^tet^_3_Th^IV^Br are practically irreversible even at scan rates up to 2000 mV s^−1^. These results, along with the uranium studies above in Table 1, clearly show that the reduction potential of the actinide complex trends with the electron donation strength of the ligand in the order of Cp^tet^ > Cp′ > Cp′′.

In addition to the Th(iv)/Th(iii) couple, the voltammograms of the Th(iv) compounds showed an irreversible anodic process that could be a cyclopentadienide oxidation, based on the electrochemical data collected on the cyclopentadienyl salts, KCp′, KCp′′, and KCp^tet^, Fig. S66.† These irreversible anodic events were not found in the uranium systems. This difference in Th and U electrochemistry has been previously observed.^11,15,37,38^ Clearly, the Lewis acidity of the metal influences the potential for these cyclopentadienide oxidations. Cyclopentadienyl rings bound to K^+^, [K(chelate)]^+^, or An^*n*+^ could have different oxidation potentials as evidenced by the differing voltammograms of KCp′′, [K(crown)][Cp′′], and [K(crypt)][Cp′′], Fig. S67.†

### Th(iii) complexes

#### Cp′′

There are fewer Th(iii) options to study since there are only five crystallographically-characterized tris(cyclopentadienyl) Th(iii) complexes, 
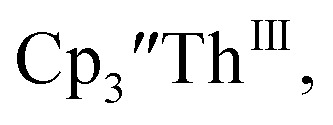
^33,34^ [C_5_H_3_(SiMe_2_^*t*^Bu)_2_]_3_Th^III^,^34^ Cp^tet^_3_Th^III^,^36^ (C_5_^*t*^Bu_2_H_3_)_3_Th^III^,^39^ and (C_5_Me_5_)_3_Th^III^.^40^ Other Th(iii) compounds have been isolated with different ligand environments,^41–45^ but our initial attempts to collect electrochemical data on (C_5_Me_5_)_2_Th^III^[^i^PrNC(Me)N^i^Pr]^44^ led to immediate decomposition. Inman and Cloke found that scanning anodically on 
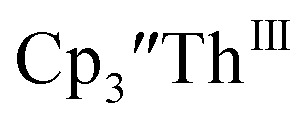
 gave a process at −2.96 V that matched the reduction of 
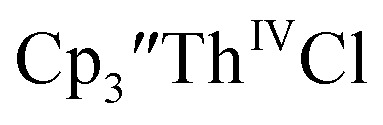
 described above and established the Th(iv)/Th(iii) couple.^15^ In our hands, scanning cathodically on 
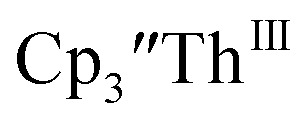
 showed a voltammogram with a redox process centered at −2.85 V, Fig. 5 and S40.† A second cathodic event appears after the first cycle at −2.29 V, or when scanning anodically from the open circuit potential, Fig. S40.† The event at −2.29 V was also observed by Cloke and was attributed to a ligand-based event.

#### Cp′ and Cp^tet^

Since 
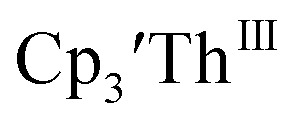
 has only been generated *in situ*,^18^ it was not studied under the present conditions. The voltammogram of Cp^tet^_3_Th^III^ at *ν* = 200 mV s^−1^ displays only a cathodic event, but at *ν* ≥ 400 mV s^−1^, a return oxidation appears and the Th(iii)/Th(ii) redox couple is centered at −3.28 V, Fig. 5 and S48.† This value matches the trend observed for the uranium systems in that Cp^tet^ complexes of thorium are more difficult to reduce than the silyl-cyclopentadienyl analogs. An anodic event at −1.87 V is present and is attributed to a Cp^tet^-based process.

### Th(ii) complexes

The only isolated Th(ii) compounds 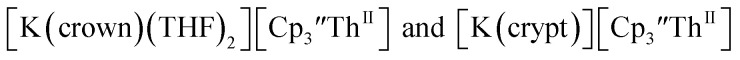 exhibited nearly identical voltammograms. Scanning anodically, 
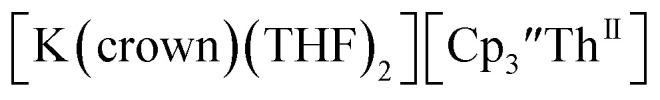
 showed a redox process centered at −2.84 V, which is assigned as the Th(iii)/Th(ii) redox couple, and a second irreversible anodic event at −1.38 V, attributed to ligand-based oxidation, Fig. 6 and S52.† The voltammogram of this Th(ii) compound was practically identical over 5 cycles, Fig. S54.†
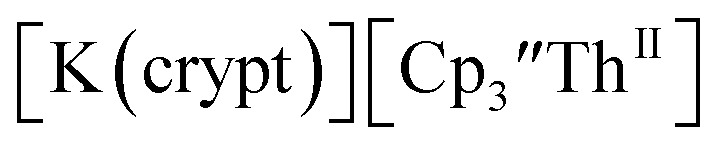
 similarly showed a reversible event centered at −2.85 V and a second anodic event at −1.43 V, Fig. 6, S57 and S61.†

### Thorium spectroelectrochemistry

**Fig. 7:**
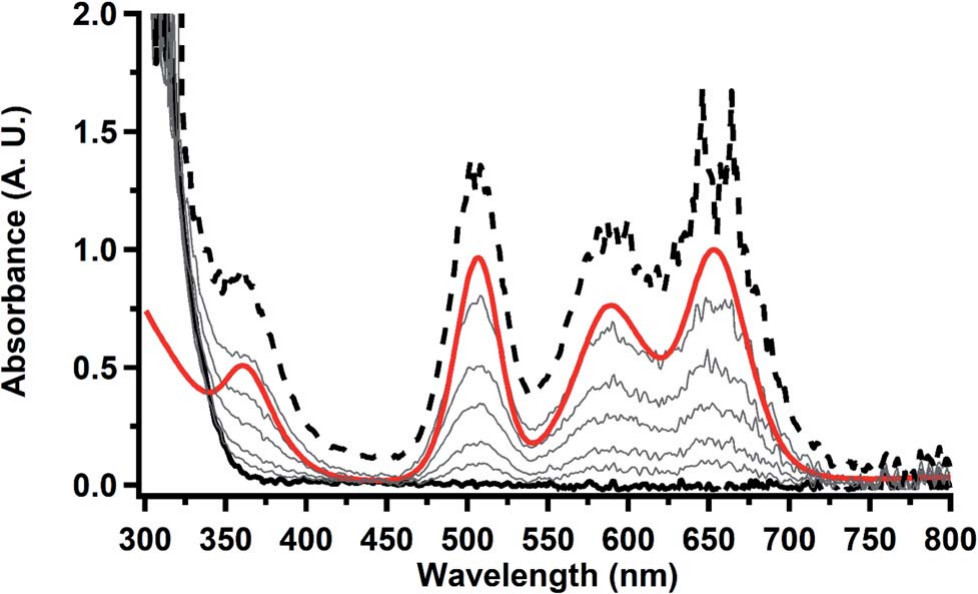
UV-visible spectrum of 
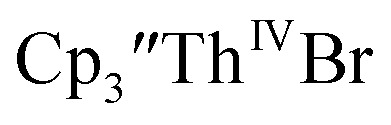
 (black, solid) converting to 
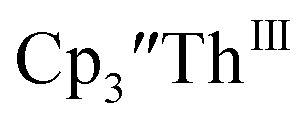
 (black, dashed) during electrolysis at −2.90 V with a starting concentration of 7.0 mM in 200 mM [^*n*^Bu_4_N][PF_6_]/THF. The growth of four bands at 365, 510, 590, and 655 nm is indicative of 
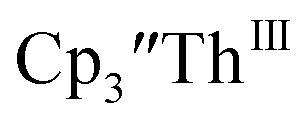
 (red).^34^

The data on isolated 
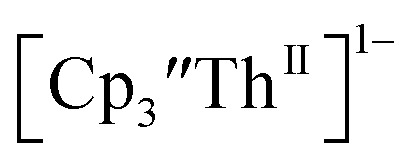
 complexes suggested that the Th(iii)/Th(ii) redox process occurs at about the same potential as the Th(iv)/Th(iii) potential of 
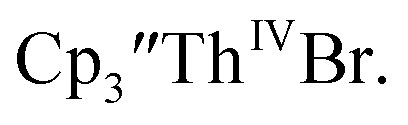
 To investigate this further, spectroelectrochemical UV-visible measurements were obtained. A potential of −2.90 V was applied to a solution of 
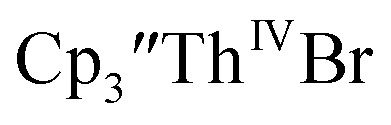
 in 200 mM [^*n*^Bu_4_N][PF_6_]/THF and the UV-visible spectrum was recorded approximately every 5 seconds during electrolysis. The formation of 
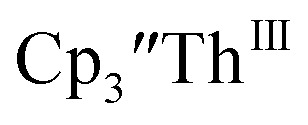
 is clearly shown by the growth of four bands at roughly 360, 500, 580, and 680 nm, Fig. 7, which correspond to the absorption spectrum of 
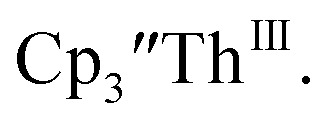
^33,34^ No further reduction to the 
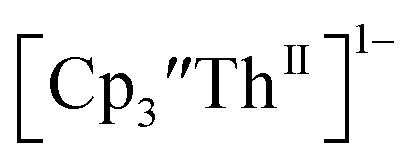
 was observed,^2^ although it cannot be ruled out as the absorbance spectrum reached the maximum of the detector.

**Fig. 8:**
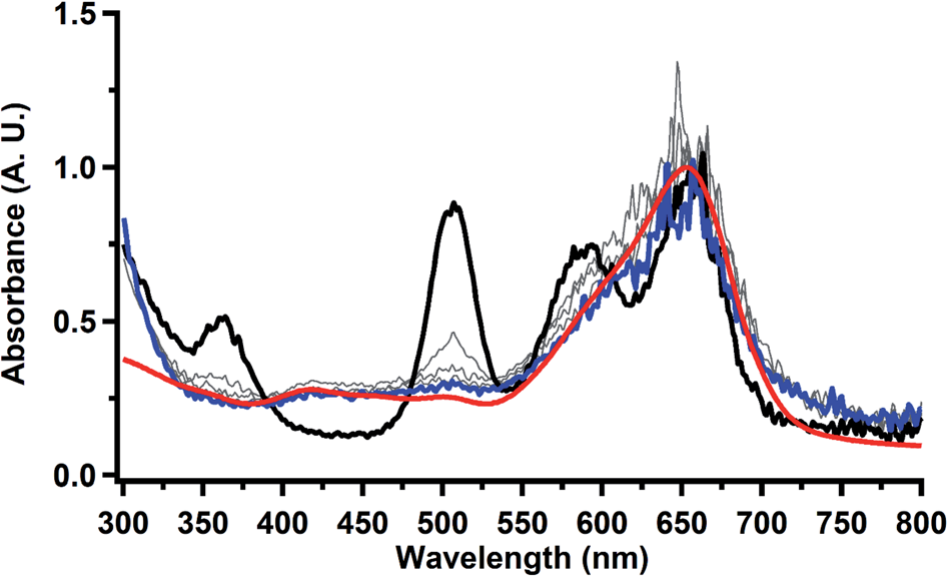
UV-visible spectrum of 
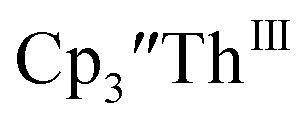
 (black) converting to 
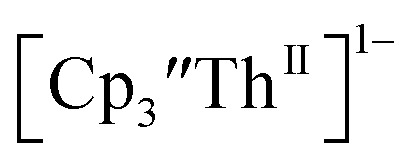
 (blue) during electrolysis at −2.90 V with a starting concentration of 1.1 mM in 200 mM [^*n*^Bu_4_N][PF_6_]/THF. The growth of the band at 650 nm is indicative of 
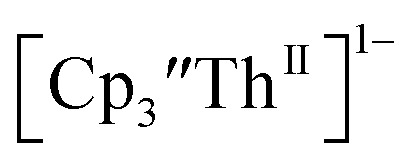
 (red).^2^

Electrolysis of a solution of 
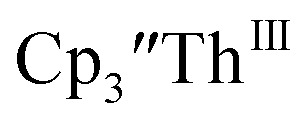
 in 200 mM [^*n*^Bu_4_N][PF_6_]/THF at −2.90 V shows clean conversion to the Th(ii) species 
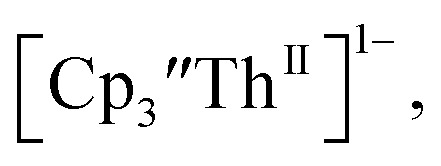
^2^ as indicated by the growth of the large absorption at 650 nm and the concomitant decrease in absorptions at 360, 500, 580, and 680 nm, Fig. 8. Although the absorption spectrum of 
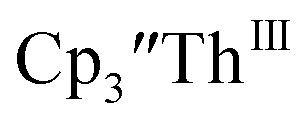
 had disappeared, the absorption at 650 nm, indicative of Th(ii),^2^ decreased in intensity as the electrolysis continued. The Th(ii) species appears to be unstable under the electrolysis conditions.

### Chemical synthesis of Th(ii) complexes from Th(iv) precursors

The similarity of the Th(iv)/Th(iii) couple in 
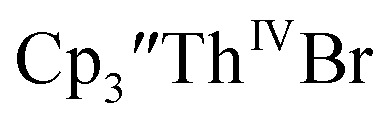
 and Th(iii)/Th(ii) couple in 
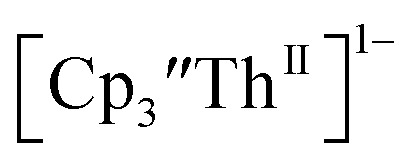
 suggested that Th(iv) compounds could be used as the precursors to Th(ii) compounds as well as the known Th(iii) precursor, 
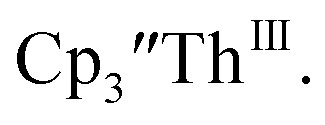
 Indeed, reaction of 2.2 equivalents of KC_8_ to a THF solution of 
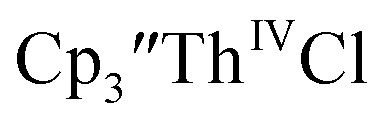
 and crown afforded 
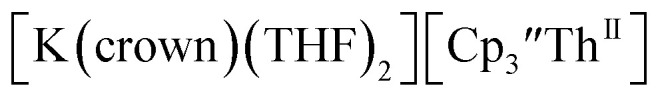
 in 50% crystalline yield, with a significant amount of 
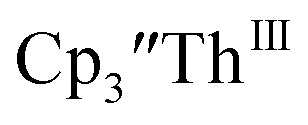
 as a byproduct. Previously, Lappert reported that prolonged stirring of a solution of 
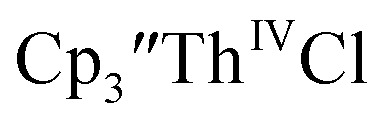
 over excess NaK alloy developed a green color,^34^ which was later confirmed to be the color of Th(ii).^2^

Conversion of Th(iv) to Th(ii) was also studied with 
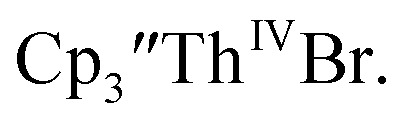
 Reaction of 
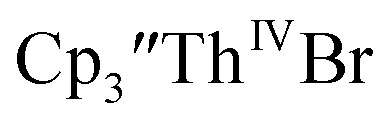
 with 2 equivalents of KC_8_ in THF generated a dark green solution characteristic of Th(ii) within 5 minutes, as did reaction of 
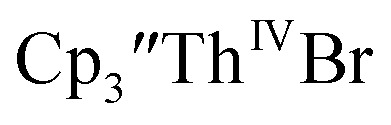
 with excess Na and with excess Li. The UV-visible spectra of these solutions have a strong absorption at 650 nm, identical to the previously reported spectra of 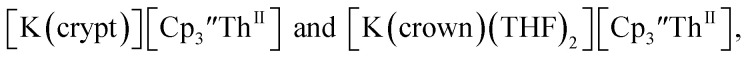
^2^ but the spectra also show a non-negligible amount of 
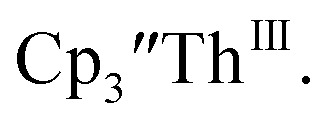
^34^ Formation of the Th(iii) complex is reasonable based on the fact that 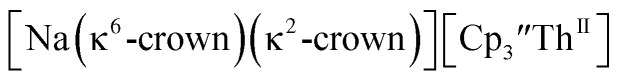 (see below) reacts with 
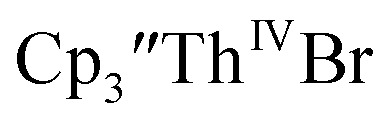
 in THF to immediately form 
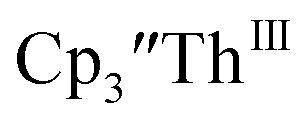
 in near quantitative yield.

**Scheme 2:**
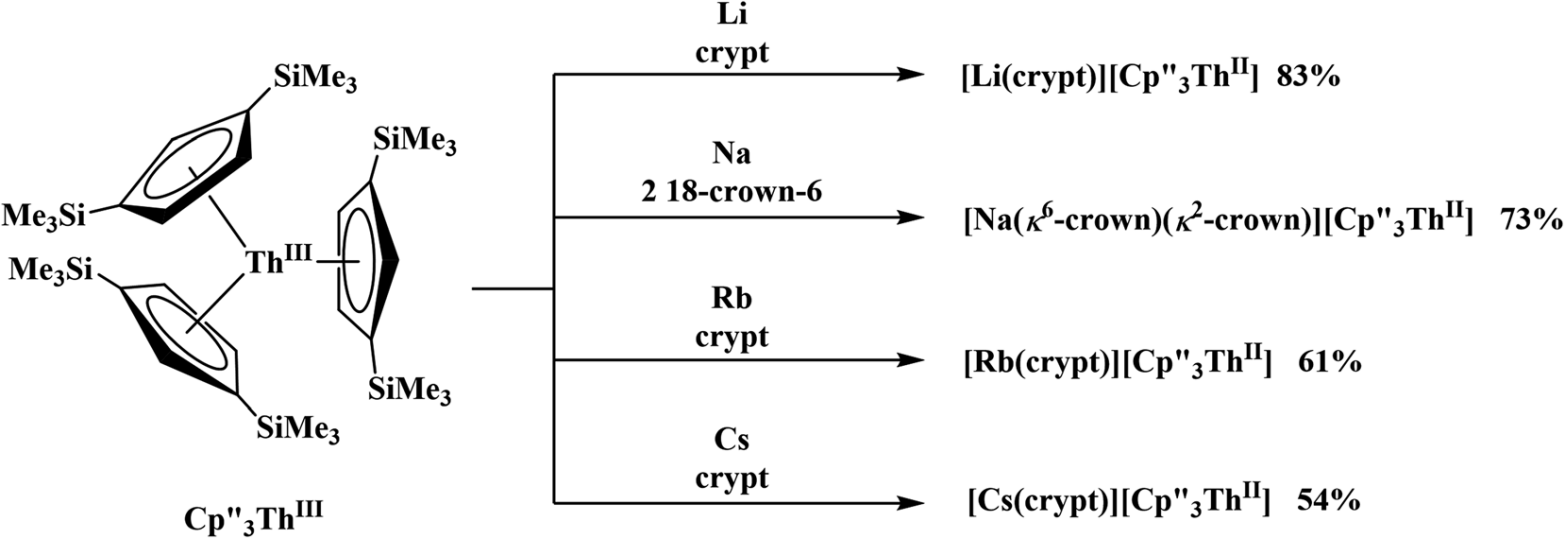
Synthesis of new Th(ii) compounds.

**Fig. 9:**
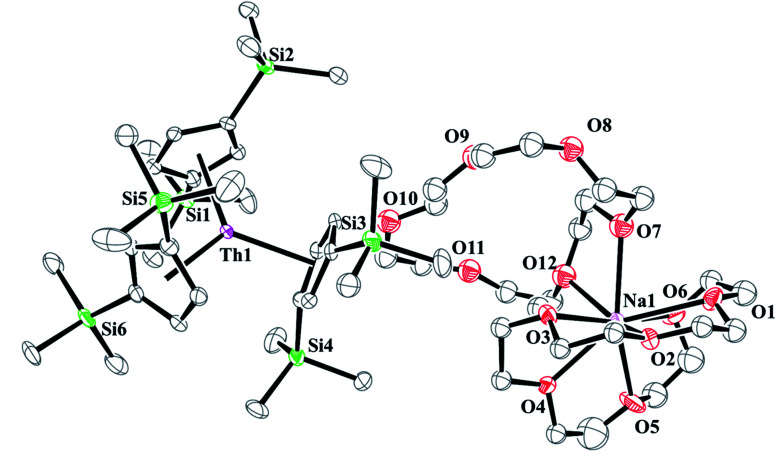
Thermal ellipsoid plot of 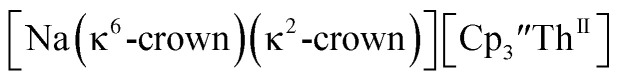 plotted at the 35% probability level. Hydrogen atoms and disorder in the κ^2^-crown unit have been removed for clarity.

These results show that a chelating agent is not necessary for the chemical synthesis of Th(ii) species in solution. However, the chelating agent appears necessary for efficient separation of the Th(ii) product from the Th(iii) starting material, as pure samples of 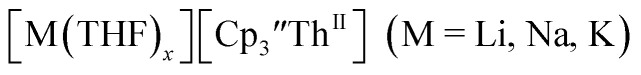 were not isolated even though it is possible to isolate chelate-free examples of 
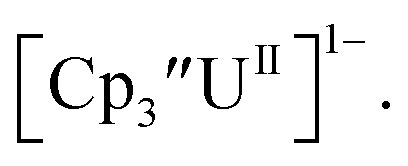
^46^ Further support for the importance of alkali metal chelates is that addition of 18-crown-6 to the reaction of 
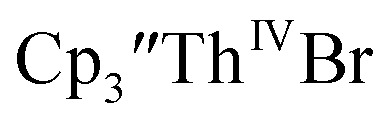
 and excess Na provided X-ray quality crystals that were identified as 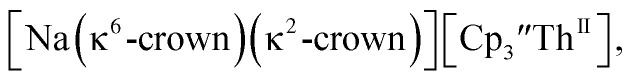 only the third reported crystal structure of a Th(ii) complex, Scheme 2, Fig. 9.

Similarly, the reaction of 
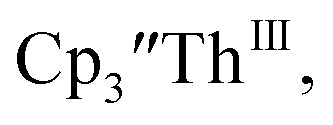
 Rb, and crypt in THF afforded dichroic blue/red crystals of 
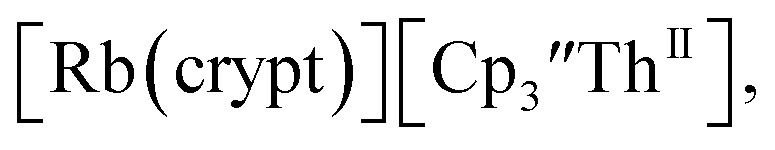
 isolated in 61% crystalline yield and identified by X-ray crystallography, Scheme 2, Fig. S69.† In addition, the reaction of 
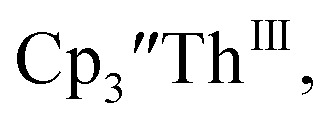
 Cs, and crypt afforded dark blue/red crystals of 
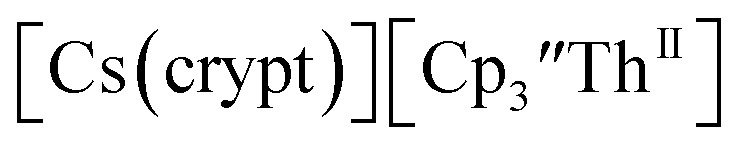
 in 54% crystalline yield, Scheme 2, Fig. S70.† The [Rb(crypt)]^1+^ and [Cs(crypt)]^1+^ compounds are isomorphous with the [K(crypt)]^1+^ analog^2^ and can be easily separated from the 
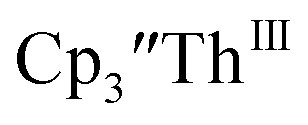
 starting material, which was difficult without the use of a chelate. The reaction of 
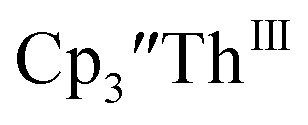
, Li, and crypt formed dark blue-green needles of 
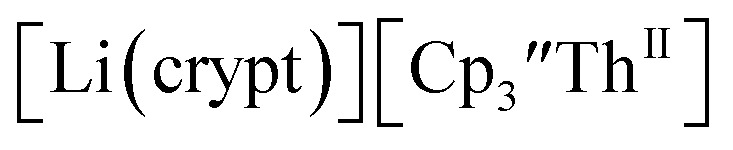
 in 83% yield, but the crystals were not suitable for X-ray diffraction, Scheme 2.

Since the reaction chemistry and the spectroelectrochemistry suggested that the Th(ii) complexes were generated from a Th(iv) precursor through a Th(iii) intermediate, reactions with the two-electron reductant Ba were studied. The Ba(ii)/Ba(0) reduction potential is nearly identical to that of K(i)/K(0).^47^ Surprisingly, prolonged stirring of a THF solution of 
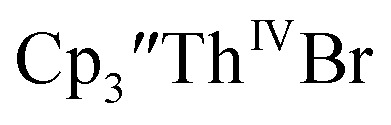
 and excess Ba afforded only 
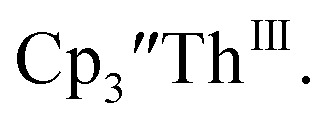
 When chelates were added, the reaction of 
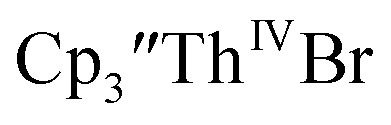
 and crown or 
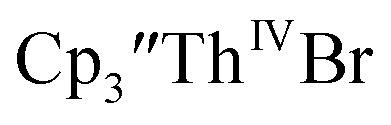
 and crypt over excess Ba formed 
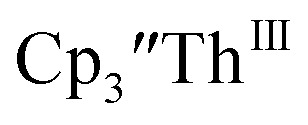
 and then the dark green color of Th(ii) with UV-visible spectra consistent with 
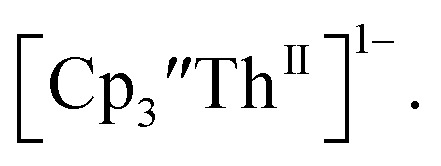
 Addition of elemental Hg did not appear to affect the rate of formation of the Th(ii) species. These results, coupled with the spectroelectrochemical measurements, strongly suggest that the Th(iv)/Th(ii) redox couple is not observed experimentally in these systems and that instead two one-electron processes occur.

## Discussion

### An(iv)/An(iii) processes

The trends observed in the U(iv)/U(iii) and Th(iv)/Th(iii) redox couples in Tables 1–3 indicate that Cp^tet^ is more electron donating than Cp′, which is more electron donating than Cp′′. This follows the electron-donating ability of the ligands previously found in studies of (C_5_R_5_)_2_Zr(CO)_2_ complexes^21^ and yttrium compounds.^22,24^ For the zirconium complexes, the CO stretching frequency and the reduction potentials were analyzed to determine electron-donation strength of the cyclopentadienyl ligand. Generally in these An(iv)/An(iii) studies, the thorium complexes showed less reversible processes than the uranium compounds. In the 
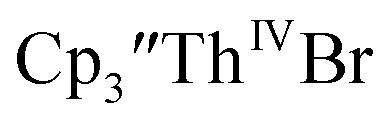
 case, UV-visible spectroelectrochemistry measurements show that this compound is reduced under electrochemical conditions to 
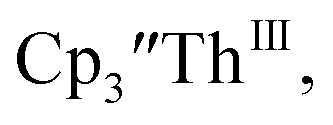
 which requires loss of Br^1−^ and geometric reorganization. In the 
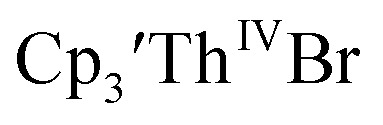
 case, density functional theory calculations have shown that the putative initial reduction product, 
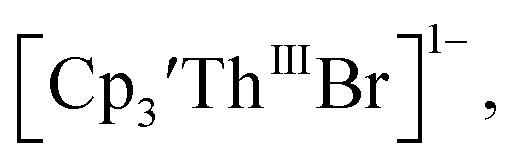
 would be unstable with respect to 
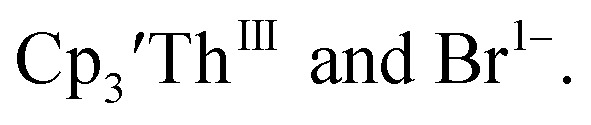
^18^ These results are consistent with the electrochemical irreversibility of the system.

### An(iii)/An(ii) processes

To our knowledge, only two other U(iii)/U(ii) couples have been assigned *via* electrochemistry: [(^Ad,Me^ArO)_3_mes]U^III^ at −2.495 V using [^*n*^Bu_4_N][PF_6_]^16^ and (C_5_^i^Pr_5_)_2_U^II^ at −2.33 V using [^*n*^Bu_4_N][BPh_4_].^9^ The −2.26 V value for 
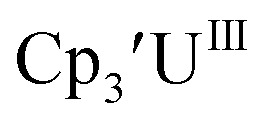
 matches well with these two data points, even though 
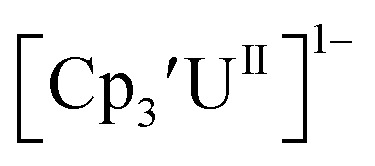
 and (C_5_^i^Pr_5_)_2_U^II^ have been assigned 5f^3^6d^1^ electron configurations,^1,9^ while {[(^Ad,Me^ArO)_3_mes]U^II^}^1−^ is best described as 5f^4^.^7^ The −2.73 V reduction potential for 
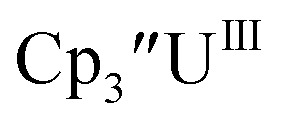
 is unexpectedly more reducing than those of these other three complexes. This is also unusual in that solutions of 
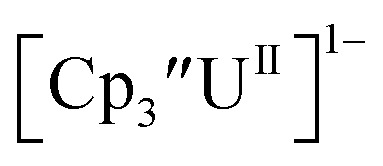
 have longer lifetimes than solutions of 
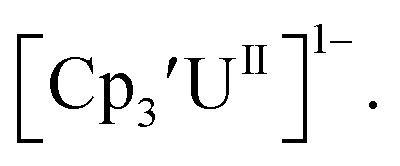
^27^ The U(iii)/U(ii) reduction potential for Cp^tet^_3_U^III^ was determined to be −3.11 V, which is the most negative reduction potential for these compounds and matches the trend observed for the An(iv)/An(iii) couples.

Th(ii) complexes were investigated for the first time *via* electrochemistry and the *E*_1/2_ values for the Th(iii)/Th(ii) couple observed in the isolated Th(ii) compounds matched the value observed in 
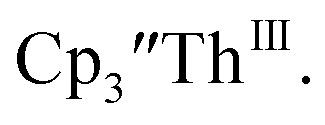
 Surprisingly, the Th(iv)/Th(iii) couple of 
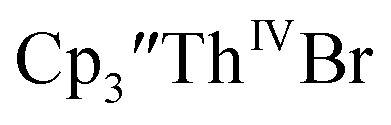
 appears to be about the same as the value for the Th(iii)/Th(ii) couple of 
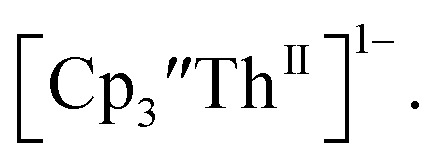
 This result was tested chemically and it was found that reduction of Th(iv) with excess reducing agent would form Th(ii) compounds directly with KC_8_, Na, Li, and Ba both with and without the use of a chelating agent. Blue 
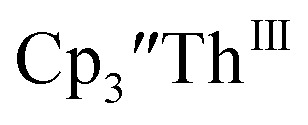
 is observed as an intermediate in these reactions which indicates formation of the Th(ii) products arises from two one-electron reductions. Furthermore, the *E*_1/2_ values for Th(iii)/Th(ii) match the expected trend compared to uranium based on previously calculated An(iii)/An(ii) reduction potentials.^48–50^

The thorium electrochemistry was also unusual in that electrochemical data were obtained using [^*n*^Bu_4_N][PF_6_] as supporting electrolyte on isolated Th(iv), Th(iii), and Th(ii) compounds. This electrolyte has proven to be more reactive than [^*n*^Bu_4_N][BPh_4_] with some complexes^11,15^ and it may have been expected that Th(ii) would react with it. The fact that the Th(iii)/Th(ii) reduction potentials vary slightly depending on the specific electrolyte highlights the fact the reduction potentials of these systems are very sensitive to experimental conditions.

## Conclusion

Electrochemical data on three series of tris(cyclopentadienyl) An(iv), An(iii), and An(ii) (An = Th, U) complexes, including the first data on Th(ii) complexes, complimented by UV-visible spectroelectrochemical measurements, show a direct correlation between reduction potential and the electron-donating ability of the cyclopentadienyl ring. The studies indicate that Th(iii) is a stronger reductant than U(iii), but the reduction potential of U(ii) is similar to that of Th(ii). Two unexpected results should stimulate further studies. The U(iii)/U(ii) reduction potential of 
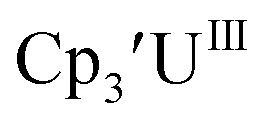
 is similar to the two previously reported U(iii)/U(ii) values, but it is significantly less negative than the Cp′′ analog. The reduction potentials of Th(iv)/Th(iii) and Th(iii)/Th(ii) couples are sufficiently similar that Th(ii) complexes can be made directly from Th(iv) precursors without the need to isolate the Th(iii) intermediate.

## Author contributions

J. C. W. synthesized all compounds and performed the cyclic voltammetry experiments. J. C. W. and J. M. B. performed the spectroelectrochemistry experiments. J. W. Z. analyzed the X-ray diffraction data. All authors analyzed the electrochemistry data. J. C. W. and W. J. E. wrote the manuscript with input from all authors.

## Conflicts of interest

There are no conflicts to declare.

## Supplementary Material

SC-012-D1SC01906F-s001

SC-012-D1SC01906F-s002
