# Supplementary material for: Electrochemical studies of tris(cyclopentadienyl)thorium and uranium complexes in the +2, +3, and +4 oxidation states
Source: Chem Sci. 2021 May 7;12(24):8501–11. doi: 10.1039/d1sc01906f (PMC8221189; doi:10.1039/d1sc01906f)
Supplement: SC-012-D1SC01906F-s001 [file SC-012-D1SC01906F-s001.pdf]

Electronic Supplementary Material (ESI) for Chemical Science.
This journal is © The Royal Society of Chemistry 2021

Supporting Information for:

Electrochemical Studies of Tris(cyclopentadienyl) Thorium and Uranium Complexes in the +2, +3, and +4 Oxidation States

Justin C. Wedal, Jeffrey M. Barlow, Joseph W. Ziller, Jenny Y. Yang,* and William J. Evans*

Department of Chemistry, University of California, Irvine, California 92697, United States

Email: j.yang@uci.edu, wevans@uci.edu

*To whom correspondence should be addressed

Table of Contents

Experimental Details S4

UV-Visible spectra of [Li(crypt)][Cp″$_3$Th], [Na(crown)$_2$][Cp″$_3$Th],

[Rb(crypt)][Cp″$_3$Th], and [Cs(crypt)][Cp″$_3$Th] in THF S10

NMR Spectra of [Li(crypt)][Cp″$_3$Th], [Na(crown)$_2$][Cp″$_3$Th],

[Rb(crypt)][Cp″$_3$Th], and [Cs(crypt)][Cp″$_3$Th] S11

Electrochemical data for…

$(C_5H_5)_2$Fe and $(C_5Me_5)_2$Fe under present conditions S22

Cp″$_3$U S23

Cp′$_3$U S24

Cp$^{tet}_3$U S26

[K(crypt)][Cp′$_3$U] S28

[K(crown)(THF)$_2$][Cp″$_3$U] S29

[K(crypt)][Cp$^{tet}_3$U] S31

Cp″$_3$ThBr S32

Cp″$_3$ThCl S34

Cp′$_3$ThCl S35

Cp″$_3$Th S37

Cp$^{tet}_3$ThBr S39

Cp$^{tet}_3$Th S41

[K(crown)(THF)$_2$][Cp″$_3$Th] S43

[K(crypt)][Cp″$_3$Th] S46

KCp^{tet}, KCp, KCp′, KCp″, [K(crown)][Cp″], [K(crypt)][Cp″] **S50**

Crystallographic Data for…

[K(crown)][Cp″] **S53**

[Na(crown)$_2$][Cp″$_3$Th] **S60**

[Rb(crypt)][Cp″$_3$Th] **S68**

[Cs(crypt)][Cp″$_3$Th] **S76**

References and Definitions **S84**

Experimental Details

Caution! ^{232}Th and ^{238}U are α emitters with half-lives of approximately 1.41×10^{10} and 4.47 x 10^9 years, respectively. Samples should be prepared and handled only in laboratories appropriately equipped to handle radioactive materials.

All syntheses and manipulations were conducted under an Ar atmosphere with rigorous exclusion of air and water using standard glovebox and vacuum line techniques. Solvents were sparged with UHP argon and dried by passage through columns containing Q-5 and molecular sieves prior to use. Deuterated NMR solvents were dried over NaK alloy, degassed by three freeze-pump-thaw cycles, and vacuum transferred prior to use. NMR spectra were recorded on an AVANCE600 MHz spectrometer at 298 K and referenced to residual proteo-solvent resonances. Cp'_3U,[1] $[K(crypt)][Cp'_3U]$,[1] Cp''_3U,[2] $[K(crown)(THF)_2][Cp''_3U]$,[2] Cp^{tet}_3U,[3] $U(NR_2)_3$,[4] Cp''_3ThBr,[5] Cp'_3ThCl,[6] Cp^{tet}_3ThBr,[7] Cp''_3Th,[5,8] Cp^{tet}_3Th,[7] $[K(crown)(THF)_2][Cp''_3Th]$,[9] $[K(crypt)][Cp''_3Th]$,[9] KCp^{tet},[3] KCp',[10] and KCp''[10] were synthesized according to literature procedures. 18-crown-6 (Alfa Aesar) was sublimed at 30 °C at 10^{-5} Torr before use. 2.2.2-cryptand (Aldrich) was dried under vacuum at 10^{-5} Torr before use. Electrochemical grade (>99%) $[^nBu_4N][BPh_4]$ (Sigma) and electrochemical grade (>99.9%) $[^nBu_4N][PF_6]$ (Sigma) were recrystallized from acetone three times and dried at 80 °C and 10^{-5} Torr overnight before use. $(C_5Me_5)_2Fe$ was purified by sublimation before use.

All actinide compounds were purified by recrystallization and dried before data collection. Electrochemical measurements were collected with a freshly made THF solution of supporting electrolyte with a glassy carbon working electrode, platinum wire counter electrode, and silver wire pseudo-reference electrode with a Princeton Applied Research PARSTAT 2273 Advanced Electrochemical System and referenced with internal standard $(C_5Me_5)_2Fe$. Internal resistance was

measured for each solution and resistance was manually compensated by approximately 90% of the measured value. All scans were measured in the cathodic direction except for the isolated U(II) and Th(II) complexes and KC_5R_5 compounds which were measured in the anodic direction. UV-visible spectroelectrochemical measurements were made using a Pine Instruments UV-visible kit with a Pt working and counter electrode and Ag wire pseudo-reference and an Agilent Cary 60 UV-visible spectrophotometer fitted with an Agilent fiber optic coupler connected to an Ocean Optics CUV 1 cm cuvette holder inside the glovebox. UV-visible measurements were made using an Agilent Cary 60 spectrophotometer in THF in a 1 mm cuvette.

Although the highest purity of commercially available [^{n}Bu$_4$N][BPh$_4$] was used, it reacted with some actinide compounds. The Th(IV) compounds $Cp'_3Th^{IV}Cl$[11] and $Cp^{tet}_3Th^{IV}Br$[7] showed no noticeable decomposition while in the presence of this material, but purple $Cp^{tet}_3Th^{III}$ [7] immediately decomposed to a yellow solution and brown $Cp^{tet}_3U^{III}$ [12] turned orange when added to commercial [^{n}Bu$_4$N][BPh$_4$] in THF. Hence, multiple recrystallizations of the commercial electrolyte were required until no reaction was observed with the actinide complexes and reproducible data were obtained. Fresh electrolyte solutions were made immediately before data collection, as small amounts of precipitate formed if the electrolyte solution sat for an extended period of time, even overnight. These samples caused decomposition with some actinide samples upon mixing. Small events were present in the voltammograms of Cp'_3U and Cp^{tet}_3U that are attributed to either decomposition or impurities in the sample, despite recrystallization immediately prior to data collection. These events were present across multiple runs with different batches of material. The solubility limit of [^{n}Bu$_4$N][BPh$_4$] in THF was roughly 100 mM which is the concentration used for most experiments. Exceptions are Cp'_3U^{III} and [K(crypt)][Cp'_3U^{II}] in which 50 mM concentrations were used since the compounds appeared to decompose in higher

concentration solutions. [^{n}Bu$_4$N][PF$_6$] was used at a concentration of 200 mM to determine if peak separations would be smaller than 100–200 mV. They were not.

General Electrochemistry Procedure. Inside the glovebox, a stock electrolyte solution was freshly prepared in THF. Between 1–2 mL of this solution was transferred to a 20 mL scintillation vial and a voltammogram of this solution was collected to verify the electrolyte solution was free of impurities. Roughly 10–15 mg of actinide compound was dissolved in the same electrolyte solution to yield approximately a 5 mM solution. Electrodes were placed into the vial and the vial was left open to the box atmosphere during data collection. The internal resistance was measured and cyclic voltammetry experiments were recorded. Decamethylferrocene, $(C_5Me_5)_2Fe$ was added to the same solution following all data collection, and a single scan was recorded to measure the internal standard redox event.

Synthesis of [Li(crypt)][Cp″$_3$Th]. Cp″$_3$Th (50 mg, 0.058 mmol) and crypt (23 mg, 0.061 mmol) were dissolved in THF (1 mL) and transferred to a vial containing a Li smear (~5 mg) and placed in the freezer at −35 °C overnight. The inky blue/green solution was filtered and dried under vacuum. The solids were dissolved in Et_2O (3 mL) and layered under hexane at −35 °C. Dark blue needles grew overnight (60 mg, 83%). ^{1}H NMR (THF-d_8): δ 5.10 (s, 9H, $C_5H_3R_2$), 3.66 (m, 11H, crypt), 3.57 (m, 14H, crypt) (overlapping with THF), 2.68 (m, 11H, crypt), 0.60 ppm (s, 42H, SiMe_3). ^{13}C (THF-d_8): δ 119.5 ($C_5H_3R_2$), 114.6 ($C_5H_3R_2$), 113.0 ($C_5H_3R_2$), 71.2 (crypt), 69.2 (crypt), 54.6 (crypt), 1.5 ppm (SiMe_3). ^{7}Li NMR (THF-d_8): δ −1.19 ppm. IR: 2943m, 2881m, 1233s, 1168s, 1071s, 910s, 820s, 743s, 675s cm^{-1}. UV-visible (THF): 657 nm (15,000 M^{-1}cm^{-1}). Anal Calcd for $C_{51}H_{99}N_2O_6Si_6ThLi$: C 49.25, H 8.02, N 2.25. Found: C 43.76, H 7.09, N 1.68. Low values were observed across multiple runs and suggests incomplete combustion

which has been problematic for high silicon-containing actinide species.[1,2,5,9,13] The calculated C:H:N ratio of $C_{51}H_{98.5}N_{1.5}$ is close to the expected value.

Synthesis of [Na(crown)$_2$][Cp″$_3$Th]. Cp″$_3$Th (48 mg, 0.056 mmol) and 18-crown-6 (28 mg, 0.11 mmol) were dissolved in THF (1 mL) and transferred into a vial containing a Na (22 mg, 0.96 mmol) smear along the wall. The vial was placed in the freezer at −35 °C overnight. The inky blue/green solution was filtered and dried under vacuum. The solids were dissolved in Et_2O (3 mL) and layered under hexane at −35 °C. Dark blue crystals suitable for X-ray diffraction grew overnight (58 mg, 73%). ^{1}H NMR (THF-d_8): δ 4.42 (s, 9H, $C_5H_3R_2$), 3.58 (s, 35H, OCH_2CH_2O) 0.18 ppm (s, 54H, Si*Me*$_3$). ^{13}C (THF-d_8): δ 120.07 ($C_5H_3R_2$), 115.33 ($C_5H_3R_2$), 114.22 ($C_5H_3R_2$), 70.64 (OCH_2CH_2O), 1.76 ppm (Si*Me*$_3$). IR: 2943m, 2886m 1352m, 1233s, 1169s, 1105s, 1072s, 965m, 910s, 822s, 783s, 742m, 673m cm^{-1}. UV-visible (THF): 658 nm (12,000 M^{-1}cm^{-1}). Anal Calcd for $C_{57}H_{111}O_{12}Si_6ThNa$: C 48.48, H 7.92. Found: C 44.23, H 6.87. Low values were observed across multiple runs and suggests incomplete combustion which has been problematic for high silicon-containing actinide species.[1,2,5,9,13] The calculated C:H ratio was $C_{57}H_{105.5}$. The combustion values are suggestive of bulk formulation as [Na(crown)(THF)$_x$][Cp″$_3$Th] but crystallization repeatedly afforded single crystals of [Na(crown)$_2$][Cp″$_3$Th].

Synthesis of [Rb(crypt)][Cp″$_3$Th]. As above, Cp″$_3$Th (50 mg, 0.058 mmol) and crypt (22 mg, 0.058 mmol) were reacted with a Rb (14 mg, 0.16 mmol) smear at −35 °C. Dark blue/red dichroic crystals were grown overnight from Et_2O/hexane at −35 °C (47 mg, 61%). ^{1}H NMR (THF-d_8): δ 4.84 (s, 9H, $C_5H_3R_2$), 3.54 (s, 14H, OCH_2CH_2O), 3.49 (m, 13H, crypt), 2.51 (m, 15H, crypt), 0.44 ppm (s, 53H, Si*Me*$_3$). ^{13}C (THF-d_8): δ 120.4 ($C_5H_3R_2$), 114.9 ($C_5H_3R_2$), 111.9 ($C_5H_3R_2$), 71.3 (crypt), 68.4 (crypt), 54.9 (crypt), 2.2 ppm (Si*Me*$_3$). ^{29}Si NMR (THF-d_8): δ −15.69 ppm (*Si*Me$_3$). UV-visible (THF): 656 nm (19,000 M^{-1}cm^{-1}). IR: 2944m, 2884m, 2810m,

1352m, 1296m, 1233s, 1171s, 1102s, 1070s, 947s, 909s, 818s, 782m, 742s, 674s cm^{-1}. Anal Calcd for $C_{51}H_{99}N_2O_6Si_6ThRb$: C 46.32, H 7.55, N 2.12. Found: C 43.80, H 7.20, N 2.53. Low C values were observed across multiple runs and suggests incomplete combustion or carbide formation which has been problematic for high silicon-containing actinide species.[1,2,5,9,13] The calculated C:H:N ratio of $C_{51}H_{99.9}N_{2.5}$ is close to the expected value.

Synthesis of [Cs(crypt)][Cp″$_3$Th]. As above, Cp″$_3$Th (52 mg, 0.060 mmol) and crypt (22 mg, 0.058 mmol) were reacted with a Cs (10 mg, 0.075 mmol) smear at −35 °C. Dark blue/red dichroic crystals were grown overnight from Et_2O/hexane at −35 °C (43 mg, 54%). ^{1}H NMR (THF-d_8): δ 5.51 (s, 9H, $C_5H_3R_2$), 3.57 (m, 18H, OCH_2CH_2O) (overlapping with THF), 3.50 (m, 7H, crypt), 2.54 (m, 9H, crypt), 0.87 ppm (s, 37H, SiMe_3). ^{13}C (THF-d_8): δ 121.9 ($C_5H_3R_2$), 117.0 ($C_5H_3R_2$), 115.2 ($C_5H_3R_2$), 71.4 (OCH_2CH_2O), 68.3 (crypt), 54.6 (crypt), 1.5 ppm (SiMe_3). ^{29}Si NMR (THF-d_8): δ −14.88 ppm (*Si*Me$_3$). ^{133}Cs NMR (THF-d_8): δ 20 ppm (br, $v_{1/2}$ = 3600 Hz). UV-visible (THF): 658 nm (14,000 M^{-1}cm^{-1}). IR: 2944m, 2884m, 2809m, 1349m, 1295m, 1233s, 1171s, 1098s, 1064s, 942m, 909s, 817s, 782m, 742s, 674s cm^{-1}. Anal Calcd for $C_{51}H_{99}N_2O_6Si_6ThCs$: C 44.72, H 7.28, N 2.05. Found: C 40.81, H 6.59, N 1.57. Low values were observed across multiple runs and suggests incomplete combustion which has been problematic for high silicon-containing actinide species.[1,2,5,9,13] The calculated C:H:N ratio of $C_{51}H_{98.1}N_{1.7}$ is close to the expected value.

Synthesis of [K(crown)(THF)$_2$][Cp″$_3$Th] from Cp″$_3$ThCl. Cp″$_3$ThCl (77 mg, 0.093 mmol) and 18-crown-6 (24 mg, 0.091 mmol) were dissolved in THF (3 mL). Pre-cooled KC_8 (31 mg, 0.229 mmol) was added and the reaction was stirred for approximately 5 minutes. The initially colorless solution turned bright blue, followed by the change to inky blue/green. Black solids were removed via centrifugation and the solution was dried under vacuum. The product was extracted

in Et_2O, filtered, and dried. The solids were washed with hexane to remove Cp''_3Th and dried. Dark blue crystals of $[K(crown)(THF)_2][Cp''_3Th]$[9] were grown overnight from Et_2O/hexane at −35 °C (58 mg, 50%).

Reaction of Cp''_3ThBr with Ba. Cp''_3ThBr (47 mg, 0.054 mmol) was dissolved in THF (3 mL). Freshly shaved Ba powder (excess) was added and the solution was stirred vigorously. After approximately four hours of stirring, the solution began to turn blue. No further color changes were observed after an additional 5 hours of stirring. Cp''_3Th was identified by UV-visible spectroscopy.[5,8]

Reaction of Cp''_3Th with Ba. Cp''_3Th (26 mg, 0.030 mmol) was dissolved in THF (3 mL). Freshly shaved Ba powder (excess) was added and the solution was placed in the freezer overnight. The solution was stirred vigorously for approximately two hours of stirring at which point the solution began to turn dark blue/green. The solution was dried and the solids were washed with hexane to remove Cp''_3Th. The remaining solids were extracted into THF and the presence of $[Cp''_3Th]^{1-}$ was confirmed by UV-visible spectroscopy.

Reaction of Cp''_3ThBr with Ba and crown. Cp''_3ThBr (42 mg, 0.048 mmol) and crown (13 mg, 0.049 mmol) were dissolved in THF (3 mL). Freshly shaved Ba powder (excess) was added and the solution was stirred vigorously. After approximately 10 minutes of stirring, the solution began to turn blue. The solution was placed in the freezer overnight and maintained the dark blue color of Cp''_3Th. The solution was stirred again at which point a dark blue/green color developed. The solution was stirred for one hour and the presence of $[Cp''_3Th]^{1-}$ was confirmed by UV-Visible spectroscopy.

Synthesis of [K(crown)][Cp″]. In a J-Young NMR tube, 18-crown-6 (8.7 mg, 0.033 mmol) was added to a solution of KCp″ (8.2 mg, 0.033 mmol) in THF-d_8 (1 mL). The solution

was mixed by inversion multiple times before the spectrum was collected. ^{1}H spectroscopy showed quantitative conversion to [K(crown)][Cp″]. ^{1}H NMR (THF-d_8): δ 6.10 (s, 1H, $C_5H_3(SiMe_3)_2$), 5.95 (m, 2H, $C_5H_3(SiMe_3)_2$), 3.51 (s, 24H, O–CH_2CH_2–O), 0.06 ppm (s, 18H, SiMe_3). Cf. KCp″ ^{1}H NMR (THF-d_8): δ 6.09 (s, 1H, $C_5H_3(SiMe_3)_2$), 6.00 (m, 2H, $C_5H_3(SiMe_3)_2$), 0.07 ppm (s, 18H, SiMe_3).

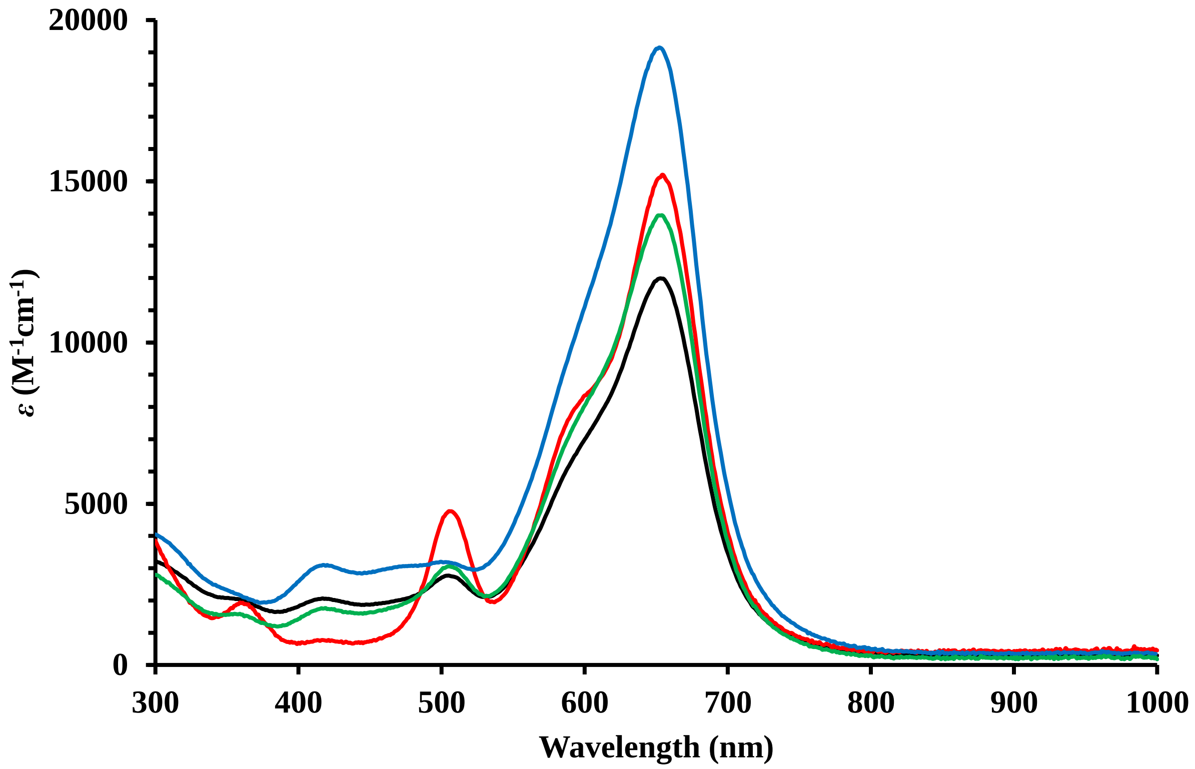

Figure S1: UV-Visible spectra of [Li(crypt)][Cp″$_3$Th] (black), [Na(crown)$_2$][Cp″$_3$Th] (red), [Rb(crypt)][Cp″$_3$Th] (blue), and [Cs(crypt)][Cp″$_3$Th] (green) in THF. The peak at 510 nm is due to Cp″$_3$Th.[5,8]

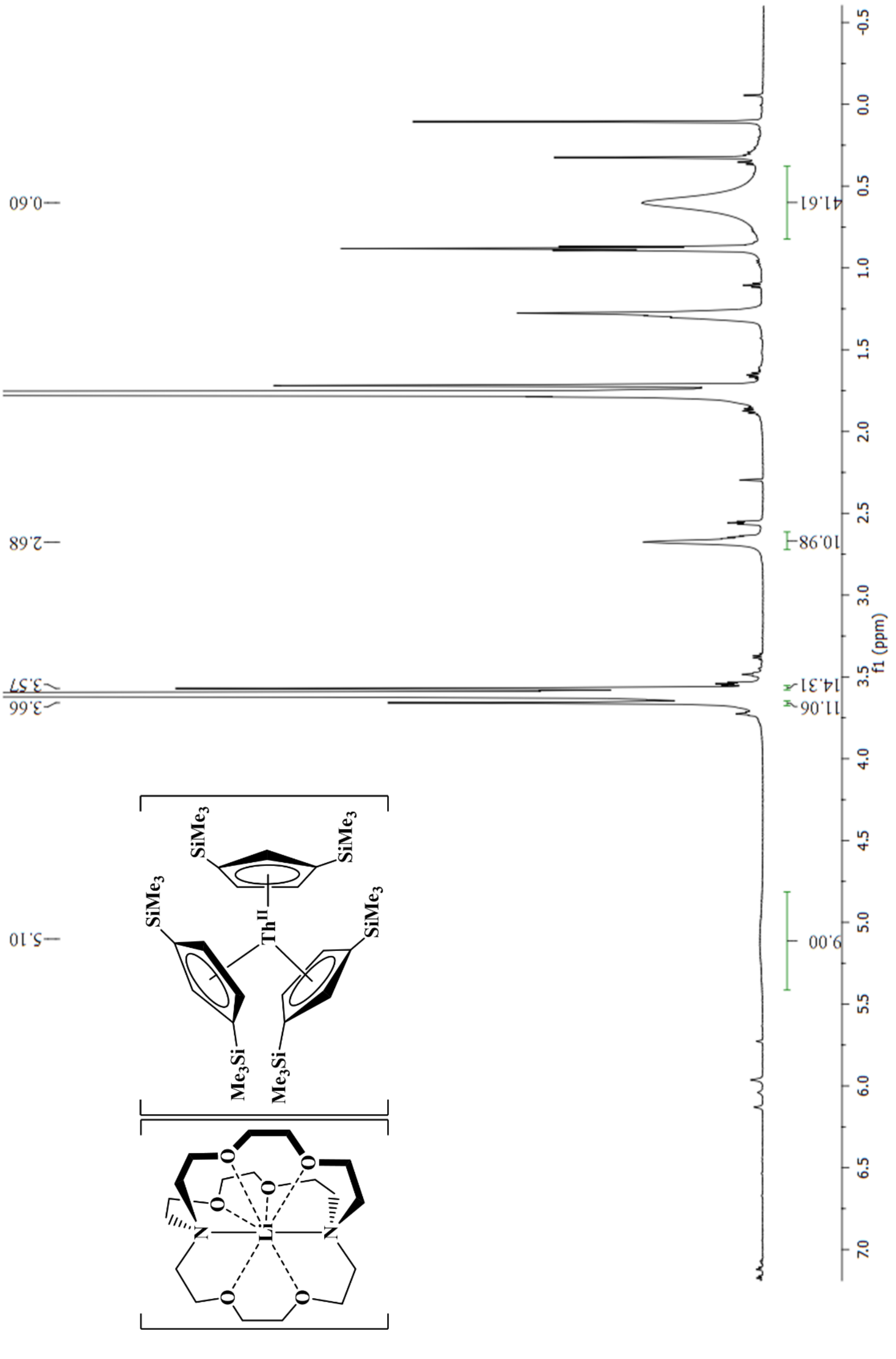

Figure S2: ^{1}H NMR spectrum of [Li(crypt)][Cp″$_3$Th] in THF-d_8.

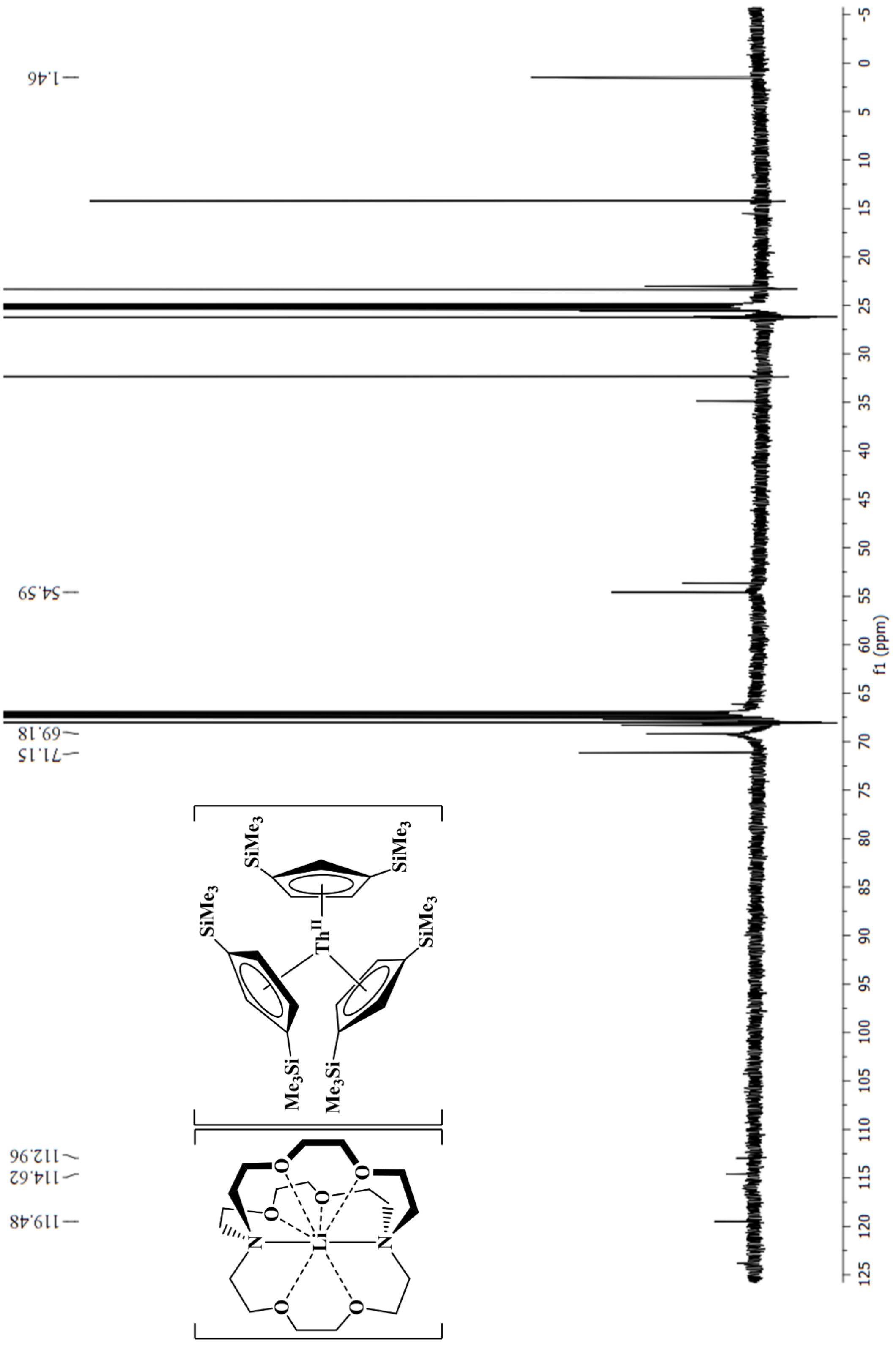

Figure S3: ^{13}C NMR spectrum of [Li(crypt)][Cp″$_3$Th] in THF-d_8.

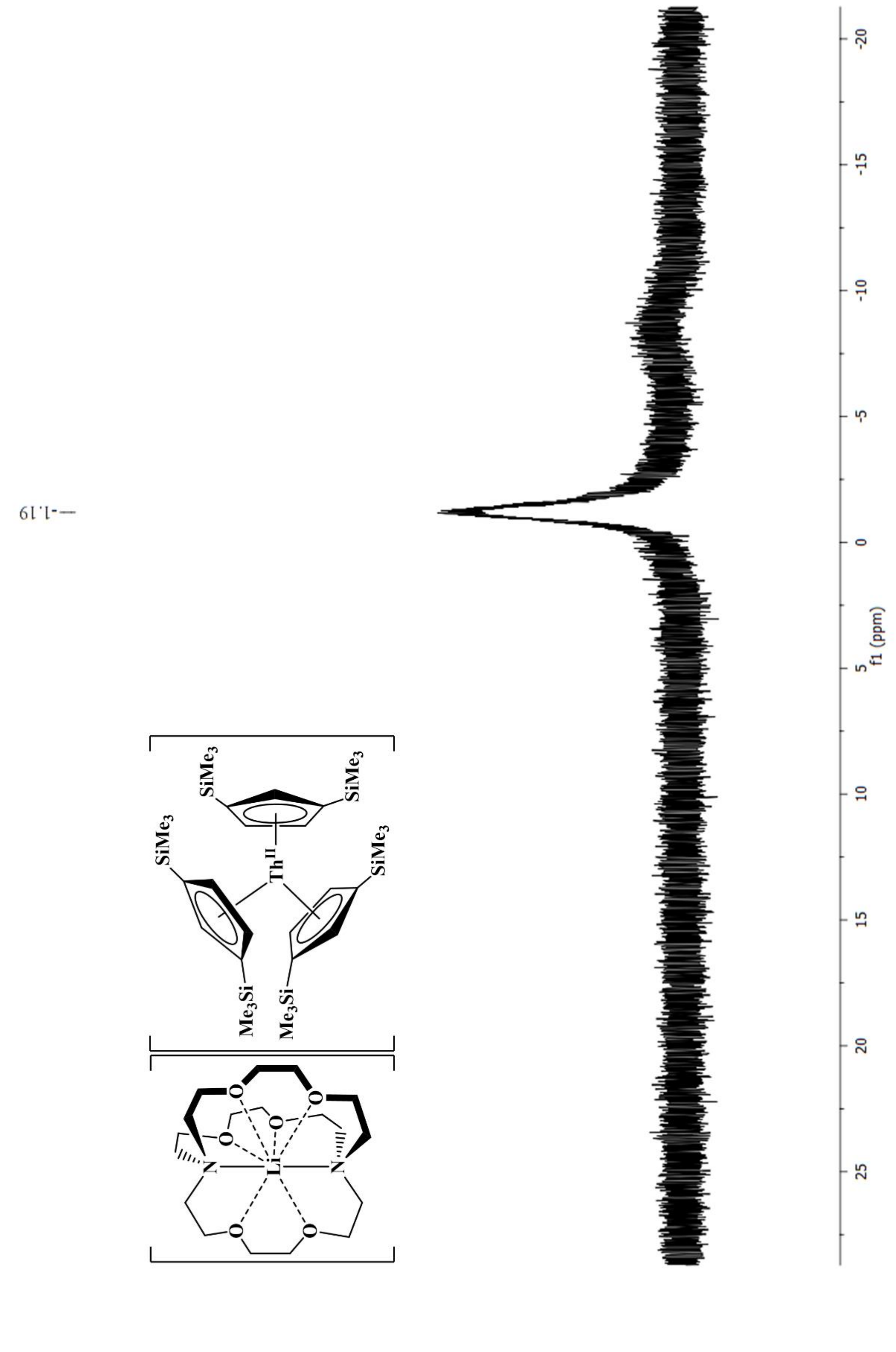

Figure S4: ^{7}Li NMR spectrum of [Li(crypt)][Cp″$_3$Th] in THF-d_8.

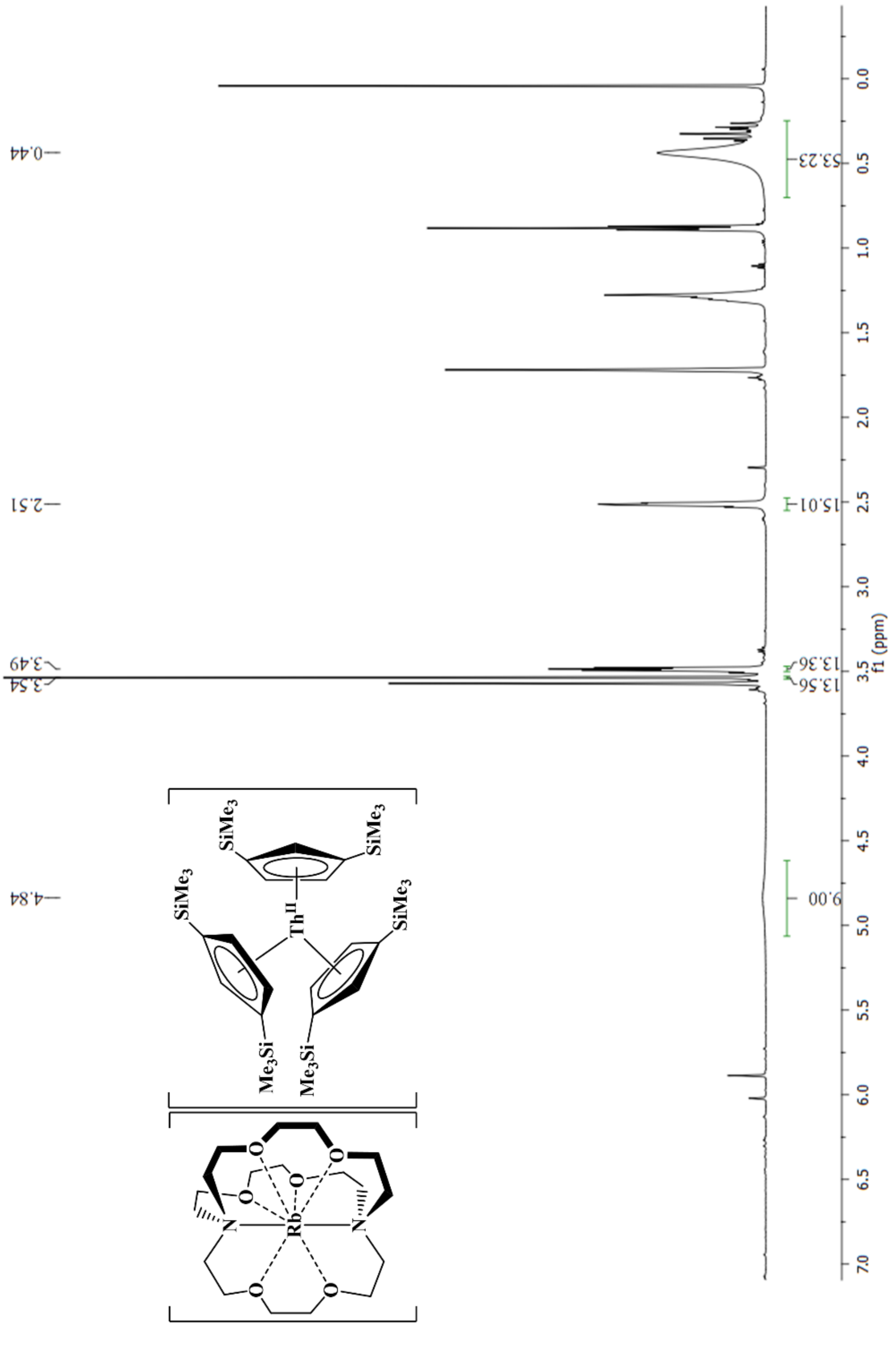

Figure S5: ^{1}H NMR spectrum of [Rb(crypt)][Cp″$_3$Th] in THF-d_8.

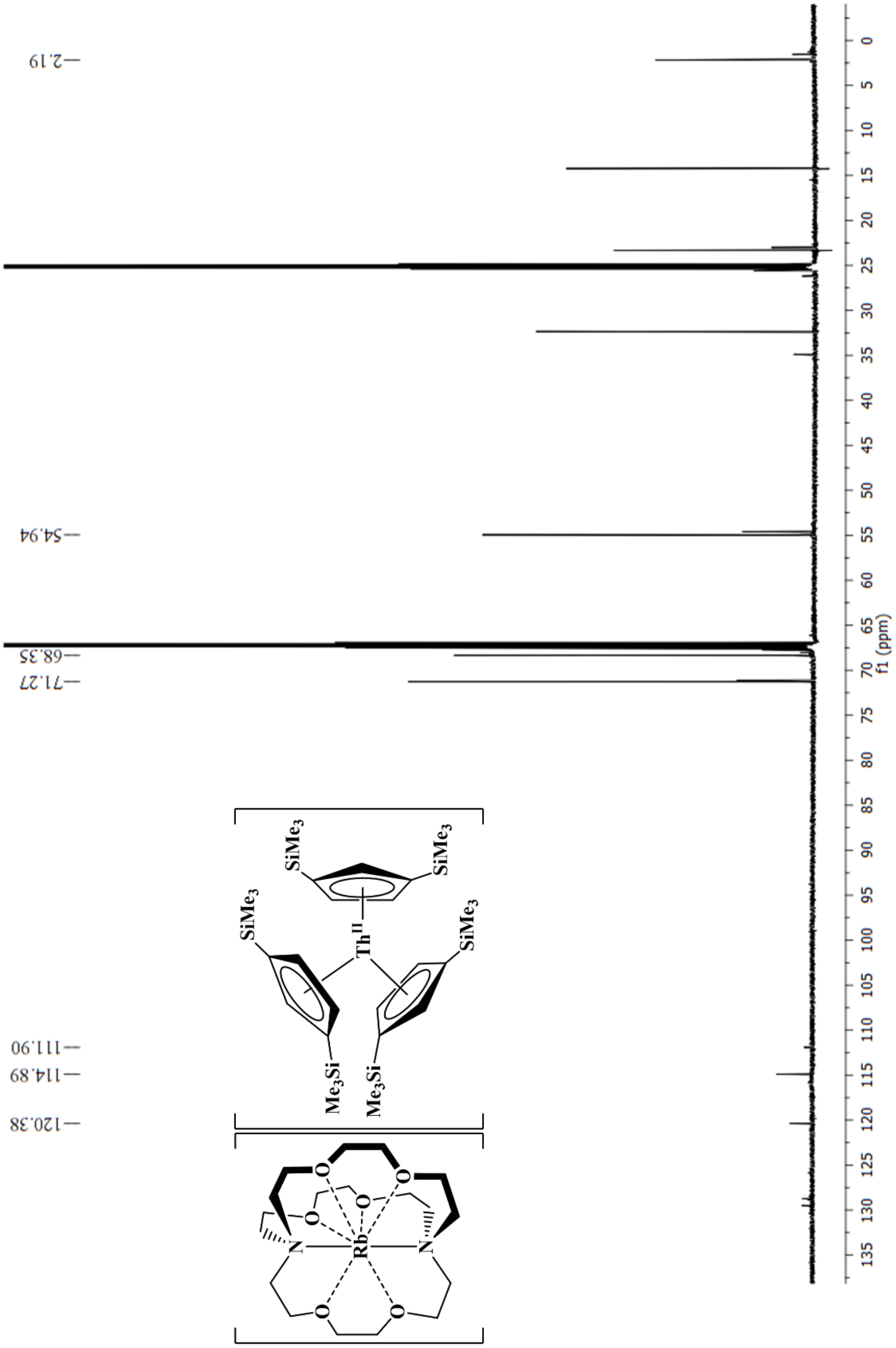

Figure S6: ^{13}C NMR spectrum of [Rb(crypt)][Cp″$_3$Th] in THF-d_8.

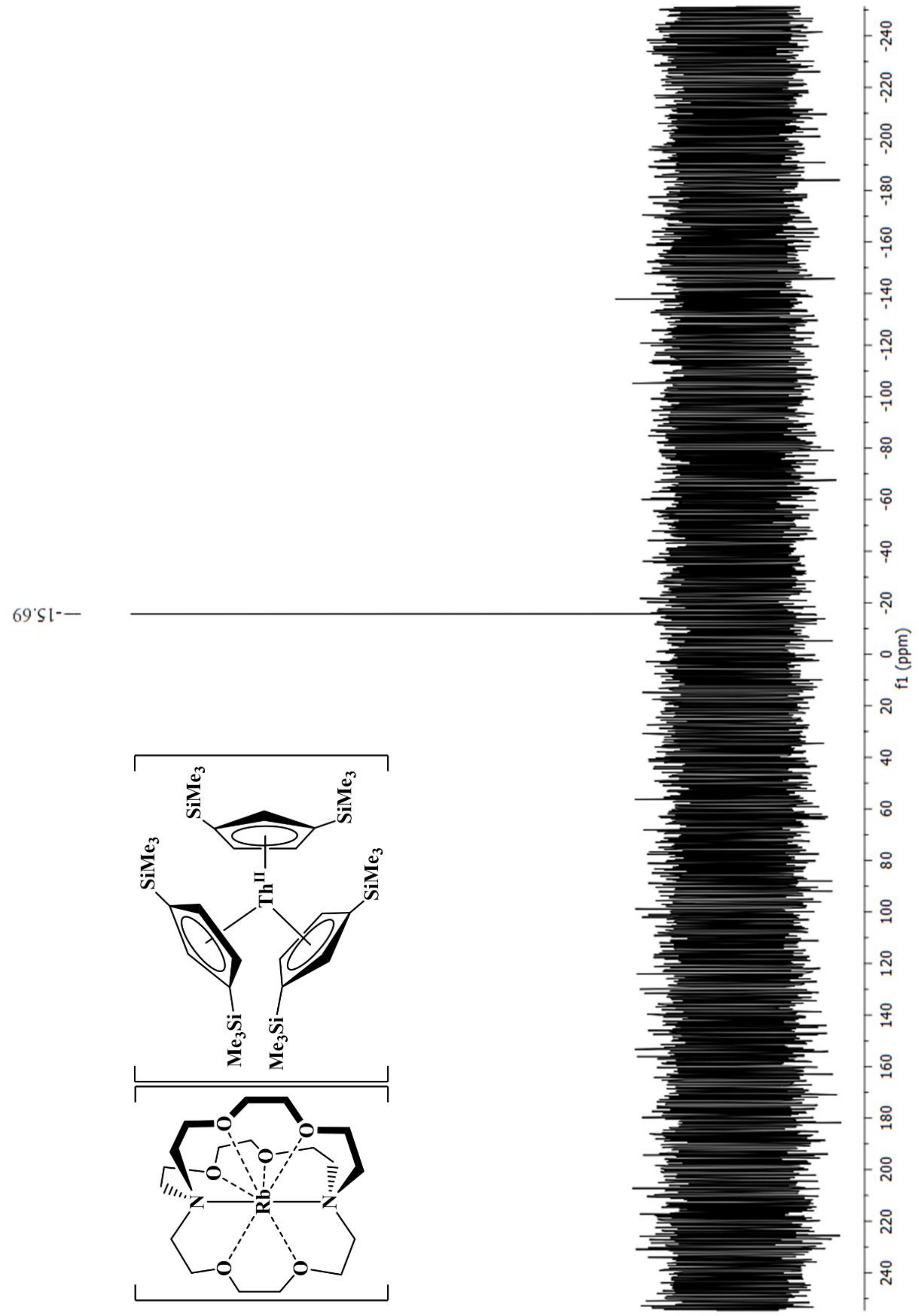

Figure S7: ^{29}Si NMR spectrum of [Rb(crypt)][Cp″$_3$Th] in THF-d_8.

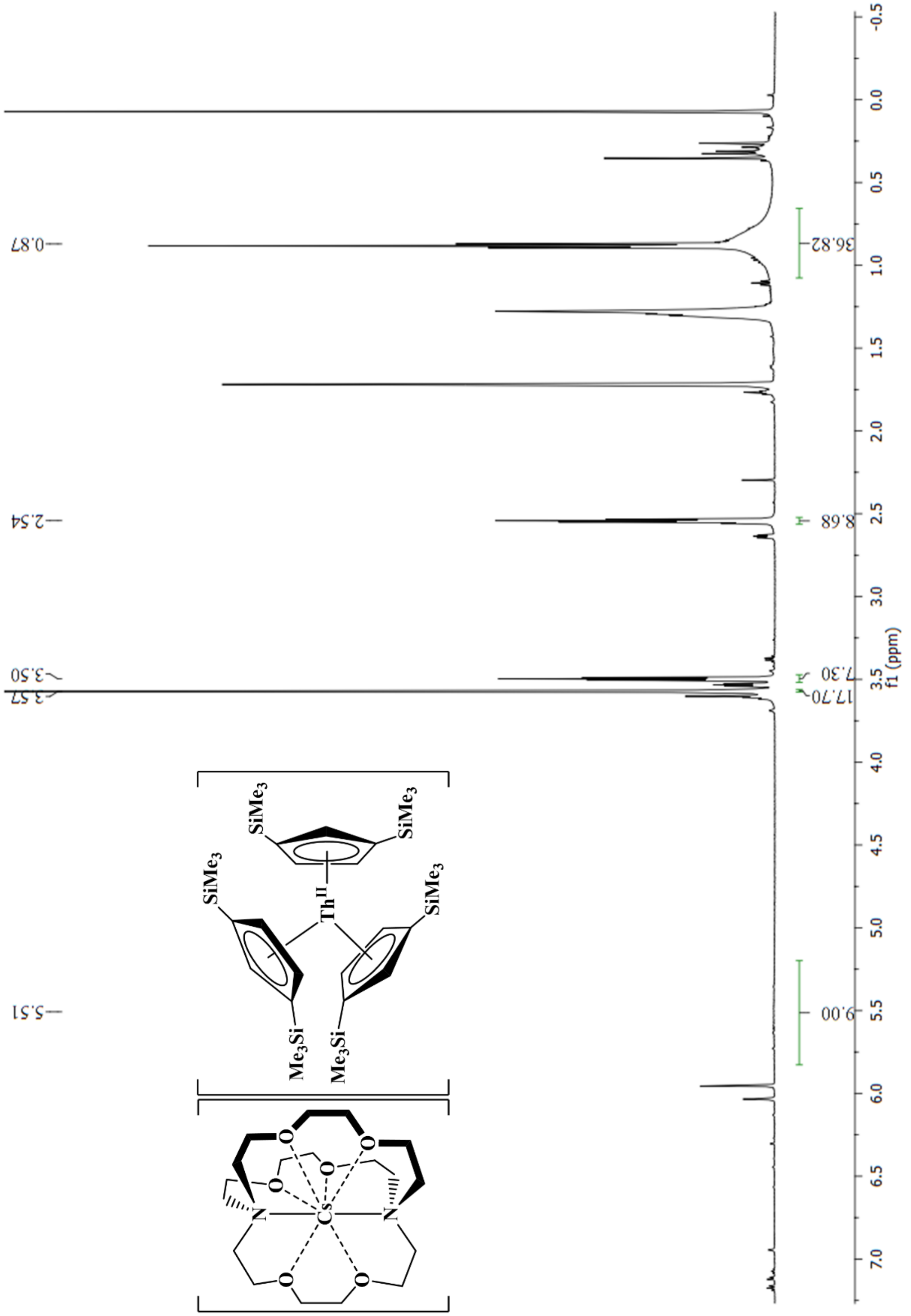

Figure S8: ^{1}H NMR spectrum of [Cs(crypt)][Cp″$_3$Th] in THF-d_8.

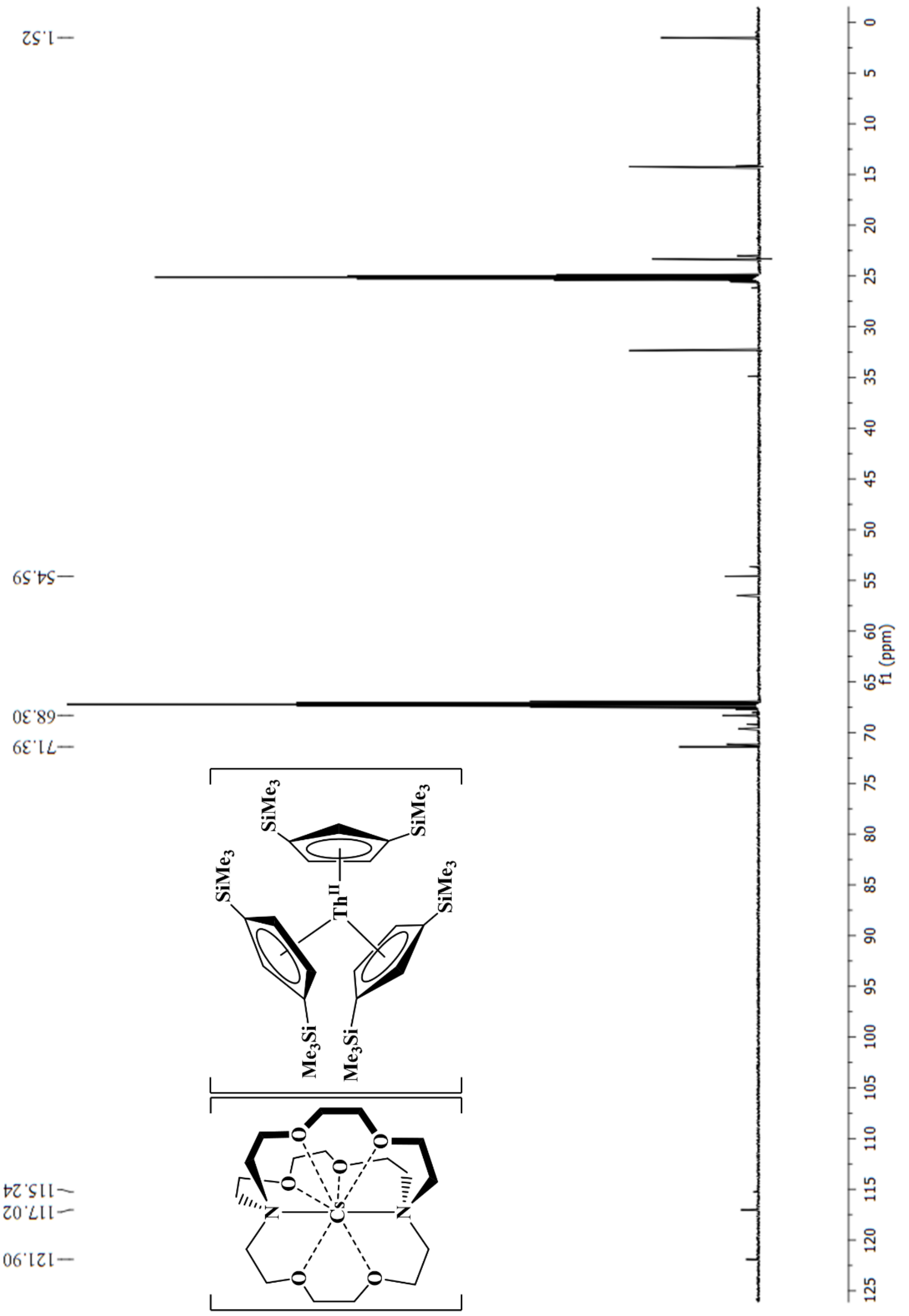

Figure S9: ^{13}C NMR spectrum of [Cs(crypt)][Cp″$_3$Th] in THF-d_8.

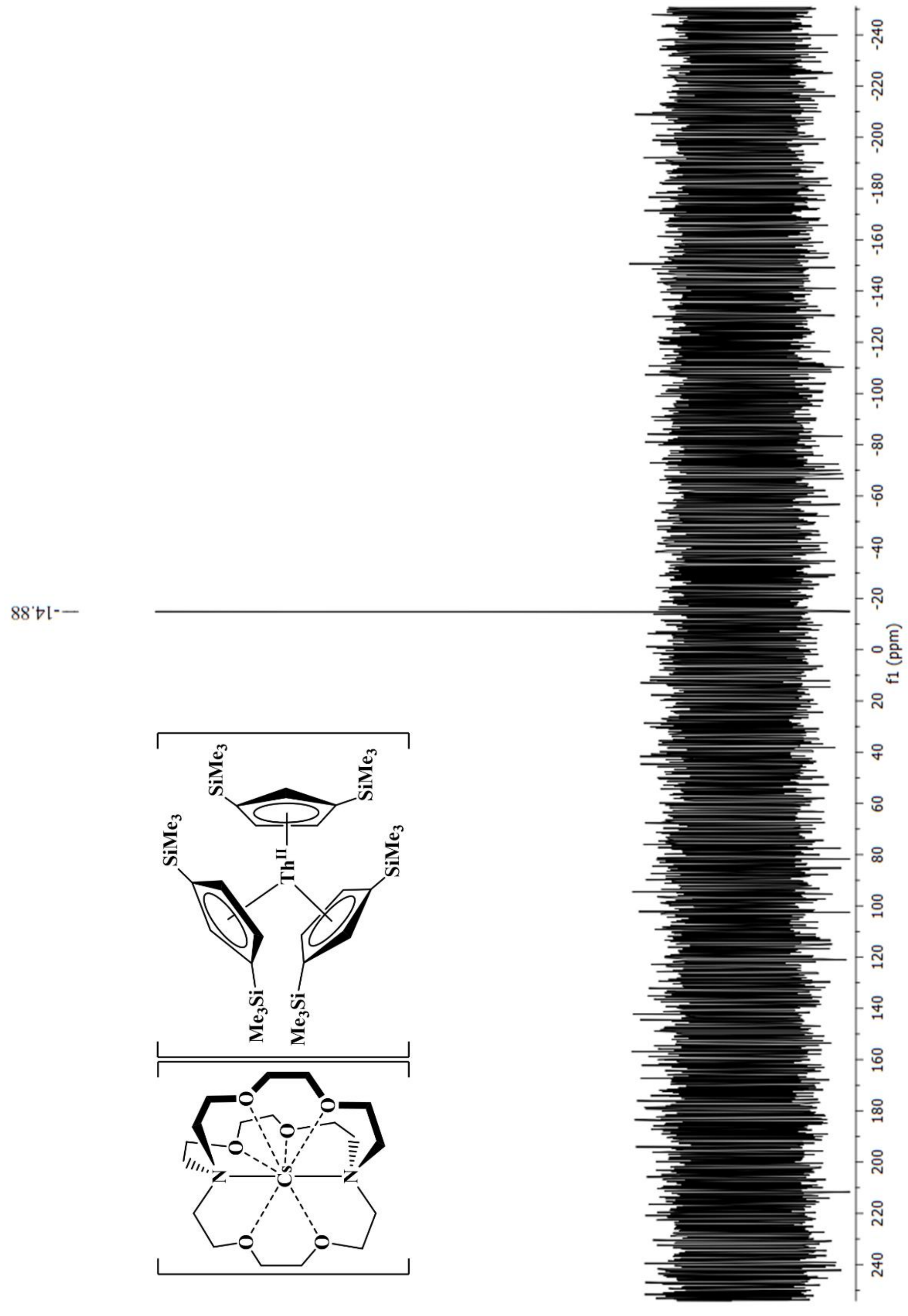

Figure S10: ^{29}Si NMR spectrum of [Cs(crypt)][Cp″$_3$Th] in THF-d_8.

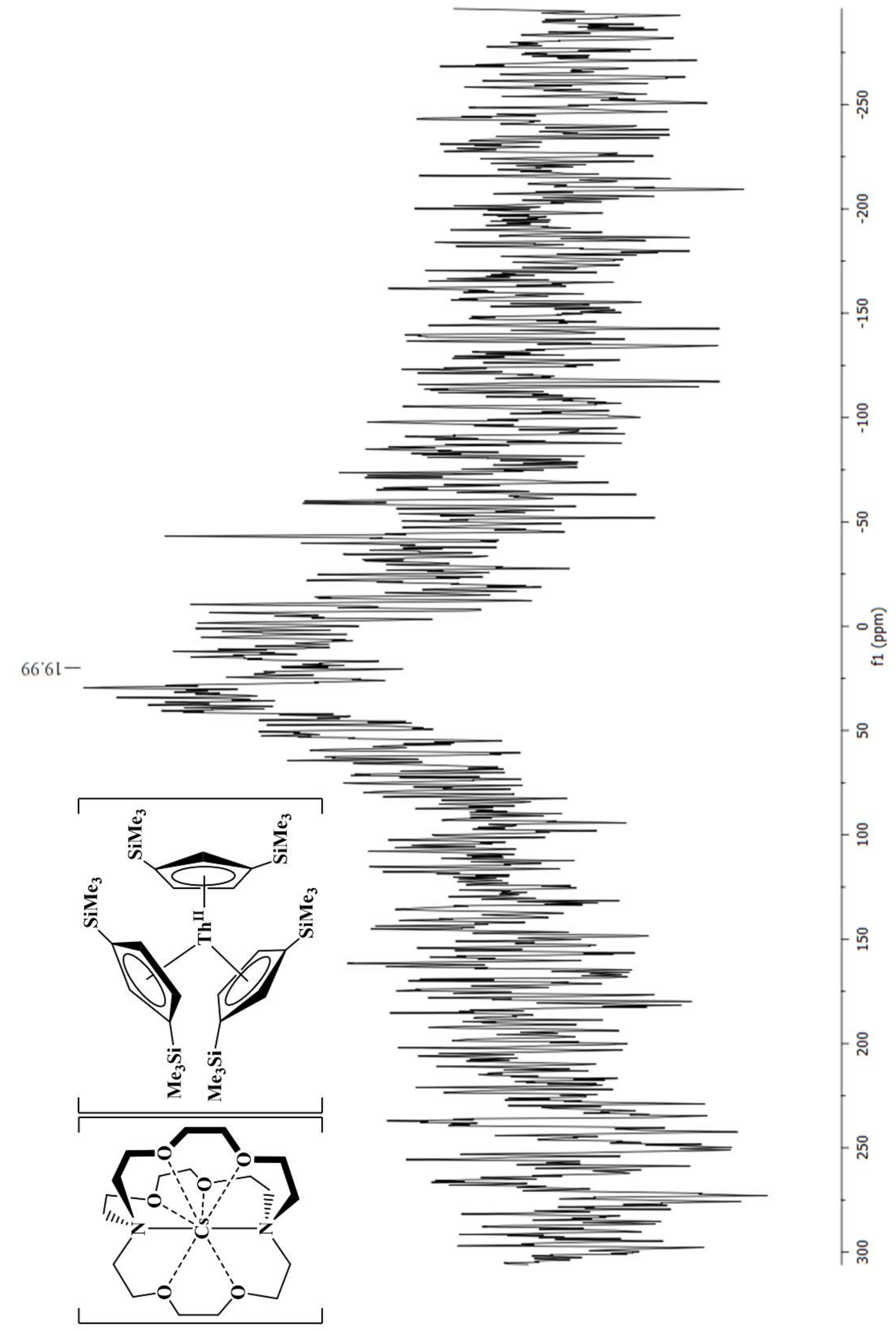

Figure S11: ^{139}Cs NMR spectrum of [Cs(crypt)][Cp″$_3$Th] in THF-d_8.

Table S1: Reduction potentials of tris(cyclopentadienyl) thorium complexes with 200 mM [^{n}Bu$_4$N][PF$_6$] supporting electrolyte

	Th(IV)/Th(III)			Th(III)/Th(II)			
	E_{PC} (V)	E_{PA} (V)	$E_{1/2}$ (V)	E_{PC} (V)	E_{PA} (V)	$E_{1/2}$ (V)	ΔE_{pp} Fc (V)
Cp″$_3$Th^{IV}Br	−2.99	−2.73	−2.86				0.17
Cp′$_3$Th^{IV}Cl	−3.39	−2.70	−3.04				0.14
Cp$^{tet}_3$Th^{IV}Br	−3.29	−3.20	−3.24				0.13
Cp″$_3$Th^{III}				−2.94	−2.73	−2.84	0.18
Cp$^{tet}_3$Th^{III}				−3.28	−3.22	−3.25	0.15
[K(crown)(THF)$_2$][Cp″$_3$Th^{II}]				−2.89	−2.73	−2.81	0.13
[K(crypt)][Cp″$_3$Th^{II}]				−2.88	−2.75	−2.82	0.06

Electrochemical Data

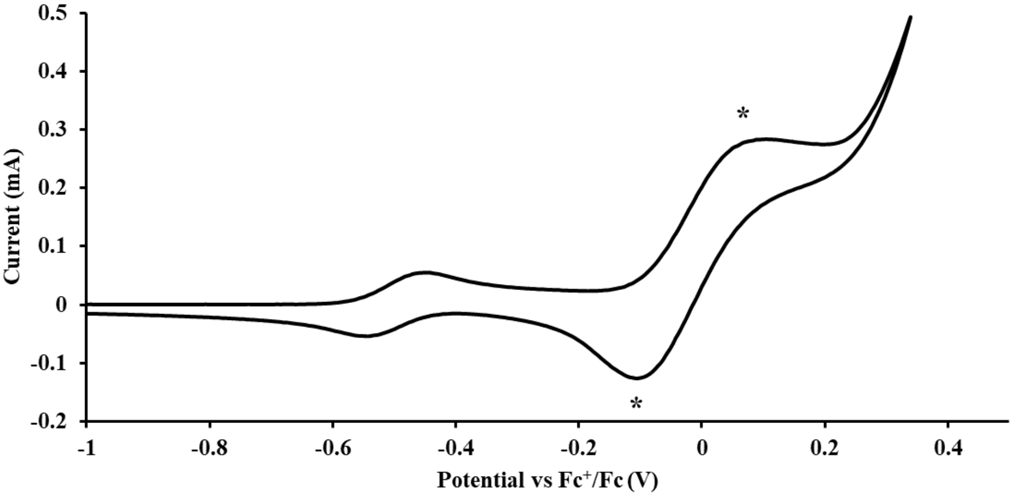

Figure S12: Voltammogram of $(C_5Me_5)_2Fe$ and $(C_5H_5)_2Fe$ (marked with asterisk) in the experimental cell at v = 200 mV/s in 100 mM $TBABPh_4$ / THF. Fc* has a potential of −0.495 V vs Fc^+/Fc under these conditions. ΔE_{pp} (Fc) = 0.20 V, ΔE_{pp} (Fc*) = 0.10 V.

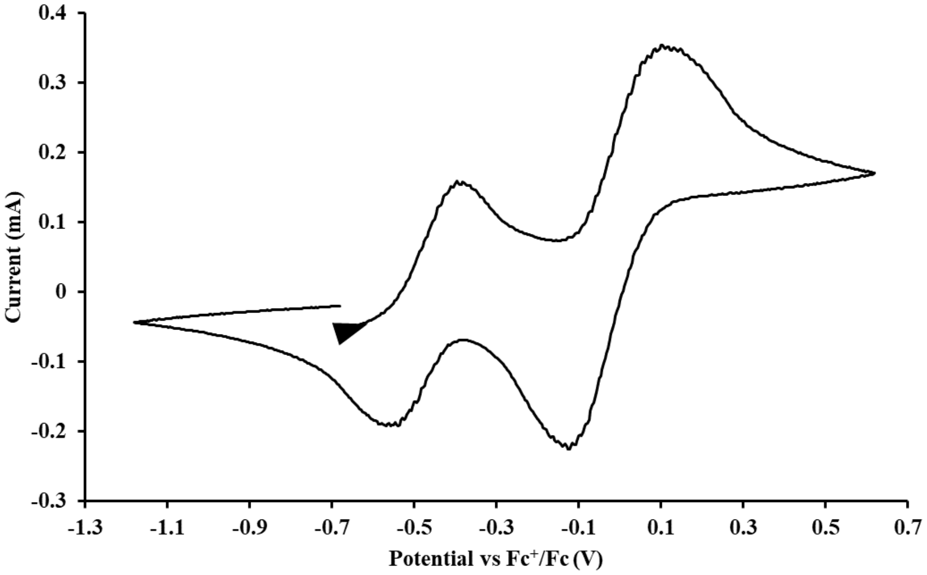

Figure S13: Voltammogram of $(C_5Me_5)_2Fe$ and $(C_5H_5)_2Fe$ (marked with asterisk) in the experimental cell at v = 200 mV/s in 200 mM $TBAPF_6$ / THF. Fc* has a potential of −0.47 V vs Fc^+/Fc under these conditions. ΔE_{pp} (Fc) = 0.24 V, ΔE_{pp} (Fc*) = 0.18 V.

Cp″$_3$U

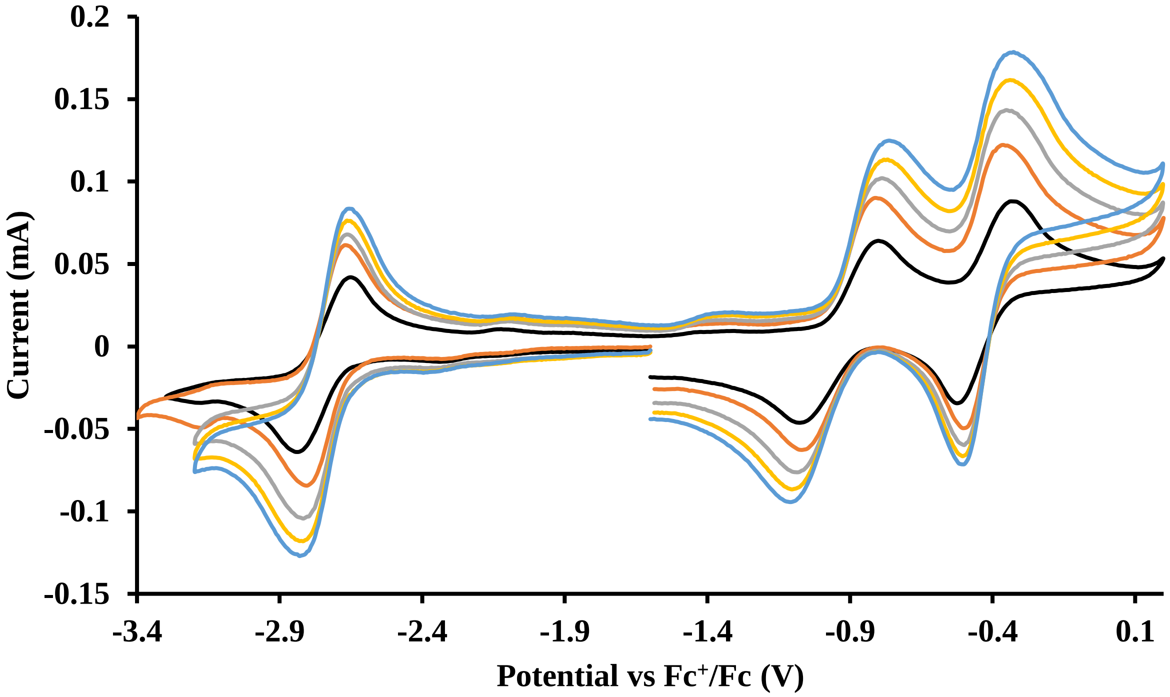

Figure S14: Voltammogram of 4.6 mM Cp″$_3$U at ν = 200 (black), 400 (orange), 600 (grey), 800 (yellow) and 1000 (blue) mV/s in 100 mM TBABPh$_4$ / THF. The event centered at −0.495 V is due to internal standard $(C_5Me_5)_2Fe^{II}$.

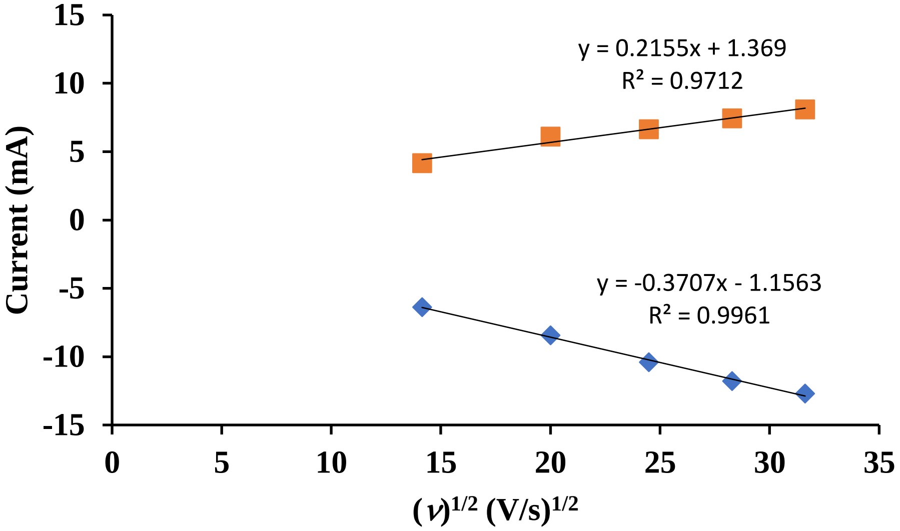

Figure S15: Scan rate dependence plot on the 3/2 couple of Cp″$_3$U.

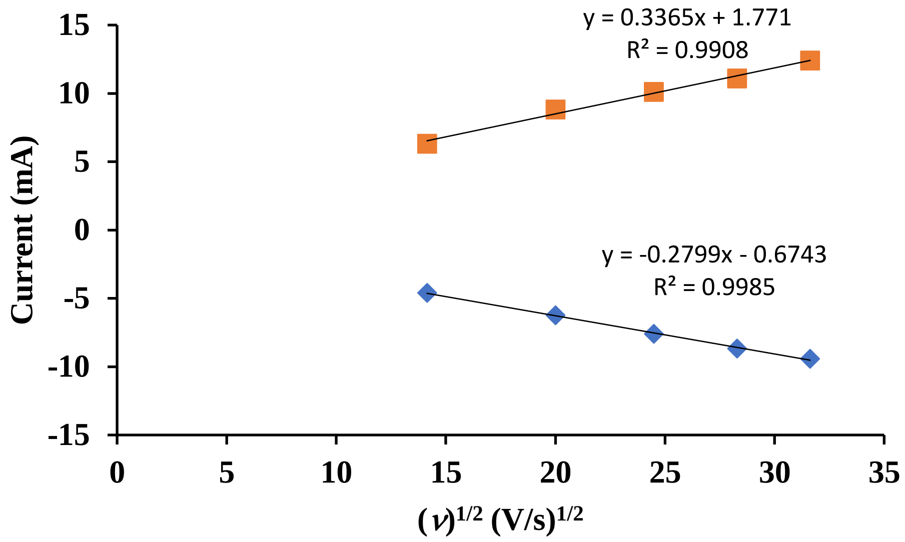

Figure S16: Scan rate dependence plot on the 4/3 couple of Cp''_3U in 100 mM $TBABPh_4$ / THF.

Cp'_3U

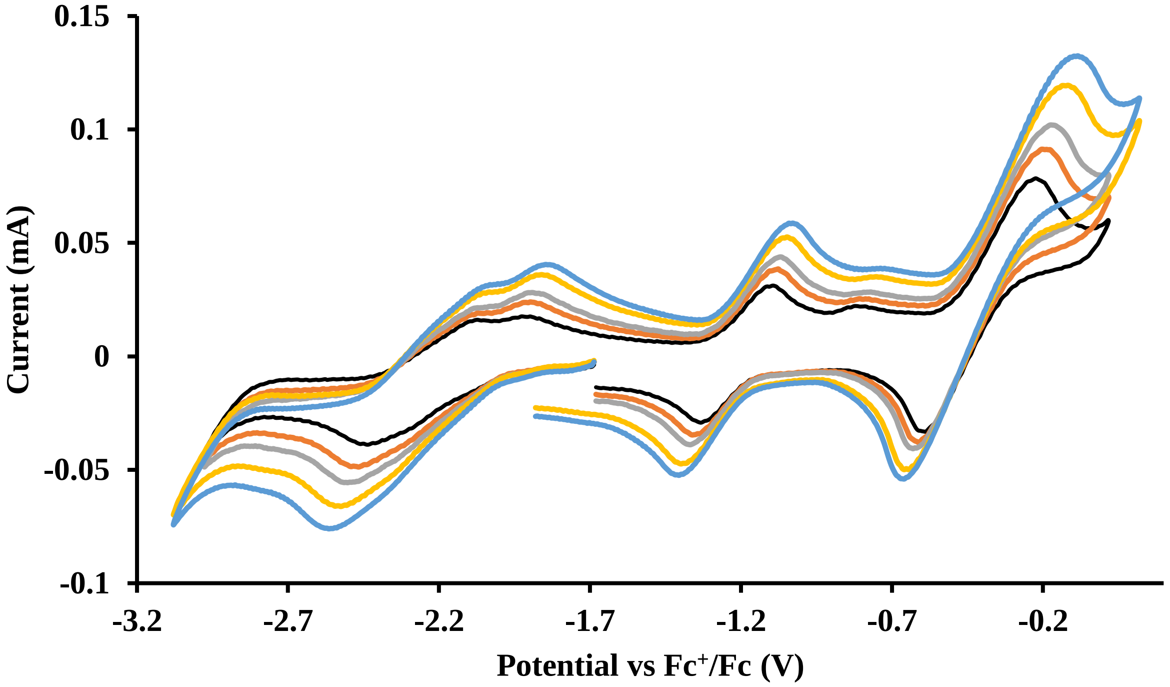

Figure S17: Voltammogram of 11 mM Cp'_3U at ν = 200 (black), 400 (orange), 600 (grey), 800 (yellow) and 1000 (blue) mV/s in 50 mM $TBABPh_4$ / THF. The event centered at −0.495 V is due to internal standard $(C_5Me_5)_2Fe^{II}$.

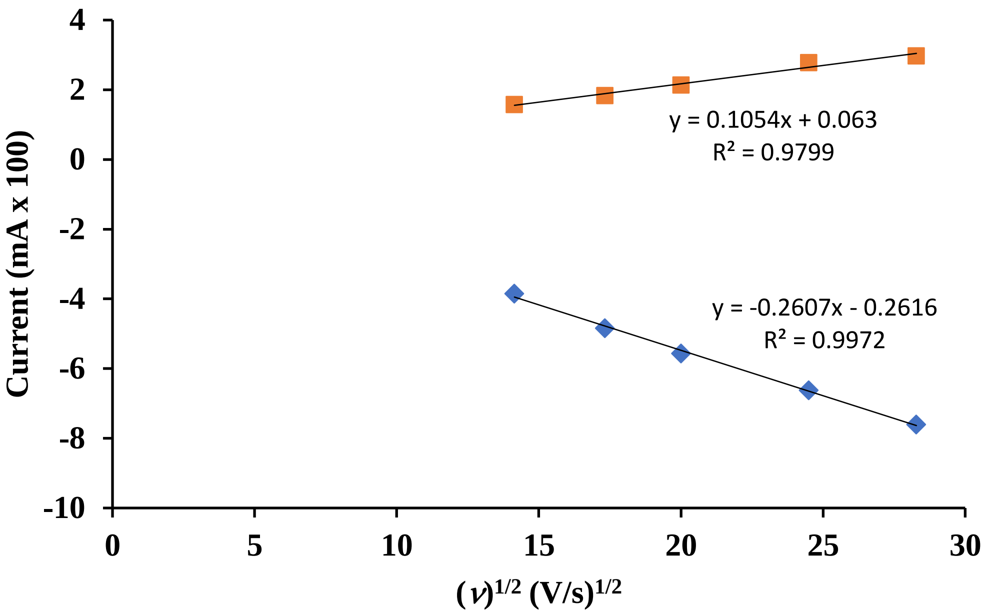

Figure S18: Scan rate dependence plot on the 3/2 couple of Cp′$_3$U in 50 mM TBABPh$_4$ / THF.

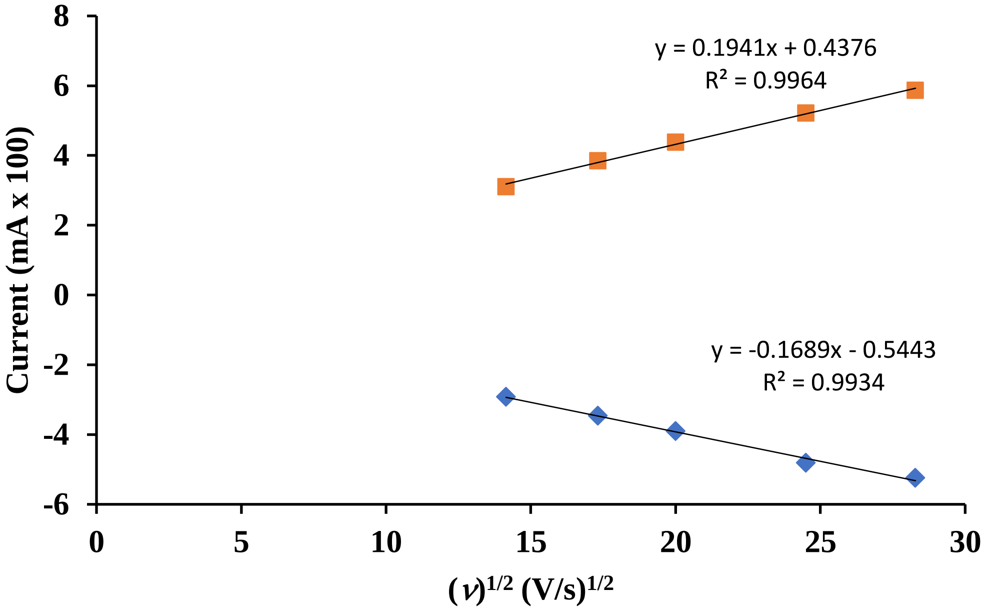

Figure S19: Scan rate dependence plot on the 3/2 couple of Cp′$_3$U in 50 mM TBABPh$_4$ / THF.

$Cp^{tet}{}_3U$

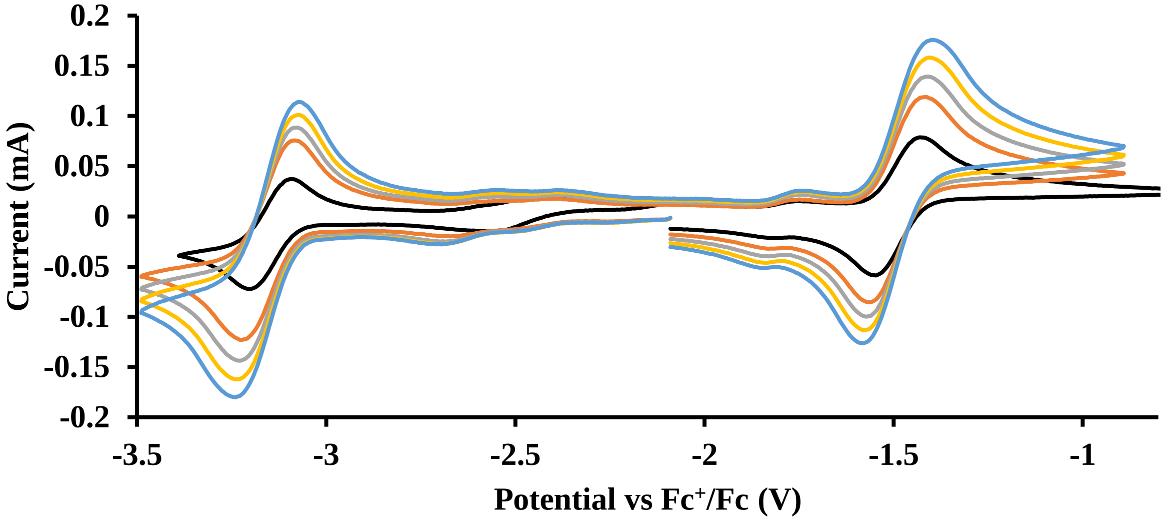

Figure S20: Voltammogram of 7.2 mM $Cp^{tet}{}_3U$ at ν = 200 (black), 400 (orange), 600 (grey), 800 (yellow) and 1000 (blue) mV/s in 100 mM $TBABPh_4$ / THF.

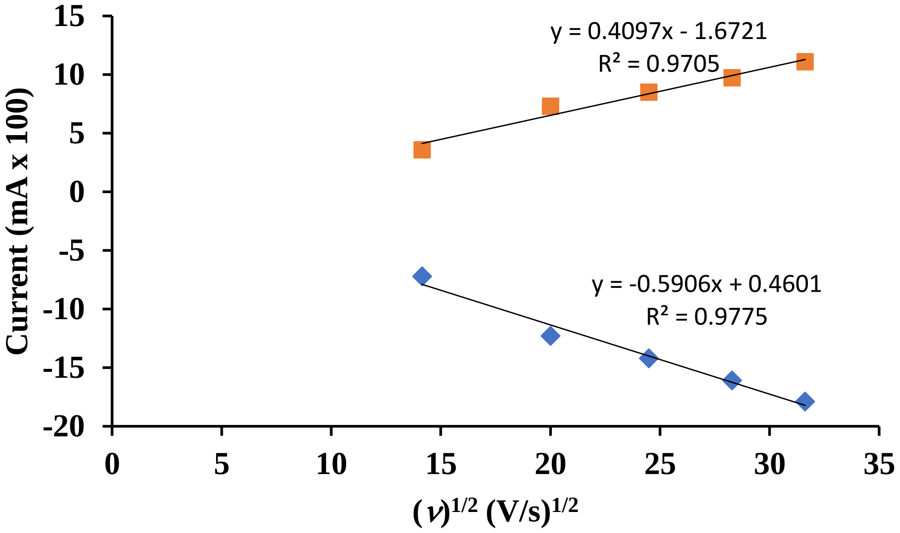

Figure S21: Scan rate dependence plot on the 3/2 couple of $Cp^{tet}{}_3U$ in 100 mM $TBABPh_4$ / THF.

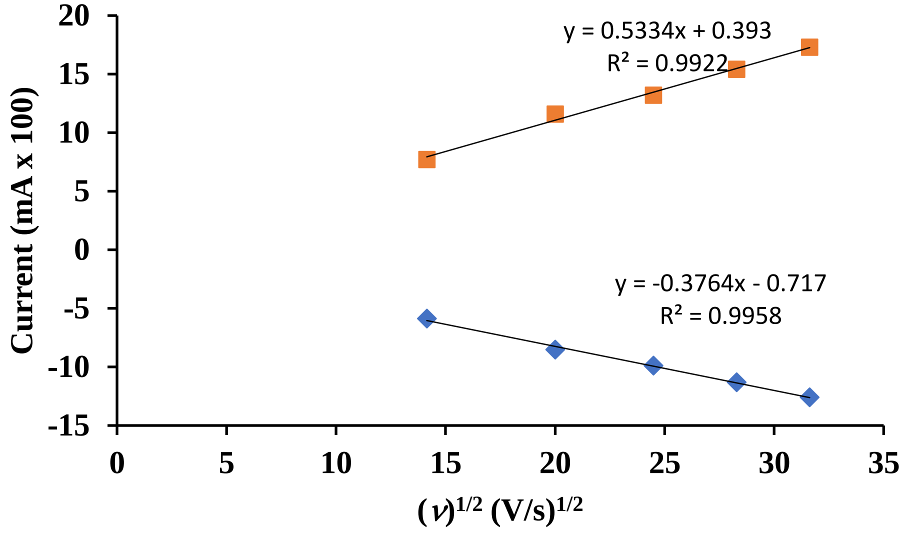

Figure S22: Scan rate dependence plot on the 4/3 couple of $Cp^{tet}{}_3U$ in 100 mM $TBABPh_4$ / THF.

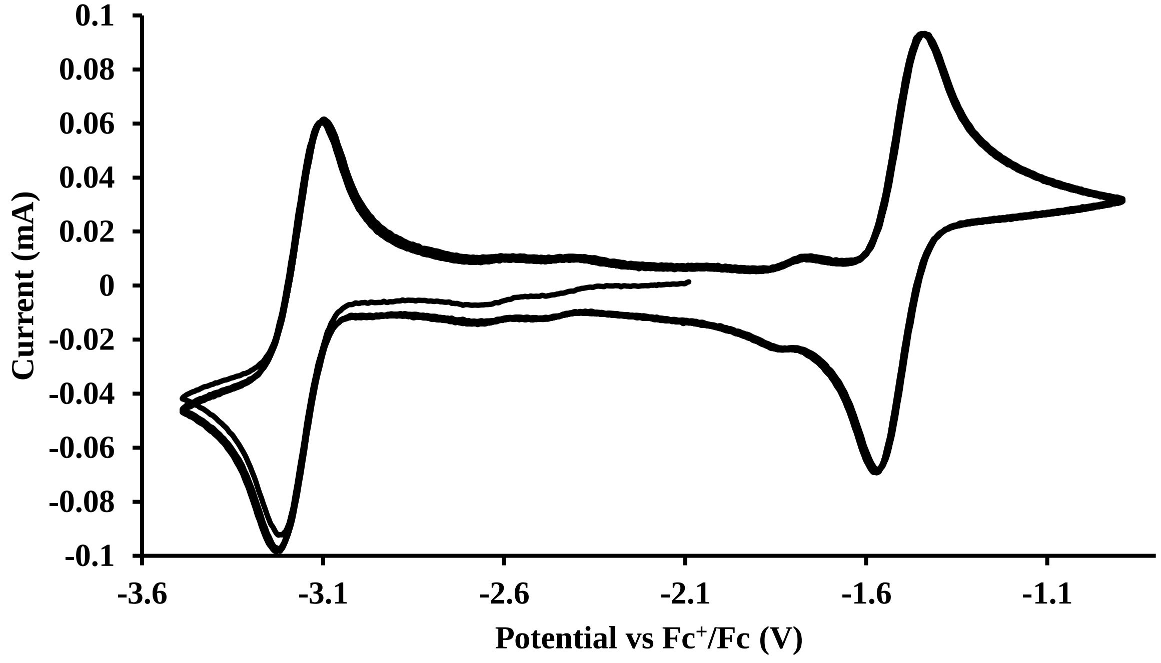

Figure S23: Voltammogram of 7.2 mM $Cp^{tet}{}_3U$ at ν = 200 mV/s over 5 cycles in 100 mM $TBABPh_4$ / THF.

[K(crypt)][Cp′$_3$U]

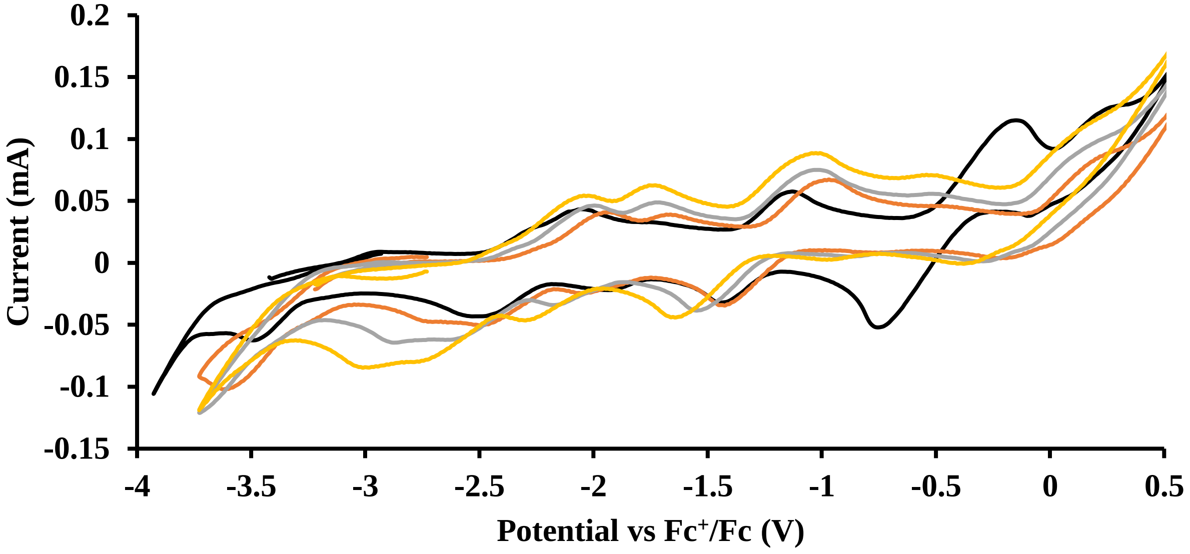

Figure S24: Voltammogram of 7.7 mM [K(crypt)][Cp′$_3$U] at v = 200 (black), 400 (orange), 600 (grey), and 1000 (yellow) mV/s in 100 mM TBABPh$_4$ / THF. The peak centered at −0.495 V is due to internal standard $(C_5Me_5)_2Fe$.

[K(crown)(THF)$_2$][Cp″$_3$U]

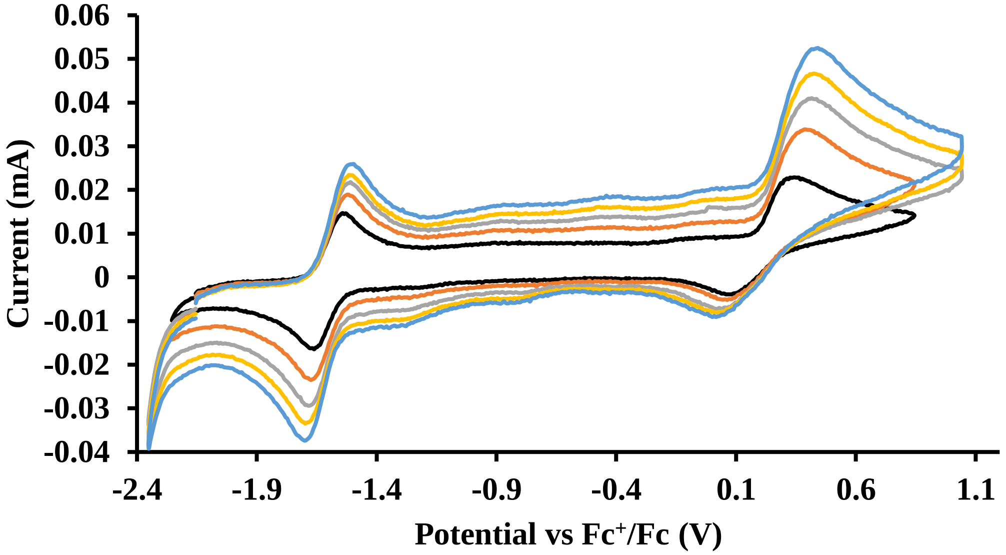

Figure S25: Voltammogram of 3.0 mM [K(crown)(THF)$_2$][Cp″$_3$U] at v = 200 (black), 400 (orange), 600 (grey), 800 (yellow) and 1000 (blue) mV/s in 100 mM TBABPh$_4$ / THF.

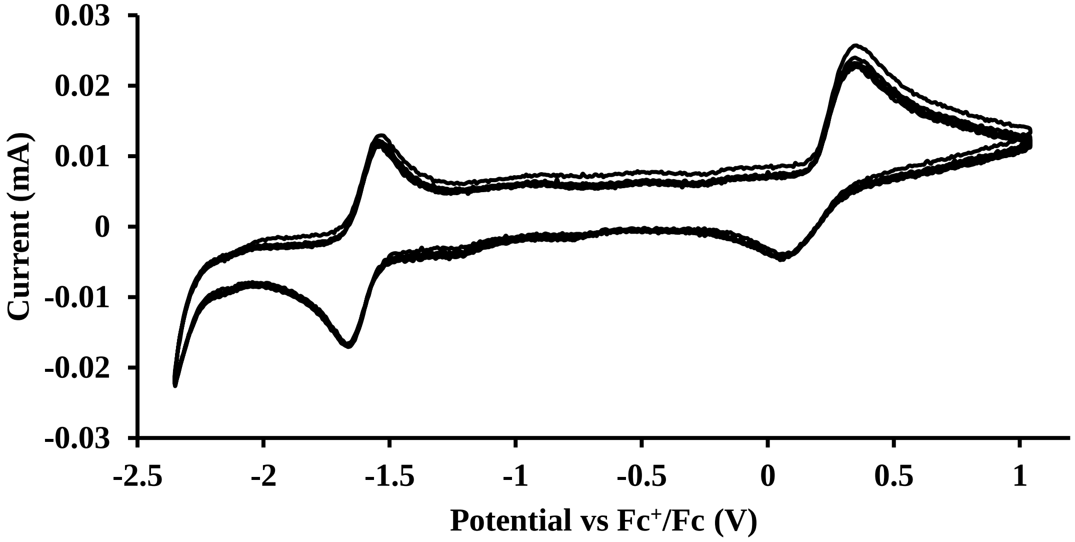

Figure S26: Voltammogram of 3.0 mM [K(crown)(THF)$_2$][Cp″$_3$U] at v = 200 mV/s over 5 cycles in 100 mM TBABPh$_4$ / THF.

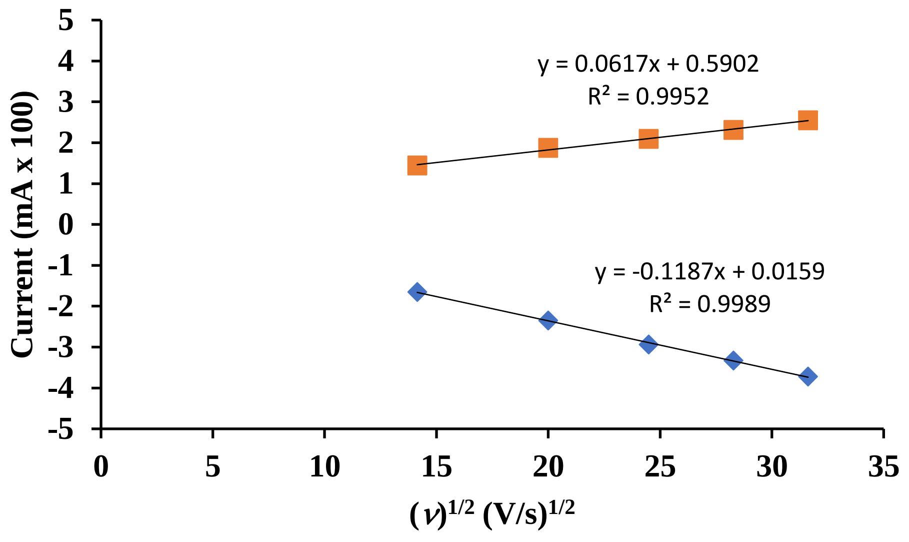

Figure S27: Scan rate dependence plot on the 3/2 couple of [K(crown)(THF)$_2$][Cp″$_3$U] in 100 mM TBABPh$_4$ / THF.

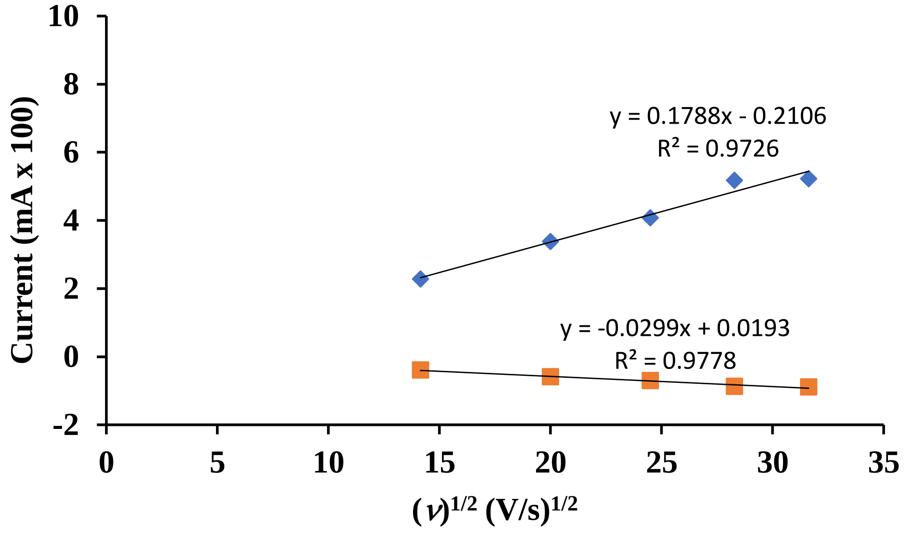

Figure S28: Scan rate dependence plot on the 4/3 couple of [K(crown)(THF)$_2$][Cp″$_3$U] in 100 mM TBABPh$_4$ / THF.

[K(crypt)][$Cp^{tet}{}_3U$]

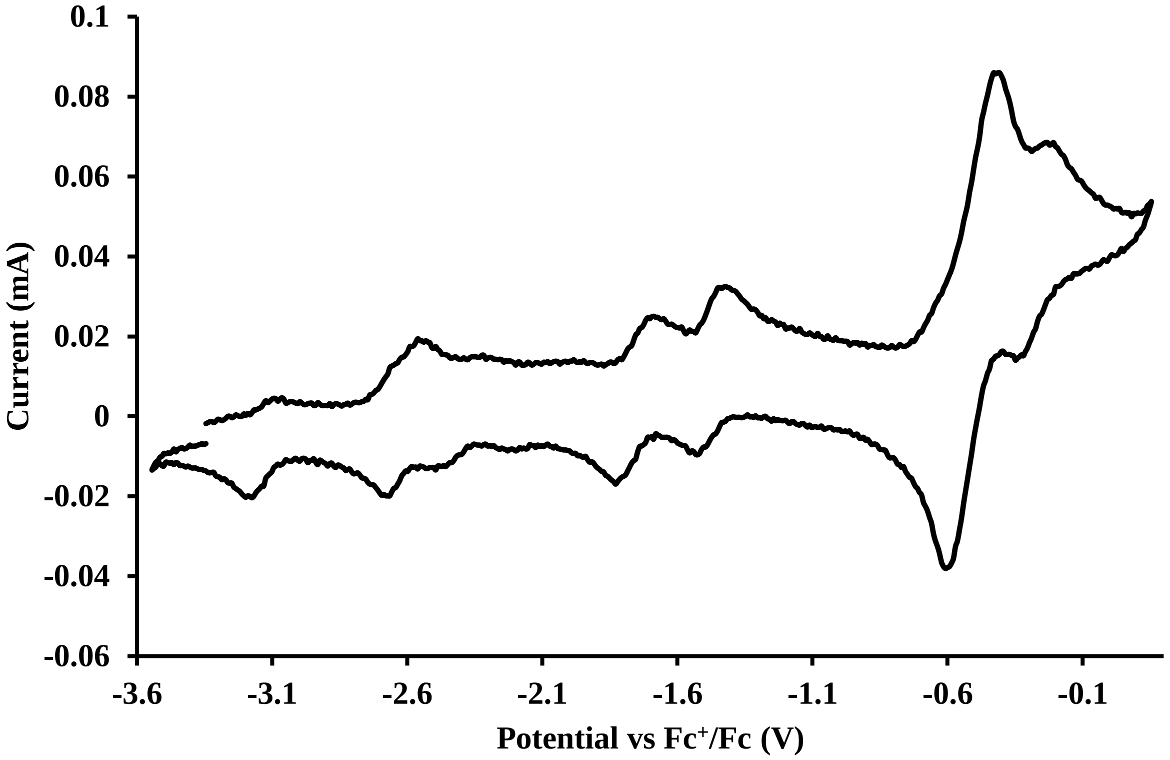

Figure S29: Voltammogram of [K(crypt)][$Cp^{tet}{}_3U$] at v = 200 mV/s in 100 mM $TBABPh_4$ / THF. The event centered at −0.495 V is due to internal standard $(C_5Me_5)_2Fe^{II}$.

Cp″3ThBr

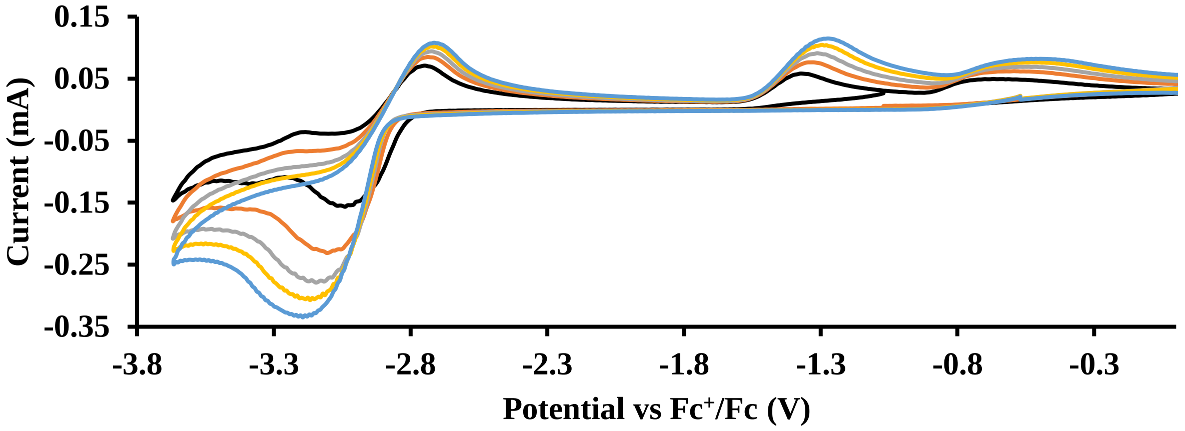

Figure S30: Voltammogram of 7.4 mM Cp″$_3$ThBr at ν = 200 (black), 400 (orange), 600 (grey), 800 (yellow) and 1000 (blue) mV/s in 100 mM TBABPh$_4$ / THF.

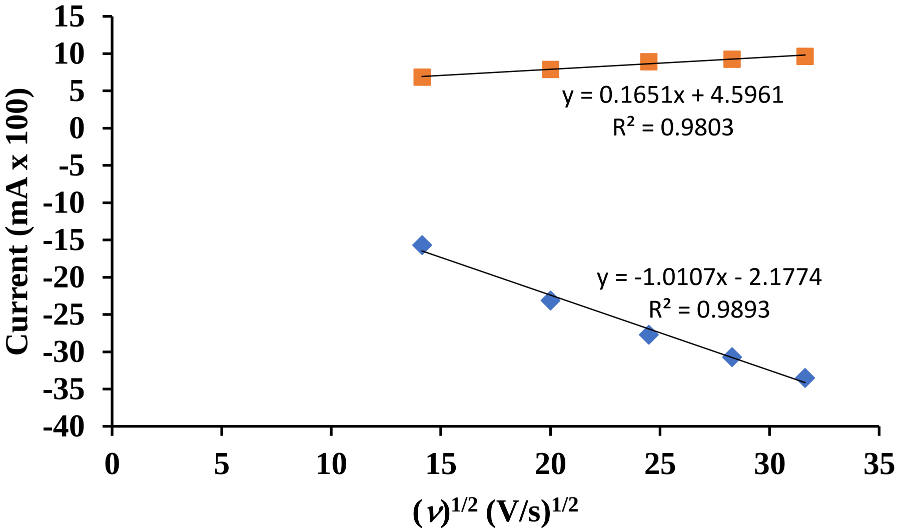

Figure S31: Scan rate dependence plot on the 4/3 couple of Cp″$_3$ThBr in 100 mM TBABPh$_4$ / THF.

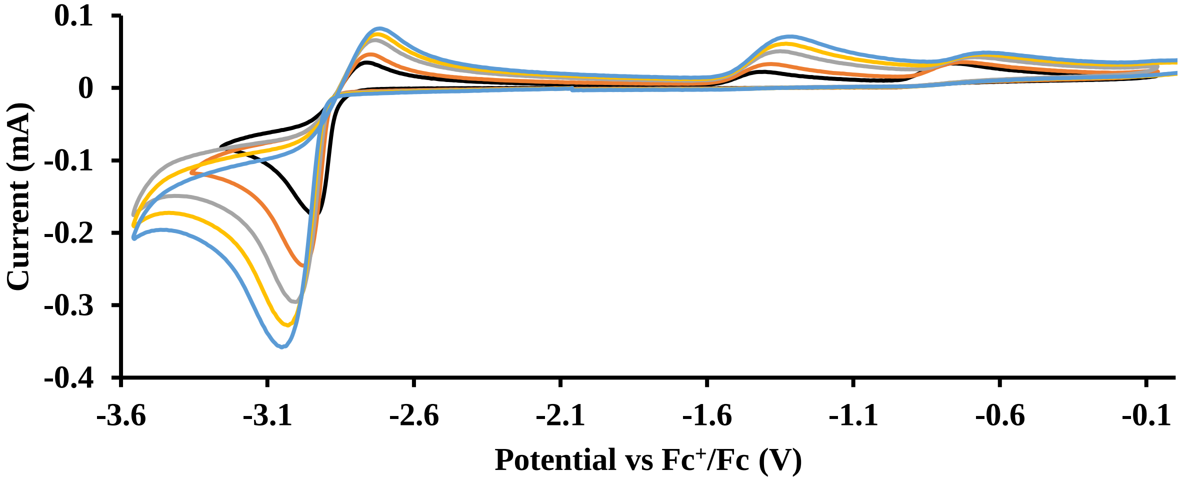

Figure S32: Voltammogram of 8.0 mM Cp″$_3$ThBr at ν = 200 (black), 400 (orange), 600 (grey), 800 (yellow) and 1000 (blue) mV/s in 200 mM $TBAPF_6$ / THF.

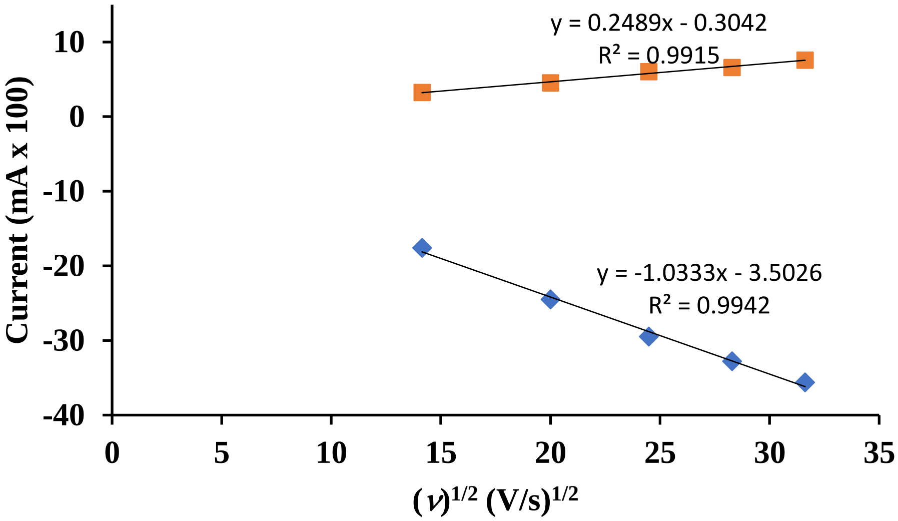

Figure S33: Scan rate dependence plot on the 4/3 couple of Cp″$_3$ThBr in 200 mM $TBAPF_6$ / THF.

Cp″$_3$ThCl

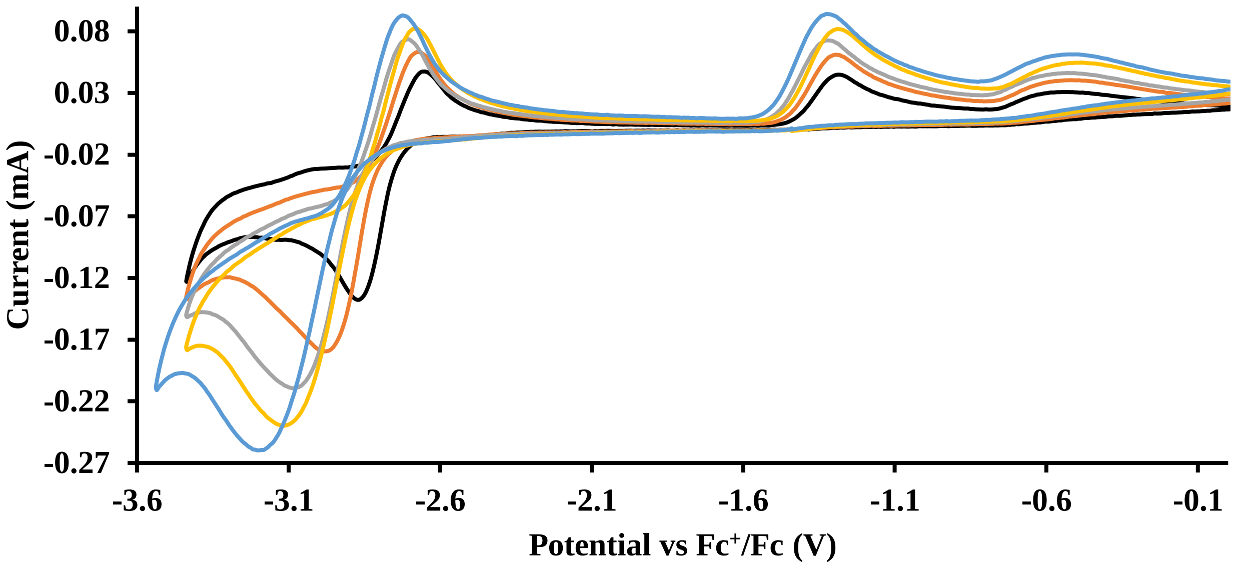

Figure S34: Voltammogram of 12 mM Cp″$_3$ThCl at ν = 200 (black), 400 (orange), 600 (grey), 800 (yellow) and 1000 (blue) mV/s in 100 mM TBABPh$_4$ / THF.

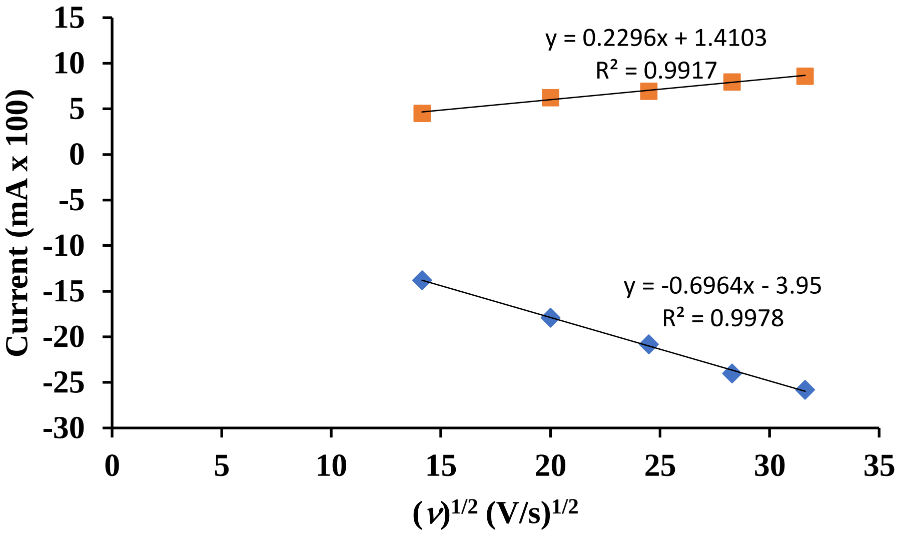

Figure S35: Scan rate dependence plot on the 4/3 couple of Cp″$_3$ThCl in 100 mM TBABPh$_4$ / THF.

Cp′$_3$ThCl

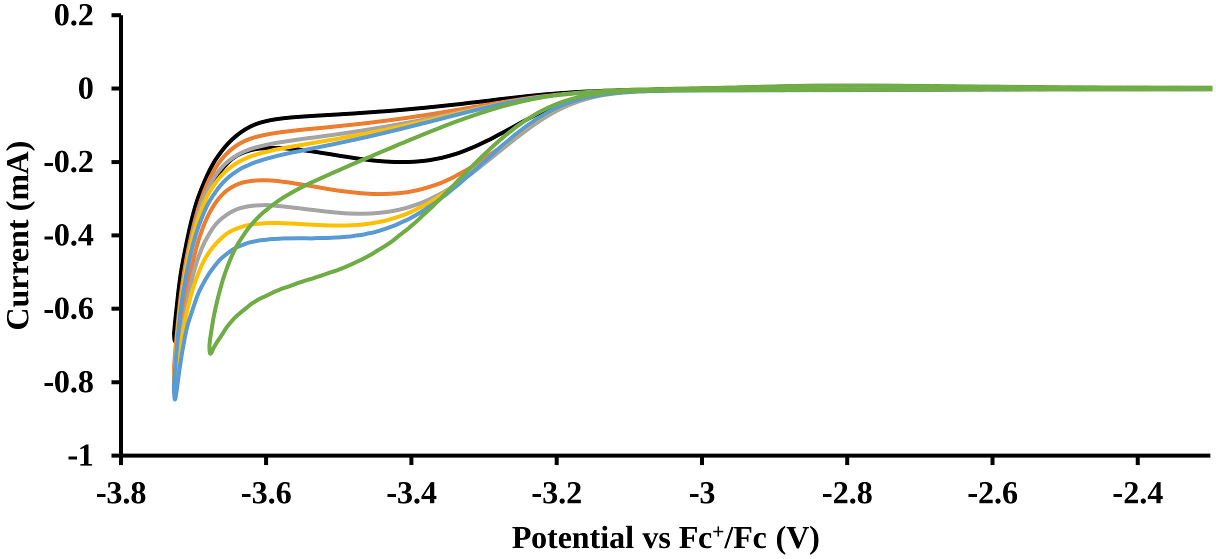

Figure S36: Voltammogram of 8.1 mM Cp′$_3$ThCl at v = 200 (black), 400 (orange), 600 (grey), 800 (yellow), 1000 (blue), and 2000 (green) mV/s in 200 mM TBAPF$_6$ / THF.

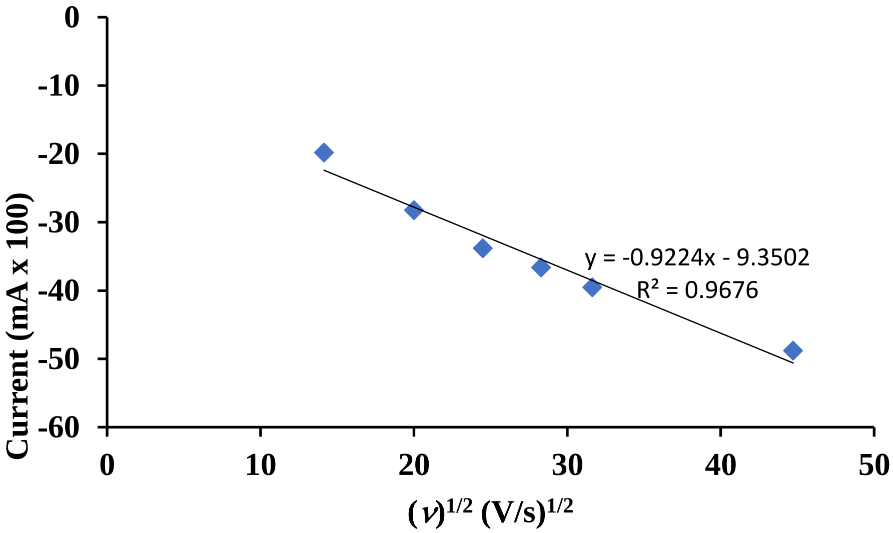

Figure S37: Scan rate dependence plot on the E_{PC} value of Cp′$_3$ThCl in 200 mM TBAPF$_6$ / THF.

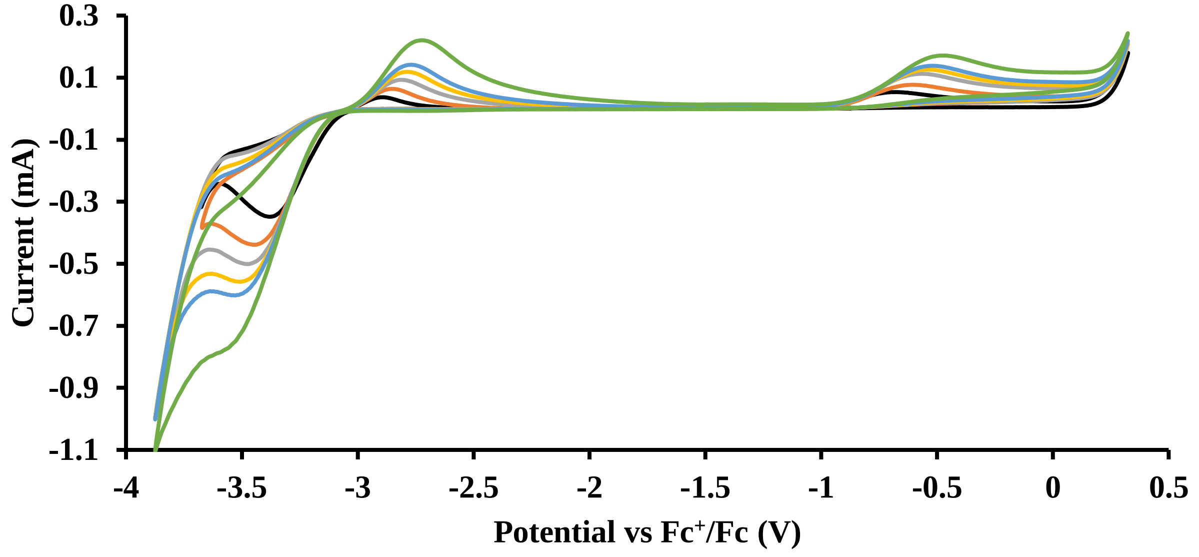

Figure S38: Voltammogram of 14 mM Cp′$_3$ThCl at v = 200 (black), 400 (orange), 600 (grey), 800 (yellow), 1000 (blue), and 2000 (green) mV/s in 100 mM TBABPh$_4$ / THF.

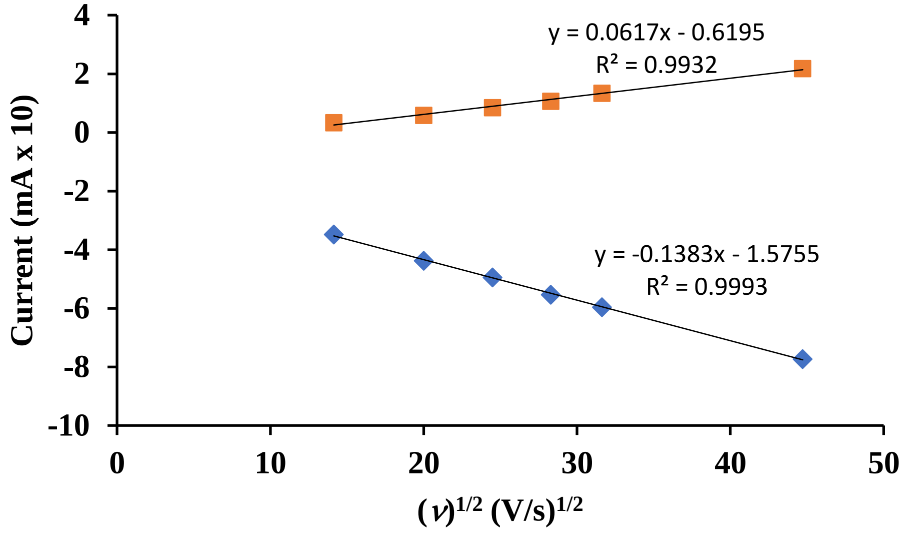

Figure S39: Scan rate dependence plot on the 4/3 couple of Cp′$_3$ThCl in 100 mM TBABPh$_4$ / THF.

Cp″₃Th

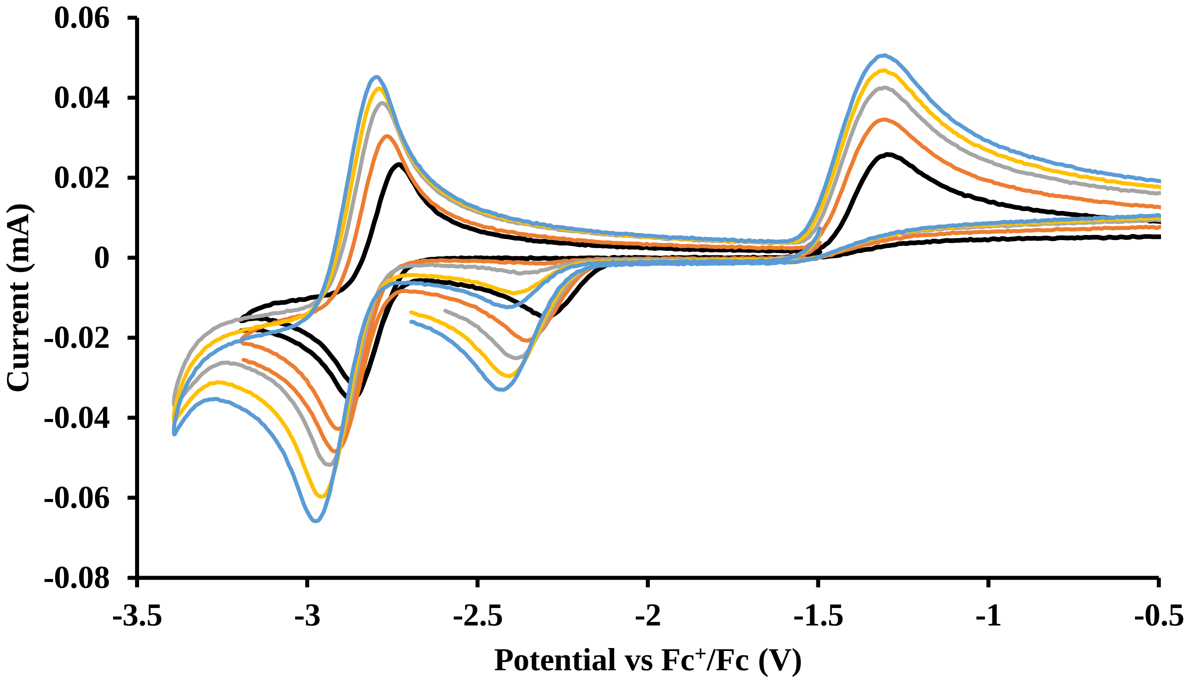

Figure S40: Voltammogram of 4.9 mM Cp″$_3$Th at ν = 200 (black), 400 (orange), 600 (grey), 800 (yellow) and 1000 (blue) mV/s in 100 mM TBABPh$_4$ / THF.

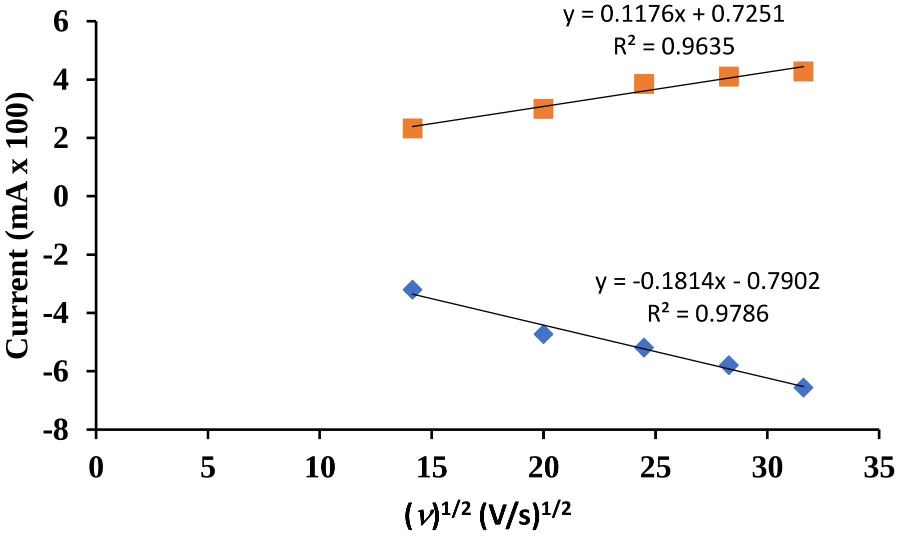

Figure S41: Scan rate dependence plot on the 3/2 couple of Cp″$_3$Th in 100 mM TBABPh$_4$ / THF.

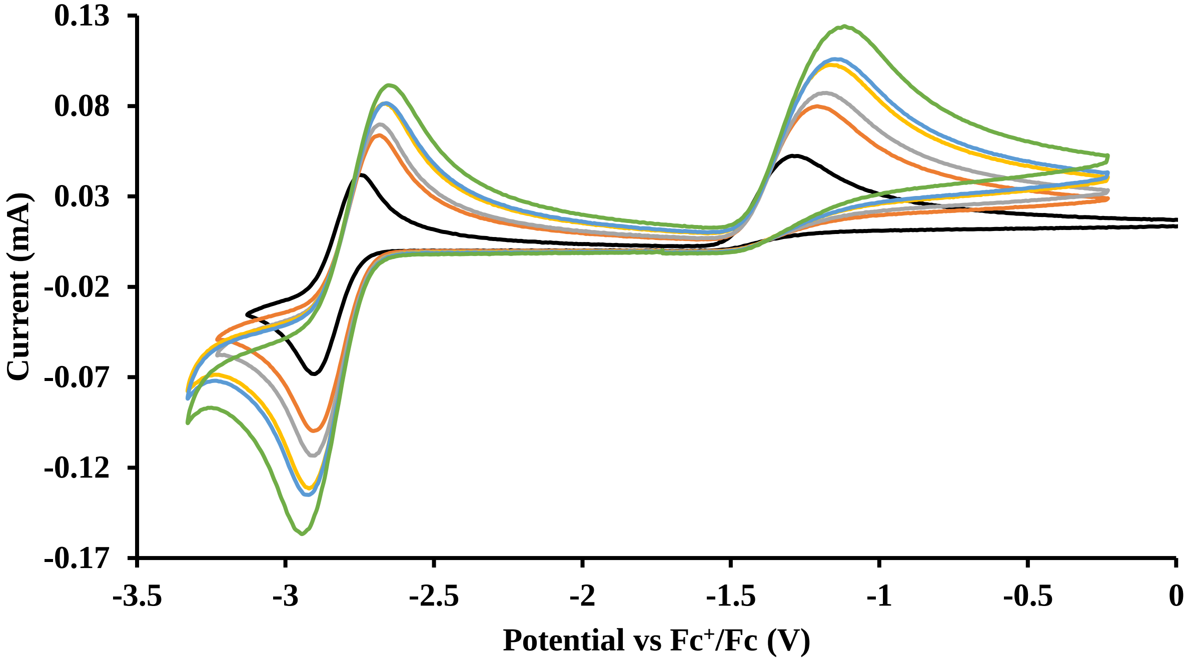

Figure S42: Voltammogram of 5.8 mM Cp″$_3$Th at *ν* = 200 (black), 400 (orange), 600 (grey), 800 (yellow), 1000 (blue), and 1500 (green) mV/s in 200 mM TBAPF$_6$ / THF.

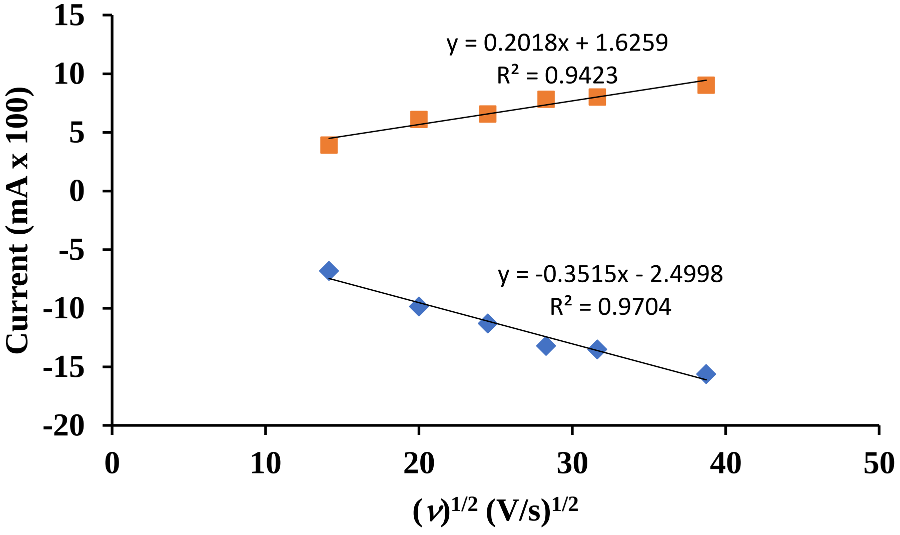

Figure S43: Scan rate dependence plot on the 3/2 couple of Cp″$_3$Th in 200 mM TBAPF$_6$ / THF.

$Cp^{tet}{}_3ThBr$

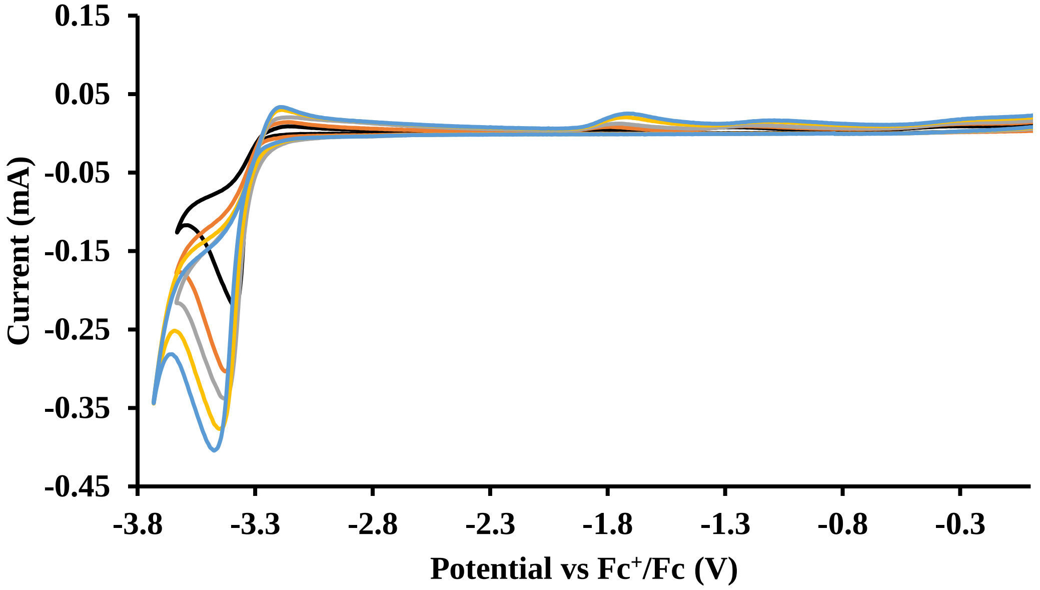

Figure S44: Voltammogram of 22 mM $Cp^{tet}{}_3ThBr$ at ν = 200 (black), 400 (orange), 600 (grey), 800 (yellow) and 1000 (blue) mV/s in 100 mM $TBABPh_4$ / THF.

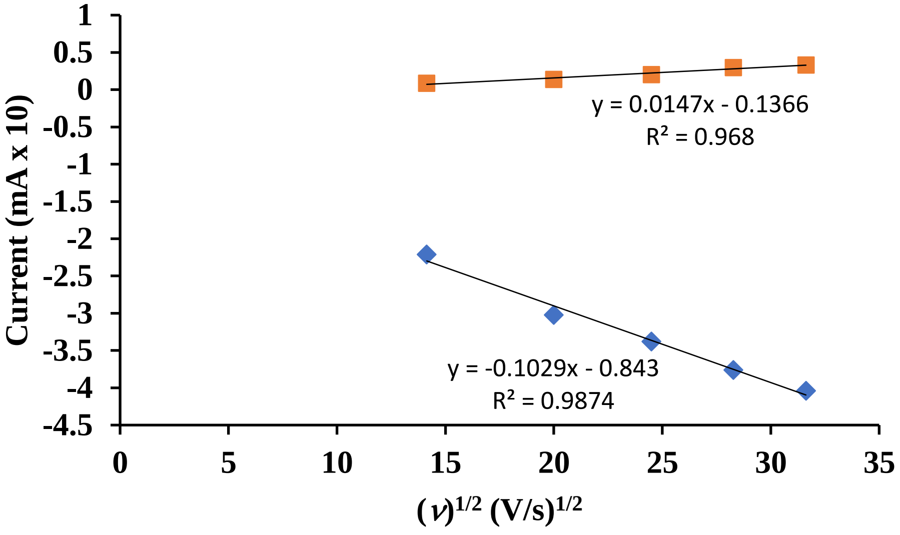

Figure S45: Scan rate dependence plot on the 4/3 couple of $Cp^{tet}{}_3ThBr$ in 100 mM $TBABPh_4$ / THF.

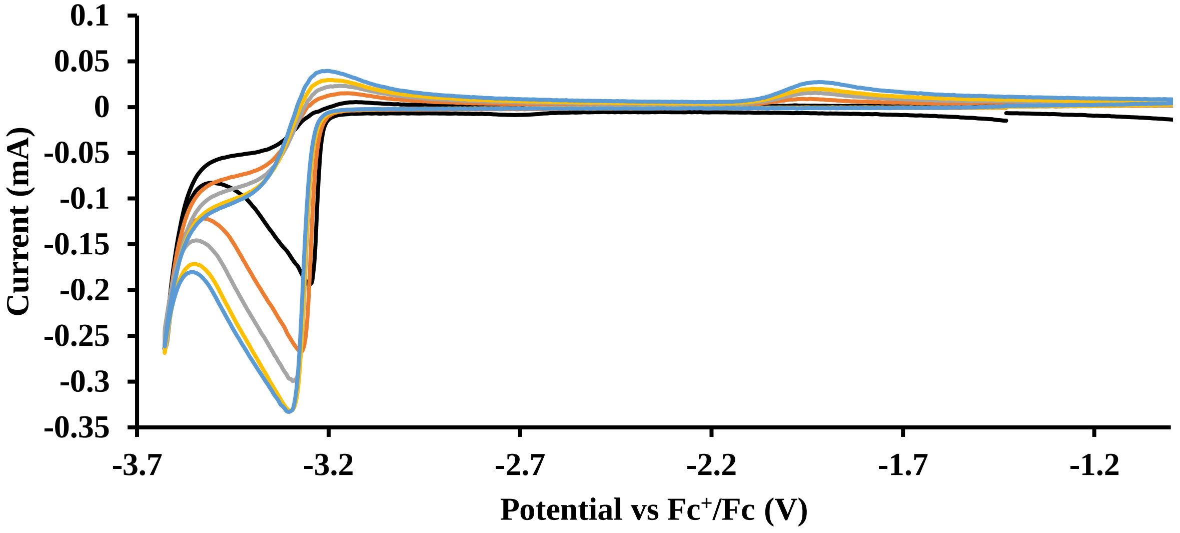

Figure S46: Voltammogram of 6.7 mM Cp[tet]$_3$ThBr at ν = 200 (black), 400 (orange), 600 (grey), 800 (yellow) and 1000 (blue) mV/s in 200 mM TBAPF$_6$ / THF.

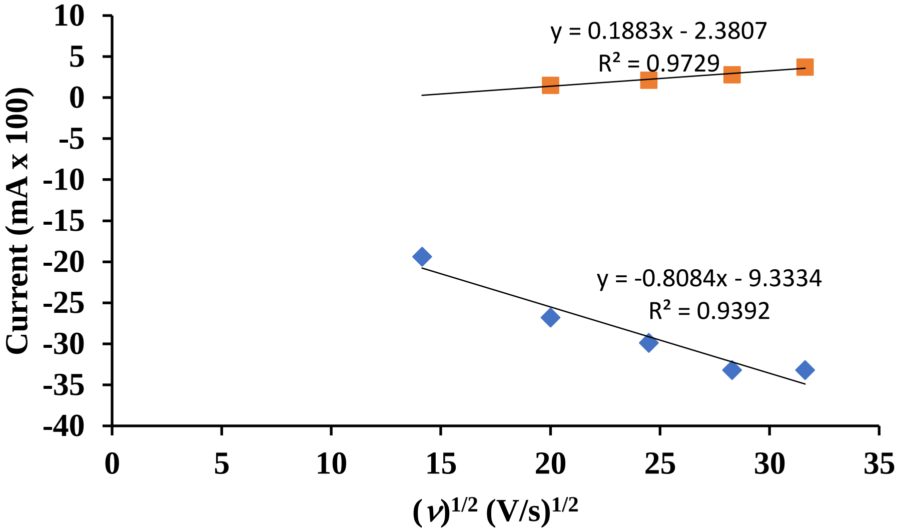

Figure S47: Scan rate dependence plot on the 4/3 couple of Cp[tet]$_3$ThBr in 200 mM TBAPF$_6$ / THF.

Cp^{tet}$_3$Th

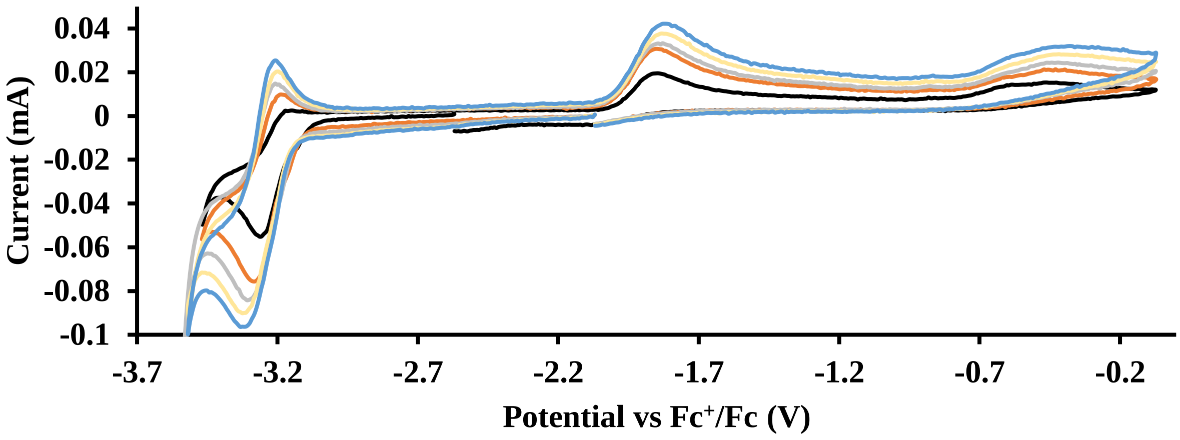

Figure S48: Voltammogram of 6.7 mM Cp^{tet}$_3$Th at ν = 200 (black), 400 (orange), 600 (grey), 800 (yellow) and 1000 (blue) mV/s in 100 mM TBABPh$_4$ / THF.

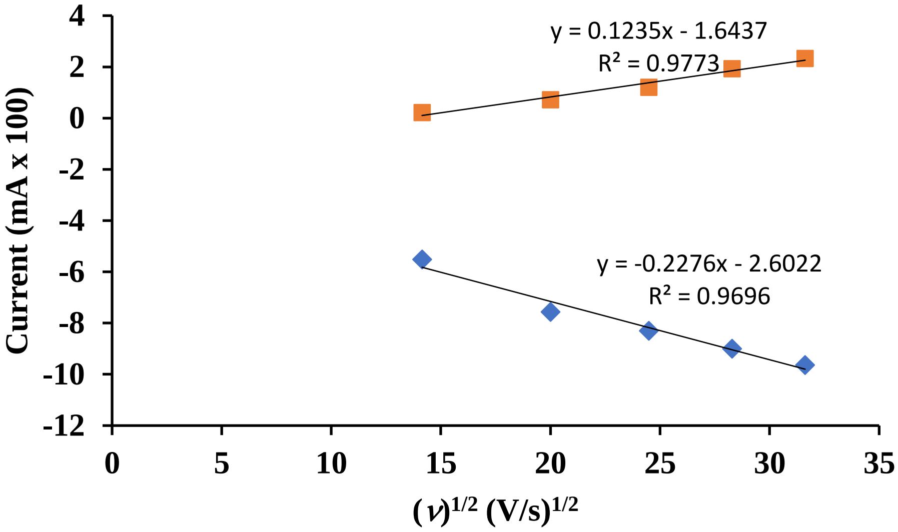

Figure S49: Scan rate dependence plot on the 3/2 couple of Cp^{tet}$_3$Th in 200 mM TBABPh$_4$ / THF.

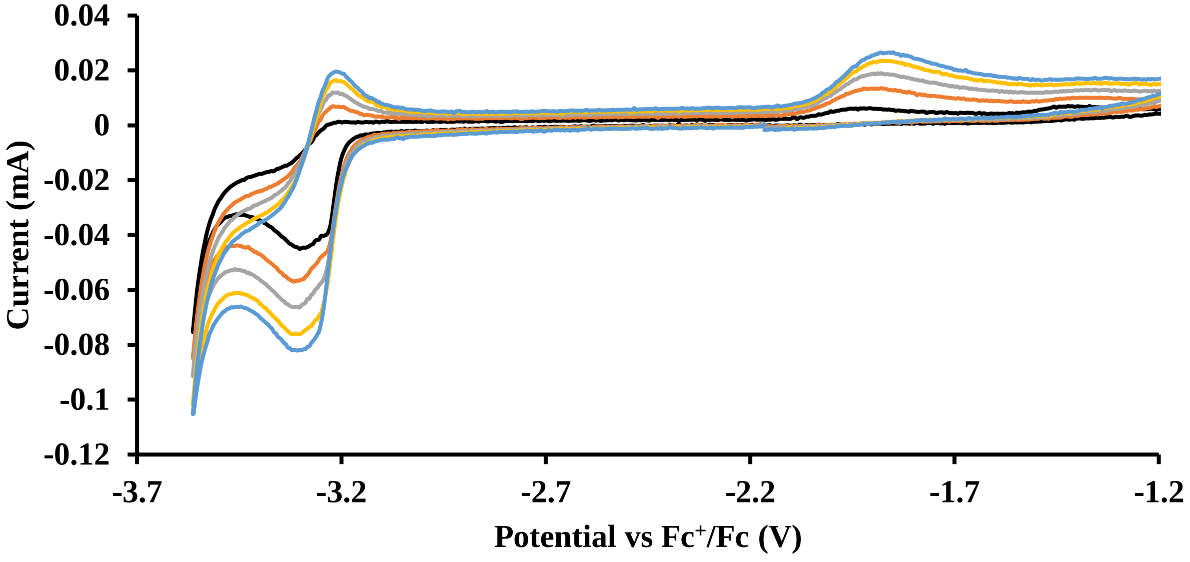

Figure S50: Voltammogram of 10 mM $Cp^{tet}{}_3Th$ at ν = 200 (black), 400 (orange), 600 (grey), 800 (yellow) and 1000 (blue) mV/s in 200 mM $TBAPF_6$ / THF.

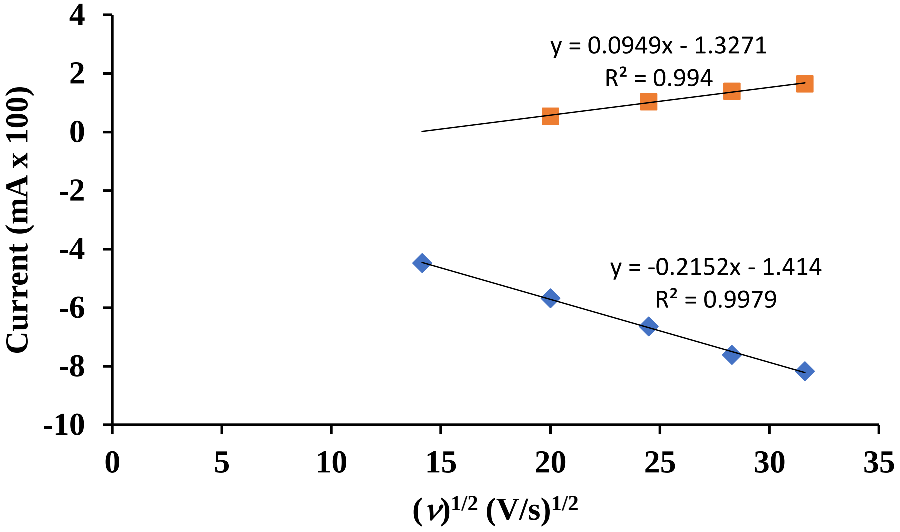

Figure S51: Scan rate dependence plot on the 3/2 couple of $Cp^{tet}{}_3Th$ in 200 mM $TBAPF_6$ / THF.

[K(crown)(THF)$_2$][Cp″$_3$Th]

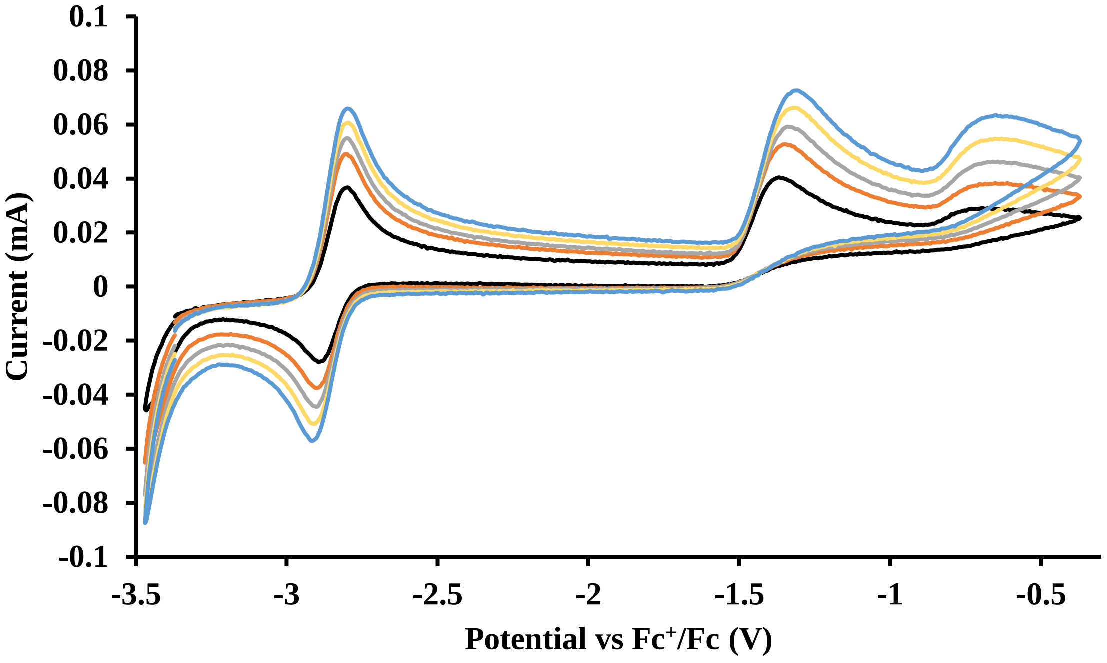

Figure S52: Voltammogram of 4.6 mM [K(crown)(THF)$_2$][Cp″$_3$Th] at v = 200 (black), 400 (orange), 600 (grey), 800 (yellow) and 1000 (blue) mV/s in 100 mM TBABPh$_4$ / THF.

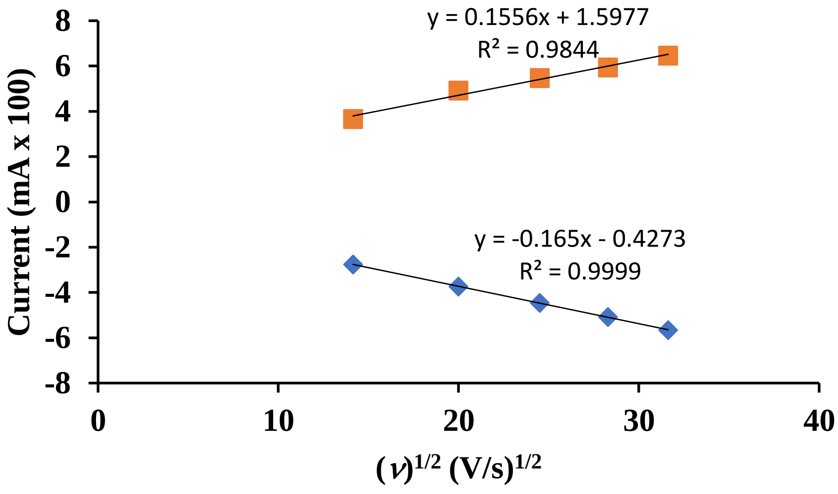

Figure S53: Scan rate dependence plot on the 3/2 couple of [K(crown)(THF)$_2$][Cp″$_3$Th] in 100 mM TBABPh$_4$ / THF.

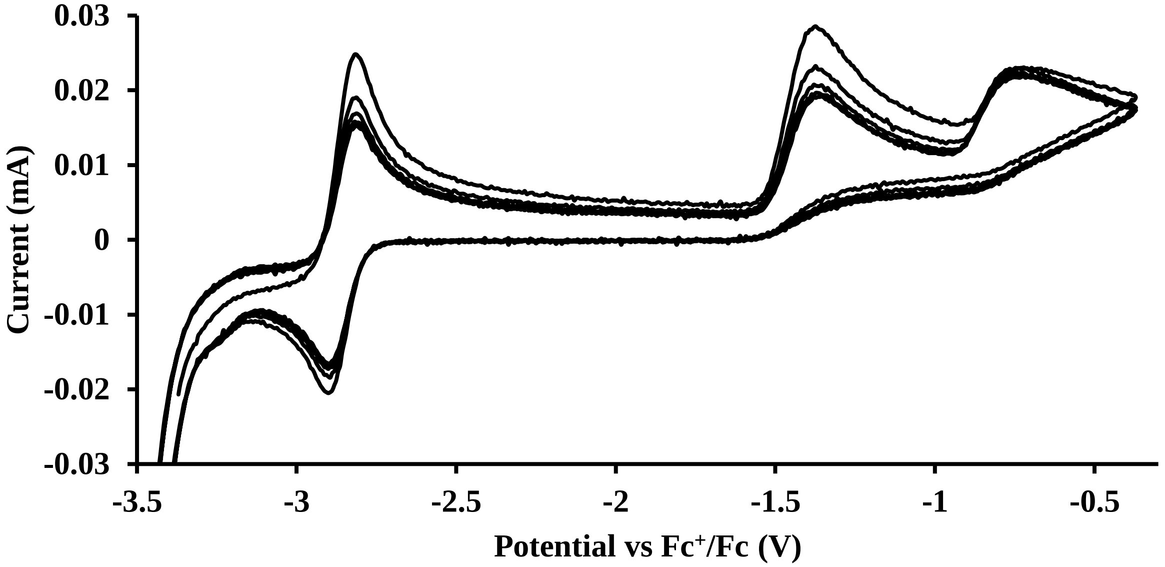

Figure S54: Voltammogram of 4.6 mM [K(crown)(THF)$_2$][Cp″$_3$Th] at v = 200 mV/s over 5 cycles in 100 mM TBABPh$_4$ / THF.

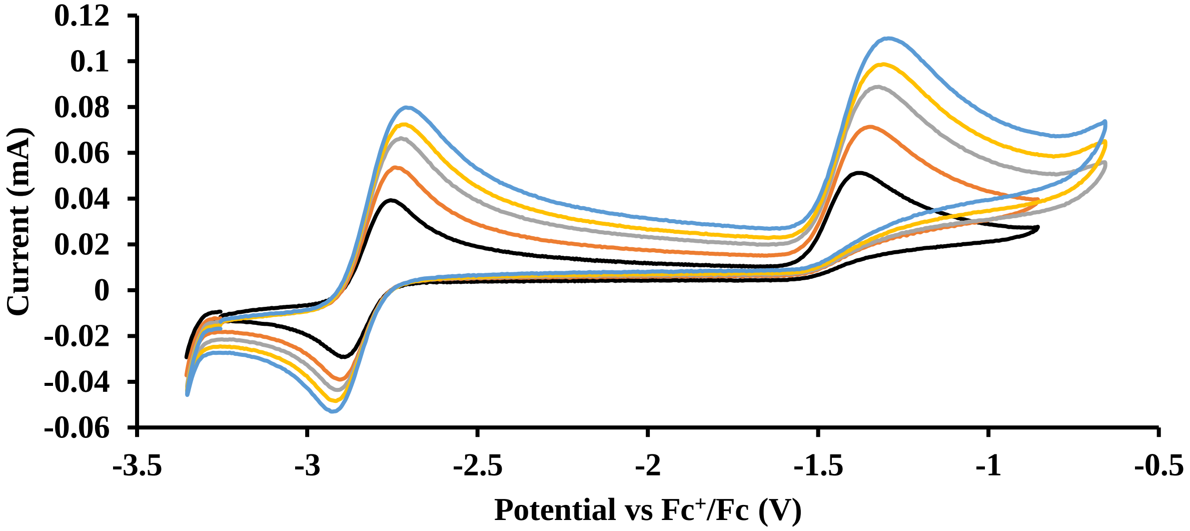

Figure S55: Voltammogram of 4.3 mM [K(crown)(THF)$_2$][Cp″$_3$Th] at v = 200 (black), 400 (orange), 600 (grey), 800 (yellow) and 1000 (blue) mV/s in 200 mM TBAPF$_6$ / THF.

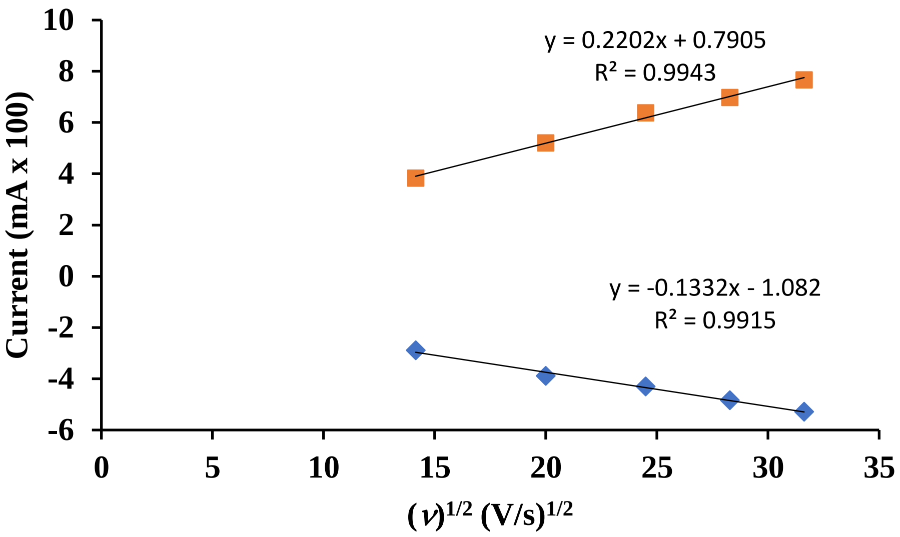

Figure S56: Scan rate dependence plot on the 3/2 couple of [K(crown)(THF)$_2$][Cp″$_3$Th] in 200 mM TBAPF$_6$ / THF.

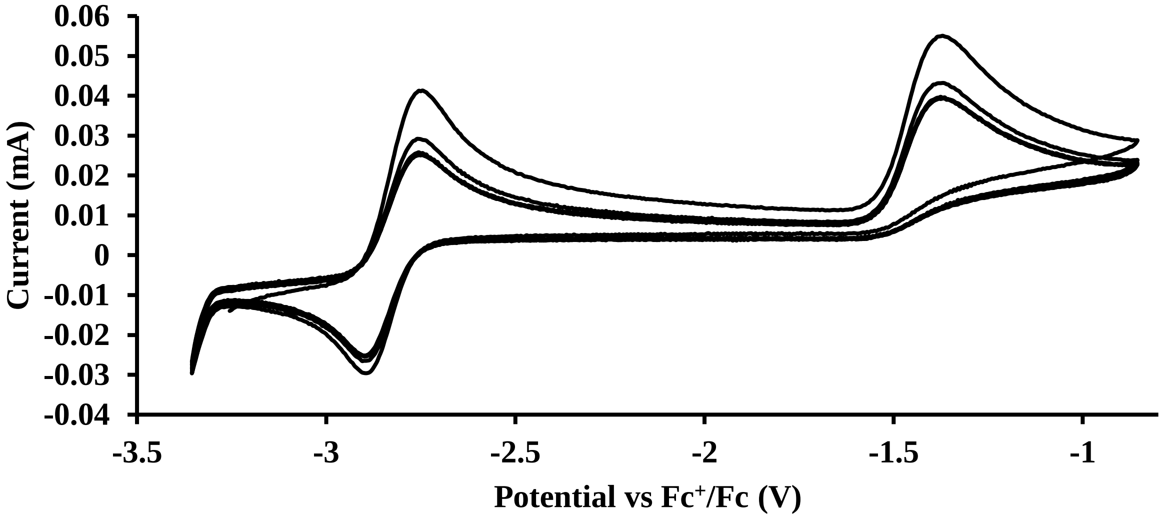

Figure S57: Voltammogram of 4.3 mM [K(crown)(THF)$_2$][Cp″$_3$Th] at ν = 200 mV/s over 5 cycles in 200 mM TBAPF$_6$ / THF.

[K(crypt)][Cp″$_3$Th]

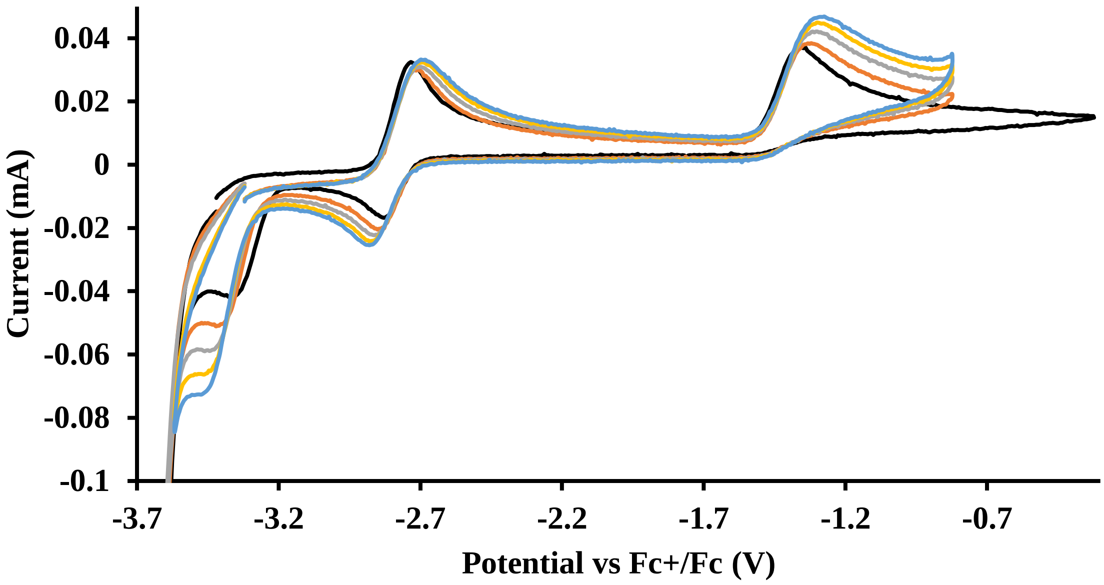

Figure S58: Voltammogram of 3.1 mM [K(crypt)][Cp″$_3$Th] at ν = 200 (black), 400 (orange), 600 (grey), 800 (yellow) and 1000 (blue) mV/s in 200 mM TBAPF$_6$ / THF.

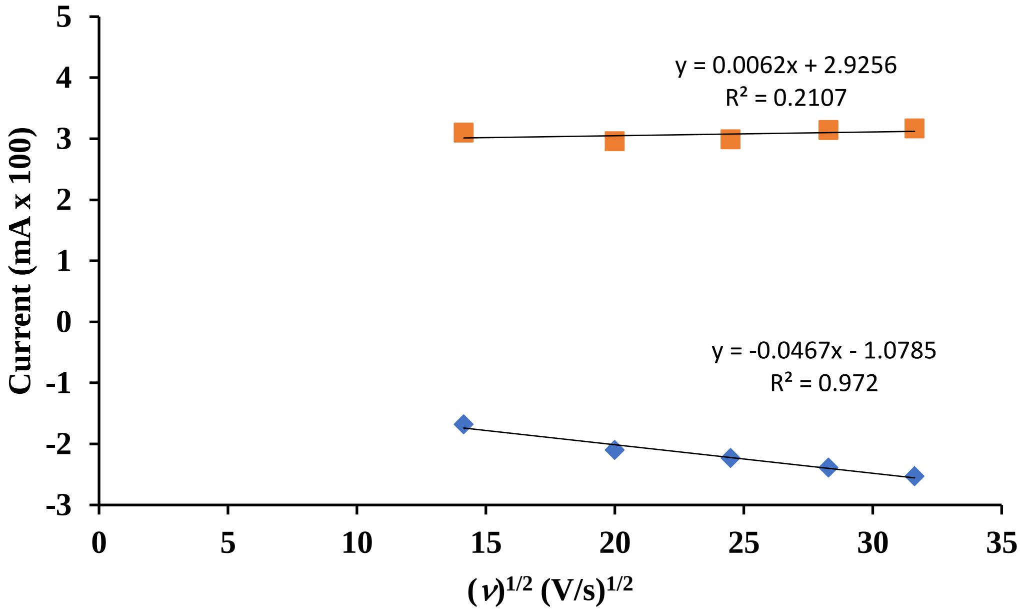

Figure S59: Scan rate dependence plot on the 3/2 couple of [K(crypt)][Cp″$_3$Th] in 200 mM TBAPF$_6$ / THF.

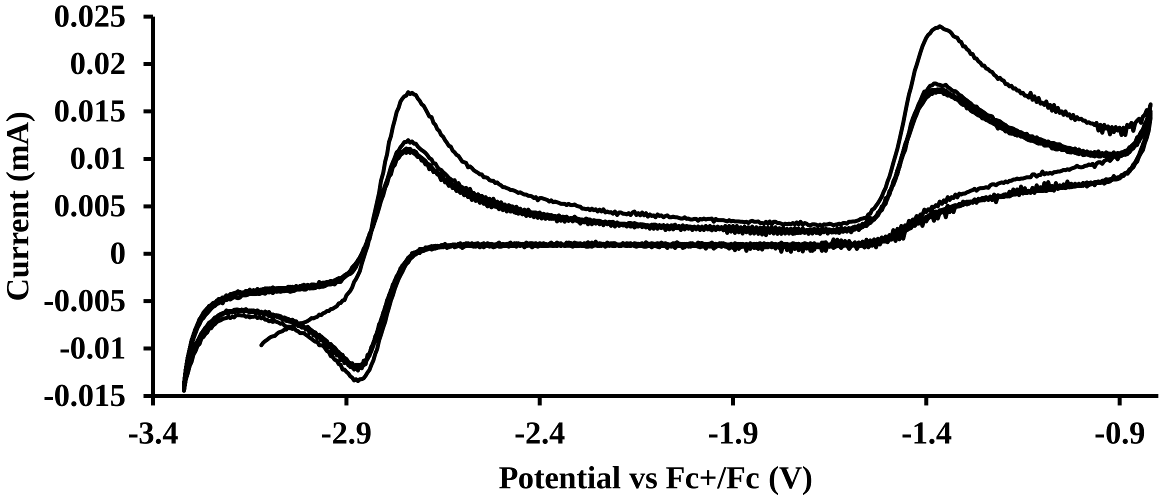

Figure S60: Voltammogram of 3.1 mM [K(crypt)][Cp″$_3$Th] at v = 200 mV/s over 5 cycles in 200 mM $TBAPF_6$ / THF.

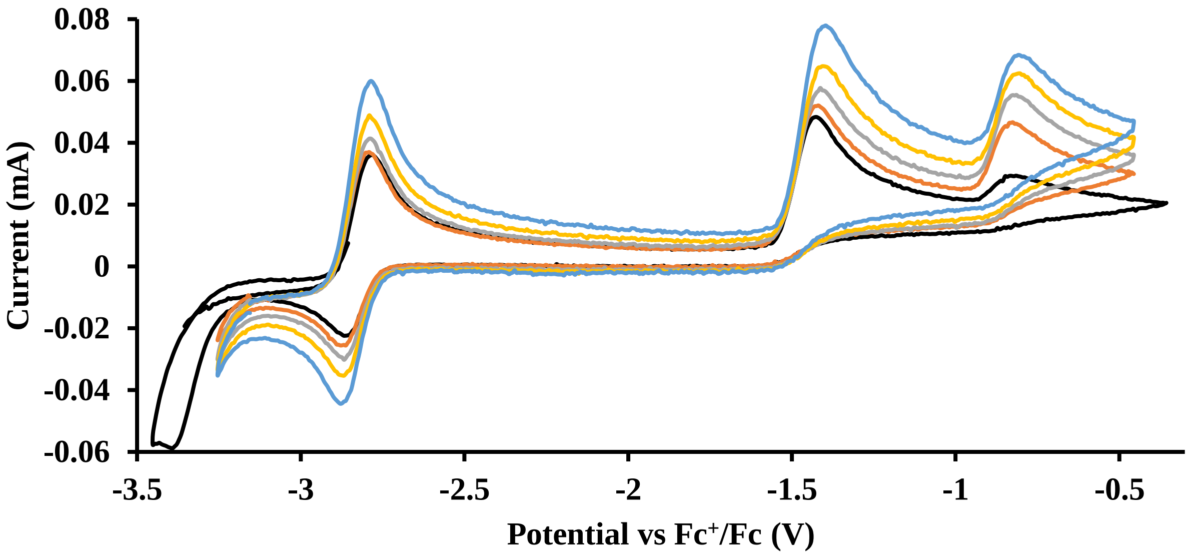

Figure S61: Voltammogram of 3.9 mM [K(crypt)][Cp″$_3$Th] at v = 200 (black), 400 (orange), 600 (grey), 800 (yellow) and 1000 (blue) mV/s in 100 mM $TBABPh_4$ / THF.

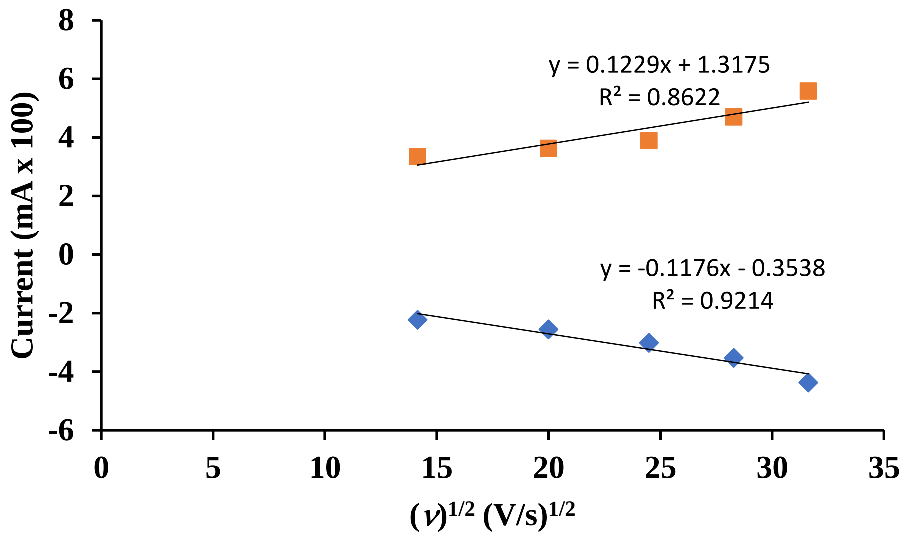

Figure S62: Scan rate dependence plot on the 3/2 couple of [K(crypt)][Cp″$_3$Th] in 100 mM TBABPh$_4$ / THF.

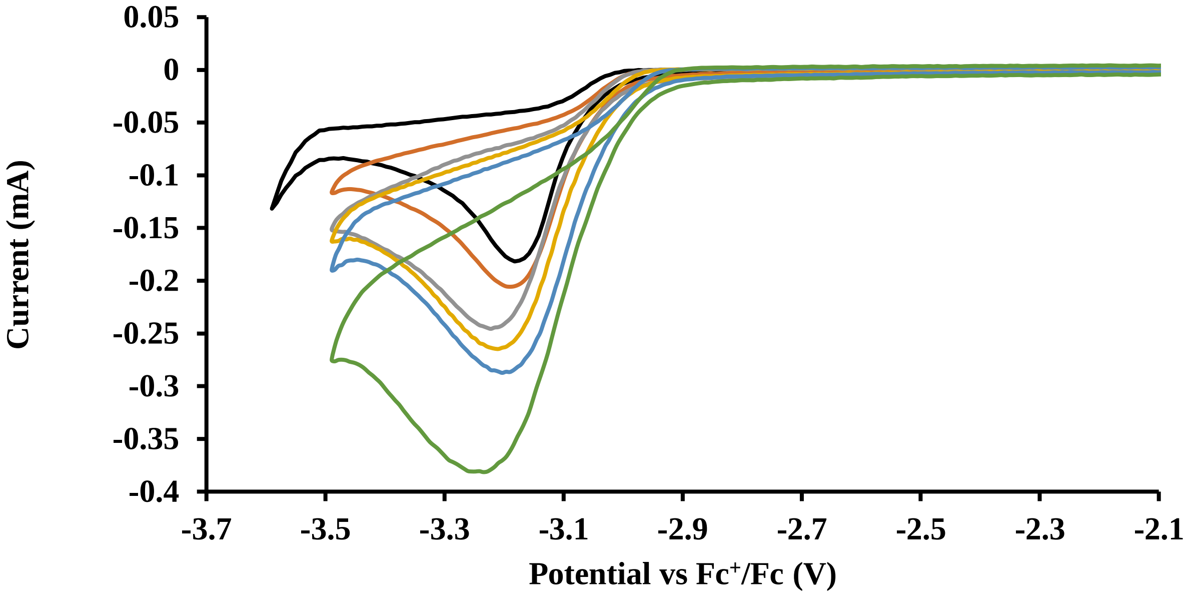

Figure S63: Voltammogram of xx mM Cp′$_3$Th^{IV}Br at ν = 200 (black), 400 (orange), 600 (grey), 800 (yellow), 1000 (blue) and 2000 (green) mV/s in 100 mM TBABPh$_4$ / THF.

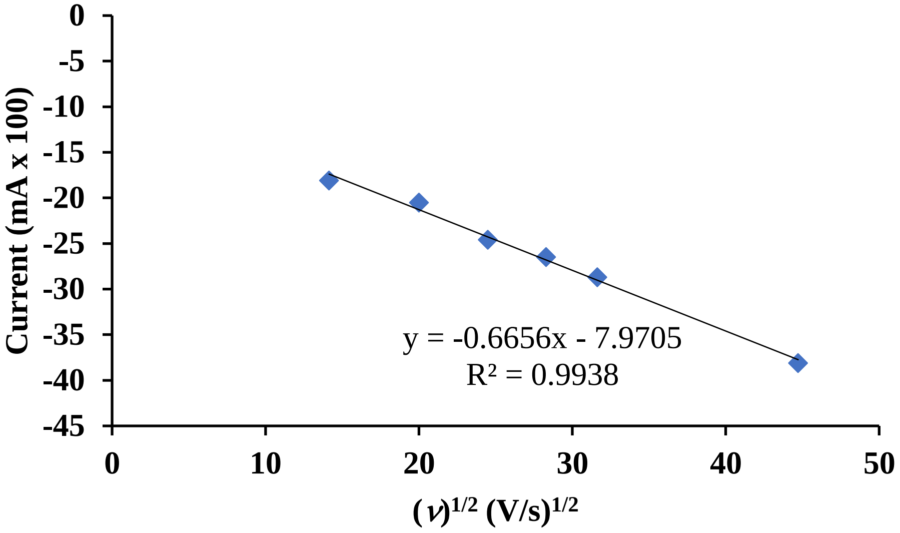

Figure S64: Scan rate dependence plot on the cathodic event of Cp′$_3$Th^{IV}Br in 100 mM TBABPh$_4$ / THF.

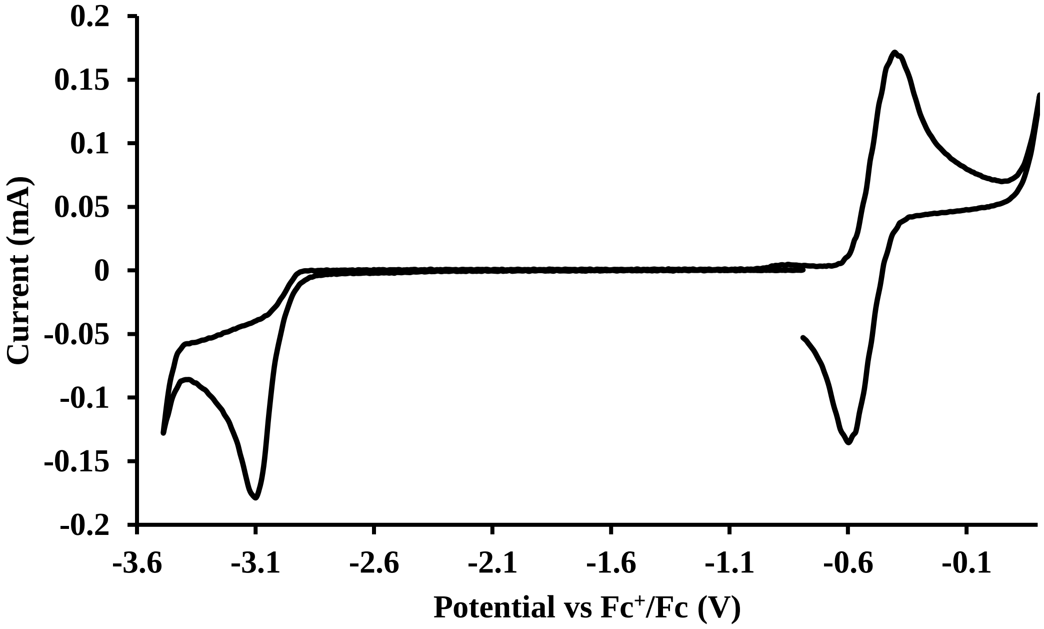

Figure S65: Voltammogram of 15 mM Cp′$_3$Th^{IV}Br and 14.6 mM $(C_5Me_5)_2Fe$ in 100 mM TBABPh$_4$ / THF. The ratio of current passed for Cp′$_3$Th^{IV}Br to $(C_5Me_5)_2Fe$ is 0.77, suggesting a one-electron process is occurring for Cp′$_3$Th^{IV}Br.

Potassium Cyclopentadienide Salts. To probe the ligand effects further and to help identify the events around −1.5 V in the voltammograms of the thorium complexes, the voltammograms for the ligands as potassium salts, KCp^{tet}, KCp, KCp′, and KCp″, were examined with [$^{n}Bu_4N$][PF_6] as supporting electrolyte. Irreversible anodic processes were observed for each species, Table S2 and Figure S56. The irreversibility of these events is consistent with a chemical process occurring after oxidation such as dimerization of the *in-situ* generated radical.[18] This series of reduction potentials for simple potassium cyclopentadienyl salts does not match the trend observed in the thorium complexes above and in related zirconium systems.[19] However, it was noted that trends in cyclopentadienyl donor strength are system dependent.

Table S2: Peak anodic potentials for potassium cyclopentadienide salts

	E_{PA} (V)
KCp^{tet}	−1.17
KCp	−0.50
KCp′	−0.63
KCp″	−0.71
[K(crown)][Cp″]	−0.76
[K(crypt)][Cp″]	−0.77

Addition of one equivalent of crown to KCp″ shifts the event slightly negative, Figure S57, from −0.71 V to −0.76 V. The addition of crypt to KCp″ shifts the oxidation event in a similar fashion to −0.77 V. Although the structure of KCp″ in THF in the presence of the chelate and the supporting electrolyte is not known, information on the chelate-potassium interaction is known in the solid state. The X-ray crystal structure of [K(crypt)][Cp′] shows a distinct ion pair in the solid state with no K^{+}….$[Cp']^{1-}$ interaction.[20] However, the crystal structure of [K(crown)][Cp″] is reported as part of this study, Figure S58, and shows clear interaction of the K^{+} ion with the $[Cp'']^{1-}$ anion in the solid state. The shifted potentials of the solutions containing chelate are consistent

with chelation of the potassium ion in solution diminishing the interaction with the cyclopentadienyl ring, thus facilitating oxidation compared to KCp″. It would be expected, based on the solid state structures, that crypt would have a greater effect than crown, but in solution, it is possible that the crown system contains solvated $[K(crown)(THF)_x]^{1+}$ moieties and thus has less interaction with the cyclopentadienyl than expected.[21–27] The [K(crown)][Cp″]/ferrocene system was also examined in THF-d_8 by ^{1}H NMR spectroscopy and no evidence for ligand exchange between KCp″ and ferrocene was observed.

The irreversible anodic events around −1.5 V observed in the thorium systems are thus assigned as cyclopentadienide-based events. Interestingly, these types of irreversible anodic events were not observed in the cyclopentadienyl-uranium systems. Previous electrochemical studies of organoactinide complexes also observed irreversible anodic events in thorium complexes and not the analogous uranium systems that were thus attributed to ligand-based processes or ligand-distribution processes.[28–31] Clearly, the Lewis acidity of the metal influences the potential for these cyclopentadienide oxidations. There will be a shift in the observed oxidation potential whether the cyclopentadienyl ring is bound to K^+, $[K(chelate)]^+$, or An^{n+}.

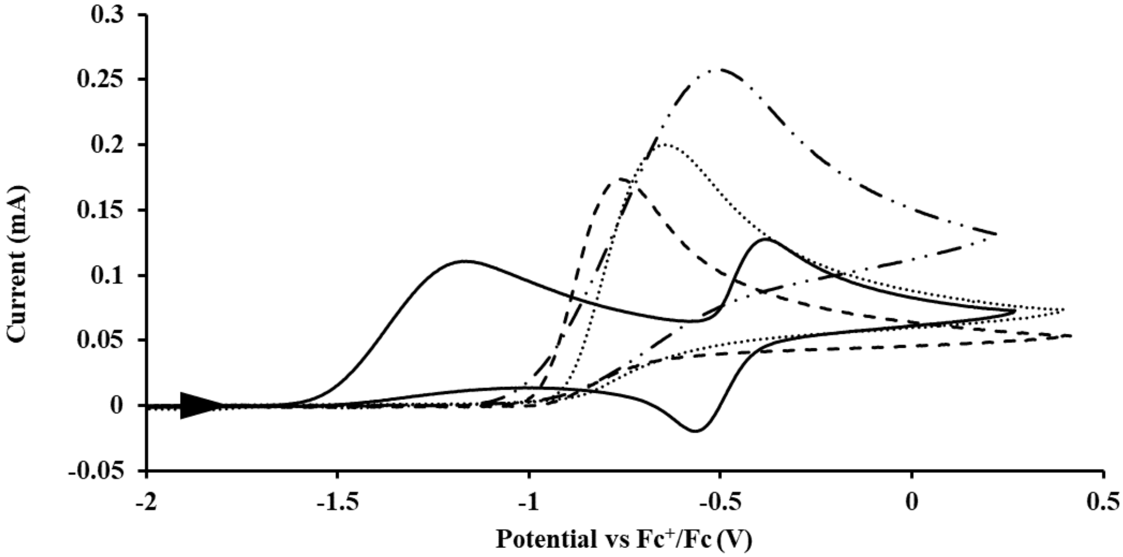

Figure S66: Voltammogram of KCp^{tet} (solid, 15 mM), KCp″ (dashed, 17 mM), KCp′ (dotted, 14 mM), and KCp (dotted dash, 22 mM) at ν = 200 mV/s in 200 mM [$^{n}Bu_4N$][PF_6] / THF. The event centered at −0.495 V in the voltammogram of KCp^{tet} is due to internal standard $(C_5Me_5)_2Fe^{II}$.

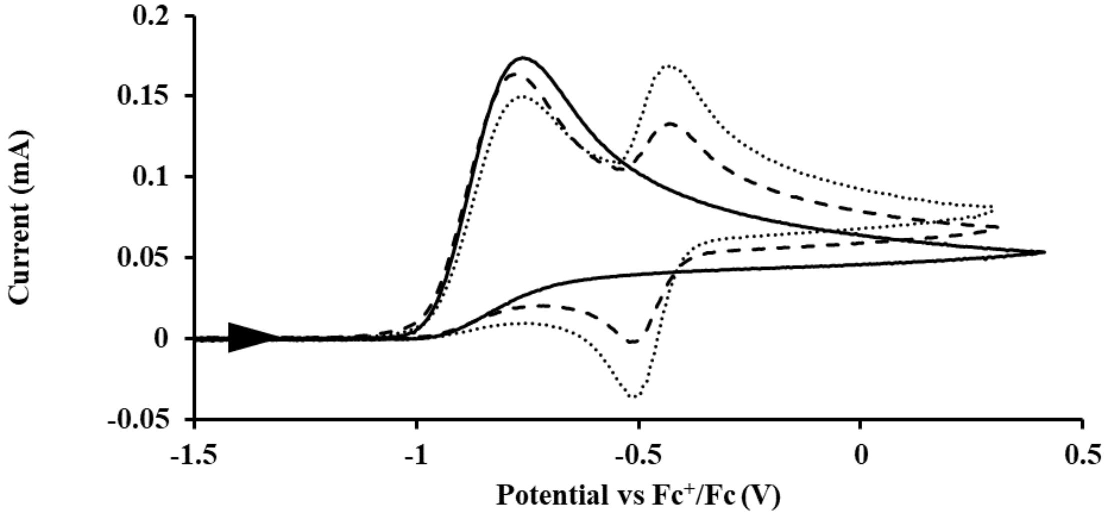

Figure S67: Voltammogram of 17 mM KCp″ (solid), [K(crown)][Cp″] (dashed), and [K(crypt)][Cp″] (dotted) at 200 mV/s in 200 mM [$^{n}Bu_4N$][PF_6] / THF. The event centered at −0.495 V is due to internal standard $(C_5Me_5)_2Fe^{II}$.

Crystallographic Details

X-ray Data Collection, Structure Solution and Refinement for [K(crown)][Cp″].

A blue crystal of approximate dimensions 0.129 x 0.144 x 0.191 mm was mounted in a cryoloop and transferred to a Bruker SMART APEX II diffractometer. The APEX2[32] program package was used to determine the unit-cell parameters and for data collection (180 sec/frame scan time for a hemisphere of diffraction data). The raw frame data was processed using SAINT[33] and SADABS[34] to yield the reflection data file. Subsequent calculations were carried out using the SHELXTL[35] program. The diffraction symmetry was 2/*m* and the systematic absences were consistent with the monoclinic space group $P2_1/c$ that was later determined to be correct.

The structure was solved by direct methods and refined on F^2 by full-matrix least-squares techniques. The analytical scattering factors[36] for neutral atoms were used throughout the analysis. Hydrogen atoms were included using a riding model. There were two molecules of the formula-unit present (Z = 8). C(41) and C(42) were disordered and included using multiple components with partial site-occupancy factors.

Least-squares analysis yielded wR2 = 0.0996 and Goof = 1.010 for 608 variables refined against 14783 data (0.73 Å), R1 = 0.0500 for those 10223 data with $I > 2.0\sigma(I)$. The structure was refined as a two-component twin (BASF = 0.43).

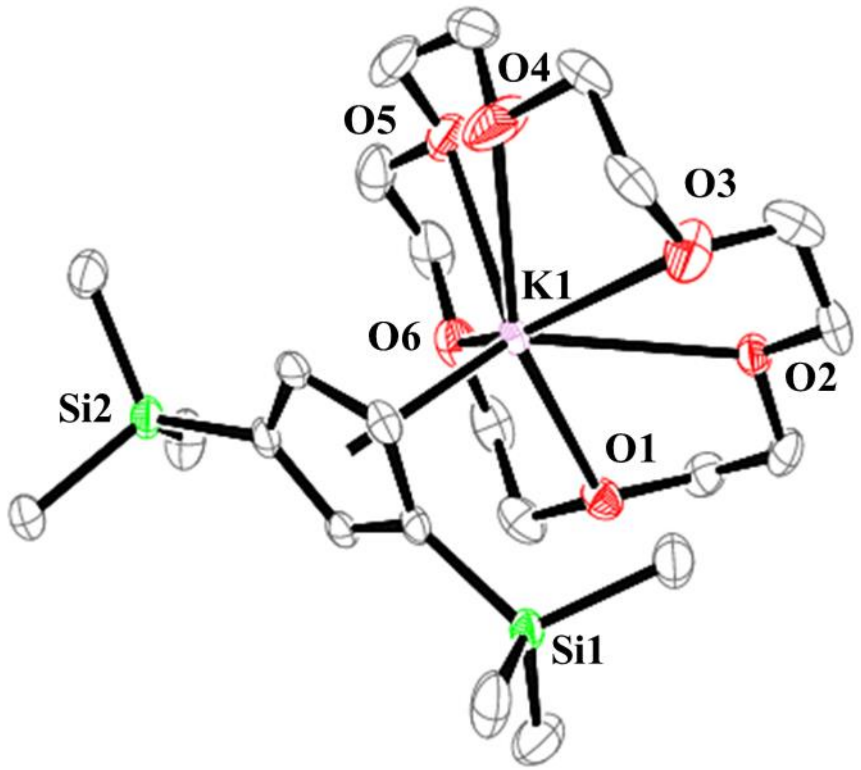

Figure S68: Thermal ellipsoid plot of [K(crown)][Cp″] drawn at the 50% probability level. Hydrogen atoms have been removed for clarity.

Table S2. Crystal data and structure refinement for [K(crown)][Cp″].

Identification code	rrl39	
Empirical formula	$C_{23} H_{45} K O_6 Si_2$	
Formula weight	512.87	
Temperature	88(2) K	
Wavelength	0.71073 Å	
Crystal system	Monoclinic	
Space group	$P2_1/c$	
Unit cell dimensions	a = 18.737(4) Å	α = 90°.
	b = 18.571(4) Å	β = 90.149(3)°.
	c = 16.803(3) Å	γ = 90°.
Volume	5847(2) Å^3	
Z	8	
Density (calculated)	1.165 Mg/m^3	
Absorption coefficient	0.295 mm^{-1}	

F(000)	2224
Crystal color	blue
Crystal size	0.191 x 0.144 x 0.129 mm^3
Theta range for data collection	1.212 to 29.206°
Index ranges	-25 ≤ *h* ≤ 25, -24 ≤ *k* ≤ 25, -22 ≤ *l* ≤ 23
Reflections collected	51430
Independent reflections	14783 [R(int) = 0.0719]
Completeness to theta = 25.500°	100.0 %
Absorption correction	Semi-empirical from equivalents
Max. and min. transmission	0.8016 and 0.7190
Refinement method	Full-matrix least-squares on F^2
Data / restraints / parameters	14783 / 0 / 608
Goodness-of-fit on F^2	1.010
Final R indices [I>2sigma(I) = 10223 data]	R1 = 0.0500, wR2 = 0.0872
R indices (all data, 0.73 Å)	R1 = 0.0964, wR2 = 0.0996
Largest diff. peak and hole	0.442 and -0.493 e.Å^{-3}

Table S3. Bond lengths [Å] and angles [°] for [K(crown)][Cp″].

K(1)-Cnt1	2.904	Si(2)-C(9)	1.862(3)
K(1)-O(3)	2.792(2)	Si(2)-C(11)	1.870(3)
K(1)-O(2)	2.857(2)	Si(2)-C(10)	1.874(3)
K(1)-O(6)	2.8574(19)	O(1)-C(12)	1.425(3)
K(1)-O(5)	2.889(2)	O(1)-C(23)	1.432(3)
K(1)-O(1)	3.0122(18)	O(2)-C(13)	1.428(3)
K(1)-O(4)	3.031(2)	O(2)-C(14)	1.429(3)
K(1)-C(4)	3.034(3)	O(3)-C(15)	1.422(3)
K(1)-C(3)	3.073(3)	O(3)-C(16)	1.431(3)
K(1)-C(5)	3.151(3)	O(4)-C(17)	1.414(4)
K(1)-C(2)	3.186(2)	O(4)-C(18)	1.430(3)
K(1)-C(1)	3.269(2)	O(5)-C(20)	1.422(3)
Si(1)-C(1)	1.848(3)	O(5)-C(19)	1.432(3)
Si(1)-C(8)	1.868(3)	O(6)-C(21)	1.416(3)
Si(1)-C(6)	1.880(3)	O(6)-C(22)	1.438(3)
Si(1)-C(7)	1.883(3)	C(1)-C(5)	1.417(4)
Si(2)-C(3)	1.855(3)	C(1)-C(2)	1.420(4)

C(2)-C(3)	1.419(4)	O(10)-C(41)	1.429(7)
C(3)-C(4)	1.425(4)	O(10)-C(41B)	1.517(8)
C(4)-C(5)	1.394(4)	O(11)-C(43)	1.374(5)
C(12)-C(13)	1.489(4)	O(11)-C(42)	1.452(6)
C(14)-C(15)	1.521(4)	O(11)-C(42B)	1.570(8)
C(16)-C(17)	1.499(4)	O(12)-C(45)	1.421(4)
C(18)-C(19)	1.492(4)	O(12)-C(44)	1.438(4)
C(20)-C(21)	1.506(4)	C(24)-C(25)	1.417(4)
C(22)-C(23)	1.487(4)	C(24)-C(28)	1.423(4)
K(2)-Cnt2	2.884	C(25)-C(26)	1.412(4)
K(2)-O(11)	2.810(2)	C(26)-C(27)	1.435(4)
K(2)-O(7)	2.820(2)	C(27)-C(28)	1.393(4)
K(2)-O(8)	2.868(2)	C(35)-C(36)	1.499(4)
K(2)-O(10)	2.903(2)	C(37)-C(38)	1.493(4)
K(2)-O(9)	2.9549(19)	C(39)-C(40)	1.486(4)
K(2)-O(12)	2.981(2)	C(41)-C(42)	1.510(9)
K(2)-C(24)	3.038(3)	C(41B)-C(42B)	1.455(10)
K(2)-C(28)	3.039(3)	C(43)-C(44)	1.470(6)
K(2)-C(25)	3.142(2)	C(45)-C(46)	1.506(5)
K(2)-C(27)	3.154(3)		
K(2)-C(26)	3.249(2)	Cnt1-K(1)-O(1)	141.6
Si(3)-C(24)	1.826(3)	Cnt1-K(1)-O(2)	107.0
Si(3)-C(29)	1.870(3)	Cnt1-K(1)-O(3)	107.9
Si(3)-C(30)	1.872(3)	Cnt1-K(1)-O(4)	113.4
Si(3)-C(31)	1.881(3)	Cnt1-K(1)-O(5)	101.3
Si(4)-C(26)	1.835(3)	Cnt1-K(1)-O(6)	108.0
Si(4)-C(32)	1.876(3)	O(3)-K(1)-O(2)	60.23(6)
Si(4)-C(33)	1.876(3)	O(3)-K(1)-O(6)	144.13(6)
Si(4)-C(34)	1.890(3)	O(2)-K(1)-O(6)	108.15(6)
O(7)-C(46)	1.416(4)	O(3)-K(1)-O(5)	113.78(6)
O(7)-C(35)	1.417(4)	O(2)-K(1)-O(5)	151.46(5)
O(8)-C(37)	1.426(3)	O(6)-K(1)-O(5)	58.69(6)
O(8)-C(36)	1.427(3)	O(3)-K(1)-O(1)	93.30(6)
O(9)-C(38)	1.419(3)	O(2)-K(1)-O(1)	56.40(5)
O(9)-C(39)	1.443(3)	O(6)-K(1)-O(1)	57.10(5)
O(10)-C(40)	1.422(4)	O(5)-K(1)-O(1)	98.53(5)

O(3)-K(1)-O(4)	57.74(6)	O(2)-K(1)-C(1)	101.82(6)
O(2)-K(1)-O(4)	113.06(6)	O(6)-K(1)-C(1)	128.94(6)
O(6)-K(1)-O(4)	107.09(6)	O(5)-K(1)-C(1)	105.73(6)
O(5)-K(1)-O(4)	56.23(6)	O(1)-K(1)-C(1)	153.46(6)
O(1)-K(1)-O(4)	104.98(5)	O(4)-K(1)-C(1)	97.46(6)
O(3)-K(1)-C(4)	129.04(7)	C(4)-K(1)-C(1)	42.50(7)
O(2)-K(1)-C(4)	122.98(7)	C(3)-K(1)-C(1)	43.19(7)
O(6)-K(1)-C(4)	86.45(6)	C(5)-K(1)-C(1)	25.43(7)
O(5)-K(1)-C(4)	83.39(7)	C(2)-K(1)-C(1)	25.37(7)
O(1)-K(1)-C(4)	133.02(7)	C(1)-Si(1)-C(8)	112.82(13)
O(4)-K(1)-C(4)	114.20(7)	C(1)-Si(1)-C(6)	110.65(14)
O(3)-K(1)-C(3)	121.26(7)	C(8)-Si(1)-C(6)	108.31(16)
O(2)-K(1)-C(3)	96.15(7)	C(1)-Si(1)-C(7)	111.77(13)
O(6)-K(1)-C(3)	92.53(6)	C(8)-Si(1)-C(7)	104.93(15)
O(5)-K(1)-C(3)	108.95(7)	C(6)-Si(1)-C(7)	108.07(13)
O(1)-K(1)-C(3)	118.25(6)	C(3)-Si(2)-C(9)	109.28(14)
O(4)-K(1)-C(3)	136.41(6)	C(3)-Si(2)-C(11)	112.24(13)
C(4)-K(1)-C(3)	26.97(7)	C(9)-Si(2)-C(11)	108.74(17)
O(3)-K(1)-C(5)	105.60(6)	C(3)-Si(2)-C(10)	112.99(13)
O(2)-K(1)-C(5)	125.93(6)	C(9)-Si(2)-C(10)	107.92(16)
O(6)-K(1)-C(5)	107.65(6)	C(11)-Si(2)-C(10)	105.48(15)
O(5)-K(1)-C(5)	82.46(6)	C(12)-O(1)-C(23)	112.1(2)
O(1)-K(1)-C(5)	158.96(6)	C(12)-O(1)-K(1)	115.48(14)
O(4)-K(1)-C(5)	93.11(6)	C(23)-O(1)-K(1)	116.11(15)
C(4)-K(1)-C(5)	25.97(7)	C(13)-O(2)-C(14)	113.0(2)
C(3)-K(1)-C(5)	43.40(7)	C(13)-O(2)-K(1)	120.00(15)
O(3)-K(1)-C(2)	95.33(7)	C(14)-O(2)-K(1)	106.44(15)
O(2)-K(1)-C(2)	85.48(6)	C(15)-O(3)-C(16)	112.2(2)
O(6)-K(1)-C(2)	118.54(6)	C(15)-O(3)-K(1)	121.14(16)
O(5)-K(1)-C(2)	122.97(6)	C(16)-O(3)-K(1)	122.78(16)
O(1)-K(1)-C(2)	128.84(6)	C(17)-O(4)-C(18)	111.1(2)
O(4)-K(1)-C(2)	122.04(6)	C(17)-O(4)-K(1)	105.57(15)
C(4)-K(1)-C(2)	42.38(7)	C(18)-O(4)-K(1)	106.08(16)
C(3)-K(1)-C(2)	26.12(7)	C(20)-O(5)-C(19)	113.5(2)
C(5)-K(1)-C(2)	41.65(7)	C(20)-O(5)-K(1)	120.24(16)
O(3)-K(1)-C(1)	86.75(7)	C(19)-O(5)-K(1)	122.48(16)

C(21)-O(6)-C(22)	113.8(2)	O(6)-C(22)-C(23)	108.2(2)
C(21)-O(6)-K(1)	109.56(15)	O(1)-C(23)-C(22)	107.3(2)
C(22)-O(6)-K(1)	111.09(15)	Cnt2-K(2)-O(7)	107.1
C(5)-C(1)-C(2)	105.2(2)	Cnt2-K(2)-O(8)	103.7
C(5)-C(1)-Si(1)	126.1(2)	Cnt2-K(2)-O(9)	138.6
C(2)-C(1)-Si(1)	128.1(2)	Cnt2-K(2)-O(10)	109.8
C(5)-C(1)-K(1)	72.64(13)	Cnt2-K(2)-O(11)	98.8
C(2)-C(1)-K(1)	74.07(13)	Cnt2-K(2)-O(12)	117.0
Si(1)-C(1)-K(1)	124.91(12)	O(11)-K(2)-O(7)	115.95(8)
C(3)-C(2)-C(1)	111.1(2)	O(11)-K(2)-O(8)	157.39(8)
C(3)-C(2)-K(1)	72.48(14)	O(7)-K(2)-O(8)	59.24(6)
C(1)-C(2)-K(1)	80.56(14)	O(11)-K(2)-O(10)	57.72(7)
C(2)-C(3)-C(4)	104.8(2)	O(7)-K(2)-O(10)	143.08(6)
C(2)-C(3)-Si(2)	128.0(2)	O(8)-K(2)-O(10)	111.21(6)
C(4)-C(3)-Si(2)	127.2(2)	O(11)-K(2)-O(9)	102.87(7)
C(2)-C(3)-K(1)	81.40(15)	O(7)-K(2)-O(9)	94.44(6)
C(4)-C(3)-K(1)	74.98(14)	O(8)-K(2)-O(9)	57.90(5)
Si(2)-C(3)-K(1)	108.01(10)	O(10)-K(2)-O(9)	57.12(6)
C(5)-C(4)-C(3)	109.5(2)	O(11)-K(2)-O(12)	57.67(8)
C(5)-C(4)-K(1)	81.71(15)	O(7)-K(2)-O(12)	58.32(7)
C(3)-C(4)-K(1)	78.05(15)	O(8)-K(2)-O(12)	112.29(6)
C(4)-C(5)-C(1)	109.4(2)	O(10)-K(2)-O(12)	103.00(7)
C(4)-C(5)-K(1)	72.33(14)	O(9)-K(2)-O(12)	104.37(6)
C(1)-C(5)-K(1)	81.94(14)	O(11)-K(2)-C(24)	110.65(9)
O(1)-C(12)-C(13)	108.7(2)	O(7)-K(2)-C(24)	116.28(7)
O(2)-C(13)-C(12)	108.3(2)	O(8)-K(2)-C(24)	89.70(7)
O(2)-C(14)-C(15)	113.1(2)	O(10)-K(2)-C(24)	98.22(7)
O(2)-C(14)-K(1)	50.76(12)	O(9)-K(2)-C(24)	114.98(7)
C(15)-C(14)-K(1)	85.19(16)	O(12)-K(2)-C(24)	140.65(6)
O(3)-C(15)-C(14)	107.7(2)	O(11)-K(2)-C(28)	83.80(9)
O(3)-C(16)-C(17)	108.0(2)	O(7)-K(2)-C(28)	129.89(7)
O(4)-C(17)-C(16)	109.3(2)	O(8)-K(2)-C(28)	116.76(7)
O(4)-C(18)-C(19)	108.7(2)	O(10)-K(2)-C(28)	86.91(7)
O(5)-C(19)-C(18)	108.5(2)	O(9)-K(2)-C(28)	127.25(7)
O(5)-C(20)-C(21)	108.9(2)	O(12)-K(2)-C(28)	121.81(7)
O(6)-C(21)-C(20)	113.9(2)	C(24)-K(2)-C(28)	27.08(7)

O(11)-K(2)-C(25)	120.18(8)	C(26)-Si(4)-C(34)	113.26(13)
O(7)-K(2)-C(25)	91.00(7)	C(32)-Si(4)-C(34)	104.78(15)
O(8)-K(2)-C(25)	82.43(6)	C(33)-Si(4)-C(34)	106.04(13)
O(10)-K(2)-C(25)	124.55(7)	C(46)-O(7)-C(35)	112.3(2)
O(9)-K(2)-C(25)	128.53(7)	C(46)-O(7)-K(2)	121.5(2)
O(12)-K(2)-C(25)	121.46(7)	C(35)-O(7)-K(2)	121.42(18)
C(24)-K(2)-C(25)	26.44(7)	C(37)-O(8)-C(36)	113.5(2)
C(28)-K(2)-C(25)	42.98(7)	C(37)-O(8)-K(2)	116.03(16)
O(11)-K(2)-C(27)	78.09(8)	C(36)-O(8)-K(2)	107.87(17)
O(7)-K(2)-C(27)	109.47(7)	C(38)-O(9)-C(39)	111.2(2)
O(8)-K(2)-C(27)	124.50(6)	C(38)-O(9)-K(2)	115.37(14)
O(10)-K(2)-C(27)	104.53(7)	C(39)-O(9)-K(2)	117.34(15)
O(9)-K(2)-C(27)	153.11(7)	C(40)-O(10)-C(41)	125.6(3)
O(12)-K(2)-C(27)	98.75(7)	C(40)-O(10)-C(41B)	97.1(4)
C(24)-K(2)-C(27)	43.39(7)	C(40)-O(10)-K(2)	115.55(17)
C(28)-K(2)-C(27)	25.92(7)	C(41)-O(10)-K(2)	106.4(3)
C(25)-K(2)-C(27)	42.13(7)	C(41B)-O(10)-K(2)	119.3(3)
O(11)-K(2)-C(26)	99.64(8)	C(43)-O(11)-C(42)	98.4(3)
O(7)-K(2)-C(26)	87.28(7)	C(43)-O(11)-C(42B)	129.1(4)
O(8)-K(2)-C(26)	102.07(6)	C(43)-O(11)-K(2)	122.6(2)
O(10)-K(2)-C(26)	128.89(7)	C(42)-O(11)-K(2)	122.7(3)
O(9)-K(2)-C(26)	153.97(6)	C(42B)-O(11)-K(2)	106.9(3)
O(12)-K(2)-C(26)	98.61(6)	C(45)-O(12)-C(44)	112.7(3)
C(24)-K(2)-C(26)	43.29(7)	C(45)-O(12)-K(2)	107.62(18)
C(28)-K(2)-C(26)	42.86(7)	C(44)-O(12)-K(2)	106.87(19)
C(25)-K(2)-C(26)	25.46(7)	C(25)-C(24)-C(28)	105.9(2)
C(27)-K(2)-C(26)	25.84(7)	C(25)-C(24)-Si(3)	127.3(2)
C(24)-Si(3)-C(29)	109.60(13)	C(28)-C(24)-Si(3)	126.8(2)
C(24)-Si(3)-C(30)	112.17(13)	C(25)-C(24)-K(2)	80.88(15)
C(29)-Si(3)-C(30)	106.46(15)	C(28)-C(24)-K(2)	76.48(15)
C(24)-Si(3)-C(31)	110.25(14)	Si(3)-C(24)-K(2)	110.86(10)
C(29)-Si(3)-C(31)	110.24(16)	C(26)-C(25)-C(24)	110.7(2)
C(30)-Si(3)-C(31)	108.05(15)	C(26)-C(25)-K(2)	81.49(15)
C(26)-Si(4)-C(32)	112.56(13)	C(24)-C(25)-K(2)	72.68(14)
C(26)-Si(4)-C(33)	109.32(14)	C(25)-C(26)-C(27)	105.3(2)
C(32)-Si(4)-C(33)	110.63(15)	C(25)-C(26)-Si(4)	128.1(2)

C(27)-C(26)-Si(4)	126.0(2)	O(8)-C(37)-C(38)	108.6(2)
C(25)-C(26)-K(2)	73.04(13)	O(9)-C(38)-C(37)	109.5(2)
C(27)-C(26)-K(2)	73.40(13)	O(9)-C(39)-C(40)	108.9(2)
Si(4)-C(26)-K(2)	125.37(12)	O(10)-C(40)-C(39)	108.8(2)
C(28)-C(27)-C(26)	109.2(2)	O(10)-C(41)-C(42)	108.7(5)
C(28)-C(27)-K(2)	72.39(14)	C(42B)-C(41B)-O(10)	102.3(6)
C(26)-C(27)-K(2)	80.76(14)	O(11)-C(42)-C(41)	102.2(4)
C(27)-C(28)-C(24)	108.9(2)	C(41B)-C(42B)-O(11)	104.5(6)
C(27)-C(28)-K(2)	81.69(16)	O(11)-C(43)-C(44)	108.1(3)
C(24)-C(28)-K(2)	76.44(15)	O(12)-C(44)-C(43)	110.5(3)
O(7)-C(35)-C(36)	108.5(2)	O(12)-C(45)-C(46)	110.1(3)
O(8)-C(36)-C(35)	113.2(2)	O(7)-C(46)-C(45)	108.6(3)

X-ray Data Collection, Structure Solution and Refinement for [Na(crown)$_2$][Cp″$_3$Th].

A red crystal of approximate dimensions 0.153 x 0.258 x 0.289 mm was mounted in a cryoloop and transferred to a Bruker SMART APEX II diffractometer. The APEX2[32] program package was used to determine the unit-cell parameters and for data collection (30 sec/frame scan time). The raw frame data was processed using SAINT[33] and SADABS[34] to yield the reflection data file. Subsequent calculations were carried out using the SHELXTL[35] program package. There were no systematic absences nor any diffraction symmetry other than the Friedel condition. The centrosymmetric triclinic space group $P\bar{1}$ was assigned and later determined to be correct.

The structure was solved by direct methods and refined on F^2 by full-matrix least-squares techniques. The analytical scattering factors[36] for neutral atoms were used throughout the analysis. Hydrogen atoms were included using a riding model. Disordered atoms were included using multiple components, partial site-occupancy-factors, thermal (EADP) and geometric restraints (DFIX).[35]

Least-squares analysis yielded wR2 = 0.1218 and Goof = 1.044 for 598 variables refined against 12873 data (0.80 Å), R1 = 0.0479 for those 10989 data with I > 2.0σ(I).

Table S4. Crystal data and structure refinement for [Na(crown)$_2$][Cp″$_3$Th].

Identification code	jcw35 (Justin Wedal)	
Empirical formula	$C_{57} H_{111} Na O_{12} Si_6 Th$	
Formula weight	1412.02	
Temperature	133(2) K	
Wavelength	0.71073 Å	
Crystal system	Triclinic	
Space group	$P\bar{1}$	
Unit cell dimensions	a = 13.8938(13) Å	α = 74.4602(14)°.
	b = 14.4588(13) Å	β = 84.1350(14)°.
	c = 18.3420(17) Å	γ = 83.5214(14)°.
Volume	3517.3(6) Å^3	
Z	2	
Density (calculated)	1.333 Mg/m^3	
Absorption coefficient	2.280 mm^{-1}	
F(000)	1468	
Crystal color	red	
Crystal size	0.289 x 0.258 x 0.153 mm^3	
Theta range for data collection	1.156 to 25.350°	
Index ranges	$-16 \leq h \leq 16$, $-17 \leq k \leq 17$, $-22 \leq l \leq 22$	
Reflections collected	39152	
Independent reflections	12873 [R(int) = 0.0464]	
Completeness to theta = 25.242°	100.0 %	
Absorption correction	Semi-empirical from equivalents	
Max. and min. transmission	0.6465 and 0.5424	
Refinement method	Full-matrix least-squares on F^2	
Data / restraints / parameters	12873 / 36 / 598	
Goodness-of-fit on F^2	1.044	
Final R indices [I>2sigma(I) = 10989 data]	R1 = 0.0479, wR2 = 0.1153	
R indices (all data, 0.80 Å)	R1 = 0.0608, wR2 = 0.1218	
Largest diff. peak and hole	2.464 and -2.311 e.Å^{-3}	

Table S5. Bond lengths [Å] and angles [°] for [Na(crown)$_2$][Cp″$_3$Th].

Th(1)-Cnt1	2.527	Th(1)-C(13)	2.754(6)
Th(1)-Cnt2	2.521	Th(1)-C(2)	2.757(6)
Th(1)-Cnt3	2.531	Th(1)-C(24)	2.761(6)

Th(1)-C(25)	2.785(6)	C(1)-C(2)	1.419(9)
Th(1)-C(12)	2.796(6)	C(1)-C(5)	1.431(9)
Th(1)-C(14)	2.798(6)	C(2)-C(3)	1.438(9)
Th(1)-C(3)	2.800(6)	C(3)-C(4)	1.434(9)
Th(1)-C(4)	2.807(6)	C(4)-C(5)	1.383(9)
Th(1)-C(16)	2.813(6)	C(12)-C(16)	1.423(9)
Th(1)-C(15)	2.816(6)	C(12)-C(13)	1.433(8)
Th(1)-C(1)	2.818(6)	C(13)-C(14)	1.426(9)
Th(1)-C(26)	2.818(6)	C(14)-C(15)	1.423(9)
Th(1)-C(23)	2.826(6)	C(15)-C(16)	1.390(9)
Th(1)-C(5)	2.826(6)	C(23)-C(24)	1.421(9)
Th(1)-C(27)	2.832(5)	C(23)-C(27)	1.426(8)
Si(1)-C(1)	1.849(7)	C(24)-C(25)	1.447(8)
Si(1)-C(7)	1.866(9)	C(25)-C(26)	1.424(8)
Si(1)-C(6)	1.881(8)	C(26)-C(27)	1.388(9)
Si(1)-C(8)	1.882(8)	Na(1)-O(12)	2.429(13)
Si(2)-C(3)	1.837(7)	Na(1)-O(6)	2.519(5)
Si(2)-C(11)	1.861(7)	Na(1)-O(2)	2.520(5)
Si(2)-C(9)	1.863(9)	Na(1)-O(7B)	2.541(19)
Si(2)-C(10)	1.877(8)	Na(1)-O(7)	2.579(13)
Si(3)-C(12)	1.847(7)	Na(1)-O(4)	2.583(5)
Si(3)-C(17)	1.865(9)	Na(1)-O(1)	2.589(5)
Si(3)-C(19)	1.866(8)	Na(1)-O(12B)	2.593(19)
Si(3)-C(18)	1.875(8)	Na(1)-O(3)	2.617(6)
Si(4)-C(14)	1.839(6)	Na(1)-O(5)	2.652(6)
Si(4)-C(20)	1.861(8)	O(1)-C(34)	1.414(8)
Si(4)-C(22)	1.879(7)	O(1)-C(45)	1.421(8)
Si(4)-C(21)	1.882(9)	O(2)-C(35)	1.410(8)
Si(5)-C(23)	1.846(6)	O(2)-C(36)	1.412(8)
Si(5)-C(28)	1.871(7)	O(3)-C(38)	1.344(11)
Si(5)-C(30)	1.872(7)	O(3)-C(37)	1.415(14)
Si(5)-C(29)	1.879(7)	O(4)-C(39)	1.403(11)
Si(6)-C(25)	1.848(6)	O(4)-C(40)	1.410(9)
Si(6)-C(33)	1.861(7)	O(5)-C(42)	1.398(9)
Si(6)-C(32)	1.869(7)	O(5)-C(41)	1.427(10)
Si(6)-C(31)	1.883(8)	O(6)-C(44)	1.414(8)

O(6)-C(43)	1.417(8)	O(12B)-C(55B)	1.417(10)
C(34)-C(35)	1.512(10)	C(46B)-C(47B)	1.494(10)
C(36)-C(37)	1.452(14)	C(48B)-C(49B)	1.490(10)
C(38)-C(39)	1.403(13)	C(50B)-C(51B)	1.505(10)
C(40)-C(41)	1.494(12)	C(52B)-C(53B)	1.496(10)
C(42)-C(43)	1.488(10)	C(54B)-C(55B)	1.474(10)
C(44)-C(45)	1.498(9)	C(56B)-C(57B)	1.515(10)
O(7)-C(57)	1.401(9)		
O(7)-C(46)	1.409(9)	Cnt1-Th(1)-Cnt2	120.0
O(8)-C(47)	1.417(9)	Cnt1-Th(1)-Cnt3	120.1
O(8)-C(48)	1.419(9)	Cnt2-Th(1)-Cnt3	119.9
O(9)-C(50)	1.405(9)	C(13)-Th(1)-C(2)	119.76(19)
O(9)-C(49)	1.417(9)	C(13)-Th(1)-C(24)	119.82(18)
O(10)-C(52)	1.403(9)	C(2)-Th(1)-C(24)	120.39(18)
O(10)-C(51)	1.403(9)	C(13)-Th(1)-C(25)	132.53(19)
O(11)-C(54)	1.375(9)	C(2)-Th(1)-C(25)	100.30(18)
O(11)-C(53)	1.402(9)	C(24)-Th(1)-C(25)	30.25(17)
O(12)-C(56)	1.407(9)	C(13)-Th(1)-C(12)	29.91(17)
O(12)-C(55)	1.419(9)	C(2)-Th(1)-C(12)	129.99(19)
C(46)-C(47)	1.492(9)	C(24)-Th(1)-C(12)	103.46(18)
C(48)-C(49)	1.438(9)	C(25)-Th(1)-C(12)	129.15(19)
C(50)-C(51)	1.470(9)	C(13)-Th(1)-C(14)	29.77(19)
C(52)-C(53)	1.470(9)	C(2)-Th(1)-C(14)	132.55(18)
C(54)-C(55)	1.466(9)	C(24)-Th(1)-C(14)	100.00(18)
C(56)-C(57)	1.500(9)	C(25)-Th(1)-C(14)	103.92(19)
O(7B)-C(57B)	1.410(10)	C(12)-Th(1)-C(14)	49.89(18)
O(7B)-C(46B)	1.422(10)	C(13)-Th(1)-C(3)	100.29(19)
O(8B)-C(47B)	1.436(10)	C(2)-Th(1)-C(3)	29.98(18)
O(8B)-C(48B)	1.445(9)	C(24)-Th(1)-C(3)	134.49(18)
O(9B)-C(50B)	1.441(10)	C(25)-Th(1)-C(3)	126.04(18)
O(9B)-C(49B)	1.457(9)	C(12)-Th(1)-C(3)	101.4(2)
O(10B)-C(52B)	1.412(9)	C(14)-Th(1)-C(3)	125.06(19)
O(10B)-C(51B)	1.433(9)	C(13)-Th(1)-C(4)	73.42(19)
O(11B)-C(54B)	1.357(9)	C(2)-Th(1)-C(4)	47.72(18)
O(11B)-C(53B)	1.419(9)	C(24)-Th(1)-C(4)	164.05(18)
O(12B)-C(56B)	1.416(10)	C(25)-Th(1)-C(4)	146.59(17)

C(12)-Th(1)-C(4)	84.08(19)	C(3)-Th(1)-C(26)	96.87(18)
C(14)-Th(1)-C(4)	95.60(19)	C(4)-Th(1)-C(26)	121.46(17)
C(3)-Th(1)-C(4)	29.63(18)	C(16)-Th(1)-C(26)	123.27(18)
C(13)-Th(1)-C(16)	47.83(18)	C(15)-Th(1)-C(26)	115.73(18)
C(2)-Th(1)-C(16)	158.3(2)	C(1)-Th(1)-C(26)	82.68(17)
C(24)-Th(1)-C(16)	75.38(18)	C(13)-Th(1)-C(23)	130.38(17)
C(25)-Th(1)-C(16)	99.76(18)	C(2)-Th(1)-C(23)	103.93(18)
C(12)-Th(1)-C(16)	29.39(19)	C(24)-Th(1)-C(23)	29.43(18)
C(14)-Th(1)-C(16)	48.49(17)	C(25)-Th(1)-C(23)	49.76(17)
C(3)-Th(1)-C(16)	128.39(19)	C(12)-Th(1)-C(23)	103.23(17)
C(4)-Th(1)-C(16)	113.43(19)	C(14)-Th(1)-C(23)	122.89(18)
C(13)-Th(1)-C(15)	47.7(2)	C(3)-Th(1)-C(23)	107.53(18)
C(2)-Th(1)-C(15)	161.57(18)	C(4)-Th(1)-C(23)	135.54(19)
C(24)-Th(1)-C(15)	73.37(18)	C(16)-Th(1)-C(23)	83.00(17)
C(25)-Th(1)-C(15)	86.64(18)	C(15)-Th(1)-C(23)	93.61(18)
C(12)-Th(1)-C(15)	48.4(2)	C(1)-Th(1)-C(23)	125.93(17)
C(14)-Th(1)-C(15)	29.36(18)	C(26)-Th(1)-C(23)	48.13(17)
C(3)-Th(1)-C(15)	147.32(19)	C(13)-Th(1)-C(5)	75.48(18)
C(4)-Th(1)-C(15)	120.96(19)	C(2)-Th(1)-C(5)	47.42(18)
C(16)-Th(1)-C(15)	28.59(18)	C(24)-Th(1)-C(5)	156.24(18)
C(13)-Th(1)-C(1)	103.48(18)	C(25)-Th(1)-C(5)	125.99(17)
C(2)-Th(1)-C(1)	29.48(18)	C(12)-Th(1)-C(5)	97.81(18)
C(24)-Th(1)-C(1)	128.61(18)	C(14)-Th(1)-C(5)	85.61(18)
C(25)-Th(1)-C(1)	99.60(18)	C(3)-Th(1)-C(5)	48.52(18)
C(12)-Th(1)-C(1)	127.16(18)	C(4)-Th(1)-C(5)	28.42(18)
C(14)-Th(1)-C(1)	105.69(18)	C(16)-Th(1)-C(5)	123.16(18)
C(3)-Th(1)-C(1)	49.97(19)	C(15)-Th(1)-C(5)	114.96(18)
C(4)-Th(1)-C(1)	48.42(19)	C(1)-Th(1)-C(5)	29.38(18)
C(16)-Th(1)-C(1)	151.05(18)	C(26)-Th(1)-C(5)	112.05(17)
C(15)-Th(1)-C(1)	132.80(18)	C(23)-Th(1)-C(5)	151.34(18)
C(13)-Th(1)-C(26)	161.63(19)	C(13)-Th(1)-C(27)	158.50(17)
C(2)-Th(1)-C(26)	73.76(18)	C(2)-Th(1)-C(27)	76.00(17)
C(24)-Th(1)-C(26)	47.90(17)	C(24)-Th(1)-C(27)	47.63(17)
C(25)-Th(1)-C(26)	29.44(17)	C(25)-Th(1)-C(27)	48.46(18)
C(12)-Th(1)-C(26)	150.04(18)	C(12)-Th(1)-C(27)	129.01(17)
C(14)-Th(1)-C(26)	132.04(19)	C(14)-Th(1)-C(27)	147.58(18)

C(3)-Th(1)-C(27)	87.30(18)	C(28)-Si(5)-C(29)	108.3(4)
C(4)-Th(1)-C(27)	116.82(18)	C(30)-Si(5)-C(29)	105.0(3)
C(16)-Th(1)-C(27)	112.13(17)	C(25)-Si(6)-C(33)	117.5(3)
C(15)-Th(1)-C(27)	120.21(17)	C(25)-Si(6)-C(32)	108.7(3)
C(1)-Th(1)-C(27)	96.81(17)	C(33)-Si(6)-C(32)	109.5(3)
C(26)-Th(1)-C(27)	28.43(17)	C(25)-Si(6)-C(31)	108.1(3)
C(23)-Th(1)-C(27)	29.19(16)	C(33)-Si(6)-C(31)	105.3(4)
C(5)-Th(1)-C(27)	123.00(17)	C(32)-Si(6)-C(31)	107.3(4)
C(1)-Si(1)-C(7)	111.8(3)	C(2)-C(1)-C(5)	104.0(5)
C(1)-Si(1)-C(6)	108.5(4)	C(2)-C(1)-Si(1)	126.1(5)
C(7)-Si(1)-C(6)	108.5(4)	C(5)-C(1)-Si(1)	127.1(5)
C(1)-Si(1)-C(8)	111.9(3)	C(2)-C(1)-Th(1)	72.9(3)
C(7)-Si(1)-C(8)	111.3(4)	C(5)-C(1)-Th(1)	75.6(3)
C(6)-Si(1)-C(8)	104.4(4)	Si(1)-C(1)-Th(1)	130.6(3)
C(3)-Si(2)-C(11)	110.4(3)	C(1)-C(2)-C(3)	112.3(5)
C(3)-Si(2)-C(9)	115.2(3)	C(1)-C(2)-Th(1)	77.6(3)
C(11)-Si(2)-C(9)	108.6(4)	C(3)-C(2)-Th(1)	76.7(3)
C(3)-Si(2)-C(10)	108.2(3)	C(4)-C(3)-C(2)	103.3(6)
C(11)-Si(2)-C(10)	105.8(4)	C(4)-C(3)-Si(2)	129.0(5)
C(9)-Si(2)-C(10)	108.2(5)	C(2)-C(3)-Si(2)	124.3(5)
C(12)-Si(3)-C(17)	107.8(4)	C(4)-C(3)-Th(1)	75.5(4)
C(12)-Si(3)-C(19)	112.1(3)	C(2)-C(3)-Th(1)	73.4(3)
C(17)-Si(3)-C(19)	106.2(4)	Si(2)-C(3)-Th(1)	131.4(3)
C(12)-Si(3)-C(18)	112.6(3)	C(5)-C(4)-C(3)	110.3(6)
C(17)-Si(3)-C(18)	106.9(4)	C(5)-C(4)-Th(1)	76.5(4)
C(19)-Si(3)-C(18)	110.9(4)	C(3)-C(4)-Th(1)	74.9(3)
C(14)-Si(4)-C(20)	112.7(3)	C(4)-C(5)-C(1)	110.1(6)
C(14)-Si(4)-C(22)	106.5(3)	C(4)-C(5)-Th(1)	75.0(3)
C(20)-Si(4)-C(22)	107.0(4)	C(1)-C(5)-Th(1)	75.0(3)
C(14)-Si(4)-C(21)	112.6(3)	C(16)-C(12)-C(13)	104.5(6)
C(20)-Si(4)-C(21)	110.6(5)	C(16)-C(12)-Si(3)	125.4(5)
C(22)-Si(4)-C(21)	106.9(4)	C(13)-C(12)-Si(3)	125.4(5)
C(23)-Si(5)-C(28)	110.9(3)	C(16)-C(12)-Th(1)	76.0(3)
C(23)-Si(5)-C(30)	113.2(3)	C(13)-C(12)-Th(1)	73.4(3)
C(28)-Si(5)-C(30)	111.0(3)	Si(3)-C(12)-Th(1)	134.4(3)
C(23)-Si(5)-C(29)	108.2(3)	C(14)-C(13)-C(12)	111.2(6)

C(14)-C(13)-Th(1)	76.8(3)	O(12)-Na(1)-O(2)	83.8(3)
C(12)-C(13)-Th(1)	76.7(3)	O(6)-Na(1)-O(2)	99.38(17)
C(15)-C(14)-C(13)	104.5(5)	O(6)-Na(1)-O(7B)	99.9(3)
C(15)-C(14)-Si(4)	125.9(5)	O(2)-Na(1)-O(7B)	135.8(4)
C(13)-C(14)-Si(4)	124.5(5)	O(12)-Na(1)-O(7)	65.6(3)
C(15)-C(14)-Th(1)	76.0(3)	O(6)-Na(1)-O(7)	92.7(3)
C(13)-C(14)-Th(1)	73.4(3)	O(2)-Na(1)-O(7)	135.1(3)
Si(4)-C(14)-Th(1)	135.0(3)	O(12)-Na(1)-O(4)	79.8(3)
C(16)-C(15)-C(14)	110.0(6)	O(6)-Na(1)-O(4)	123.22(19)
C(16)-C(15)-Th(1)	75.6(3)	O(2)-Na(1)-O(4)	118.0(2)
C(14)-C(15)-Th(1)	74.6(3)	O(7B)-Na(1)-O(4)	82.5(4)
C(15)-C(16)-C(12)	109.8(6)	O(7)-Na(1)-O(4)	89.2(3)
C(15)-C(16)-Th(1)	75.8(3)	O(12)-Na(1)-O(1)	90.4(3)
C(12)-C(16)-Th(1)	74.6(3)	O(6)-Na(1)-O(1)	64.70(16)
C(24)-C(23)-C(27)	105.1(5)	O(2)-Na(1)-O(1)	63.96(16)
C(24)-C(23)-Si(5)	125.2(4)	O(7B)-Na(1)-O(1)	89.6(4)
C(27)-C(23)-Si(5)	125.7(5)	O(7)-Na(1)-O(1)	83.4(3)
C(24)-C(23)-Th(1)	72.8(3)	O(4)-Na(1)-O(1)	169.57(19)
C(27)-C(23)-Th(1)	75.6(3)	O(6)-Na(1)-O(12B)	148.1(4)
Si(5)-C(23)-Th(1)	133.9(3)	O(2)-Na(1)-O(12B)	76.8(3)
C(23)-C(24)-C(25)	110.8(5)	O(7B)-Na(1)-O(12B)	66.0(5)
C(23)-C(24)-Th(1)	77.8(3)	O(4)-Na(1)-O(12B)	84.7(4)
C(25)-C(24)-Th(1)	75.8(3)	O(1)-Na(1)-O(12B)	85.9(4)
C(26)-C(25)-C(24)	104.2(5)	O(12)-Na(1)-O(3)	101.7(3)
C(26)-C(25)-Si(6)	129.3(4)	O(6)-Na(1)-O(3)	106.8(2)
C(24)-C(25)-Si(6)	122.2(4)	O(2)-Na(1)-O(3)	62.85(17)
C(26)-C(25)-Th(1)	76.6(3)	O(7B)-Na(1)-O(3)	144.0(4)
C(24)-C(25)-Th(1)	74.0(3)	O(7)-Na(1)-O(3)	151.6(3)
Si(6)-C(25)-Th(1)	132.2(3)	O(4)-Na(1)-O(3)	62.88(18)
C(27)-C(26)-C(25)	110.1(5)	O(1)-Na(1)-O(3)	123.42(18)
C(27)-C(26)-Th(1)	76.3(3)	O(12B)-Na(1)-O(3)	99.5(4)
C(25)-C(26)-Th(1)	74.0(3)	O(12)-Na(1)-O(5)	124.0(3)
C(26)-C(27)-C(23)	109.8(5)	O(6)-Na(1)-O(5)	62.89(16)
C(26)-C(27)-Th(1)	75.2(3)	O(2)-Na(1)-O(5)	150.22(18)
C(23)-C(27)-Th(1)	75.2(3)	O(7B)-Na(1)-O(5)	73.3(4)
O(12)-Na(1)-O(6)	149.4(3)	O(7)-Na(1)-O(5)	72.5(3)

O(4)-Na(1)-O(5)	63.88(18)	C(46)-O(7)-Na(1)	125.6(10)
O(1)-Na(1)-O(5)	120.20(18)	C(47)-O(8)-C(48)	107.4(10)
O(12B)-Na(1)-O(5)	131.2(4)	C(50)-O(9)-C(49)	111.3(10)
O(3)-Na(1)-O(5)	97.9(2)	C(52)-O(10)-C(51)	116.8(10)
C(34)-O(1)-C(45)	113.1(5)	C(54)-O(11)-C(53)	132.5(10)
C(34)-O(1)-Na(1)	106.6(4)	C(56)-O(12)-C(55)	111.4(11)
C(45)-O(1)-Na(1)	109.7(4)	C(56)-O(12)-Na(1)	114.9(9)
C(35)-O(2)-C(36)	114.6(5)	C(55)-O(12)-Na(1)	115.2(9)
C(35)-O(2)-Na(1)	121.8(4)	O(7)-C(46)-C(47)	114.2(12)
C(36)-O(2)-Na(1)	120.6(4)	O(8)-C(47)-C(46)	109.0(11)
C(38)-O(3)-C(37)	106.2(9)	O(8)-C(48)-C(49)	106.0(11)
C(38)-O(3)-Na(1)	116.0(5)	O(9)-C(49)-C(48)	128.5(11)
C(37)-O(3)-Na(1)	118.4(7)	O(9)-C(50)-C(51)	113.1(10)
C(39)-O(4)-C(40)	112.1(7)	O(10)-C(51)-C(50)	105.8(9)
C(39)-O(4)-Na(1)	113.9(5)	O(10)-C(52)-C(53)	126.0(11)
C(40)-O(4)-Na(1)	110.4(5)	O(11)-C(53)-C(52)	117.0(11)
C(42)-O(5)-C(41)	113.7(6)	O(11)-C(54)-C(55)	119.8(11)
C(42)-O(5)-Na(1)	110.3(4)	O(12)-C(55)-C(54)	120.9(12)
C(41)-O(5)-Na(1)	117.7(5)	O(12)-C(56)-C(57)	107.9(13)
C(44)-O(6)-C(43)	112.1(5)	O(7)-C(57)-C(56)	109.0(12)
C(44)-O(6)-Na(1)	118.0(4)	C(57B)-O(7B)-C(46B)	113.3(17)
C(43)-O(6)-Na(1)	121.3(4)	C(57B)-O(7B)-Na(1)	115.3(15)
O(1)-C(34)-C(35)	113.1(6)	C(46B)-O(7B)-Na(1)	123.4(14)
O(2)-C(35)-C(34)	107.6(6)	C(47B)-O(8B)-C(48B)	120.4(15)
O(2)-C(36)-C(37)	112.3(8)	C(50B)-O(9B)-C(49B)	104.8(13)
O(3)-C(37)-C(36)	111.2(10)	C(52B)-O(10B)-C(51B)	137.1(15)
O(3)-C(38)-C(39)	112.5(9)	C(54B)-O(11B)-C(53B)	111.5(15)
O(4)-C(39)-C(38)	111.8(9)	C(56B)-O(12B)-C(55B)	108.2(16)
O(4)-C(40)-C(41)	113.0(7)	C(56B)-O(12B)-Na(1)	107.4(14)
O(5)-C(41)-C(40)	108.2(7)	C(55B)-O(12B)-Na(1)	115.5(14)
O(5)-C(42)-C(43)	108.9(6)	O(7B)-C(46B)-C(47B)	113.3(17)
O(6)-C(43)-C(42)	108.6(6)	O(8B)-C(47B)-C(46B)	109.7(16)
O(6)-C(44)-C(45)	108.8(6)	O(8B)-C(48B)-C(49B)	123.3(17)
O(1)-C(45)-C(44)	107.2(5)	O(9B)-C(49B)-C(48B)	119.1(16)
C(57)-O(7)-C(46)	116.2(12)	O(9B)-C(50B)-C(51B)	104.4(12)
C(57)-O(7)-Na(1)	114.2(10)	O(10B)-C(51B)-C(50B)	109.7(13)

O(10B)-C(52B)-C(53B)	112.3(16)	O(12B)-C(55B)-C(54B)	113.4(17)
O(11B)-C(53B)-C(52B)	113.7(15)	O(12B)-C(56B)-C(57B)	108(2)
O(11B)-C(54B)-C(55B)	118.6(16)	O(7B)-C(57B)-C(56B)	108.2(18)

X-ray Data Collection, Structure Solution and Refinement for [Rb(crypt)][Cp″$_3$Th].

A blue crystal of approximate dimensions 0.296 x 0.316 x 0.435 mm was mounted in a cryoloop and transferred to a Bruker SMART APEX II diffractometer. The APEX2[32] program package was used to determine the unit-cell parameters and for data collection (10 sec/frame scan time). The raw frame data was processed using SAINT[33] and SADABS[34] to yield the reflection data file. Subsequent calculations were carried out using the SHELXTL[35] program package. There were no systematic absences nor any diffraction symmetry other than the Friedel condition. The centrosymmetric triclinic space group $P\bar{1}$ was assigned and later determined to be correct.

The structure was solved by direct methods and refined on F^2 by full-matrix least-squares techniques. The analytical scattering factors[36] for neutral atoms were used throughout the analysis. Hydrogen atoms were included using a riding model.

Least-squares analysis yielded wR2 = 0.0475 and Goof = 1.019 for 622 variables refined against 15844 data (0.73 Å), R1 = 0.0212 for those 14524 data with I > 2.0σ(I).

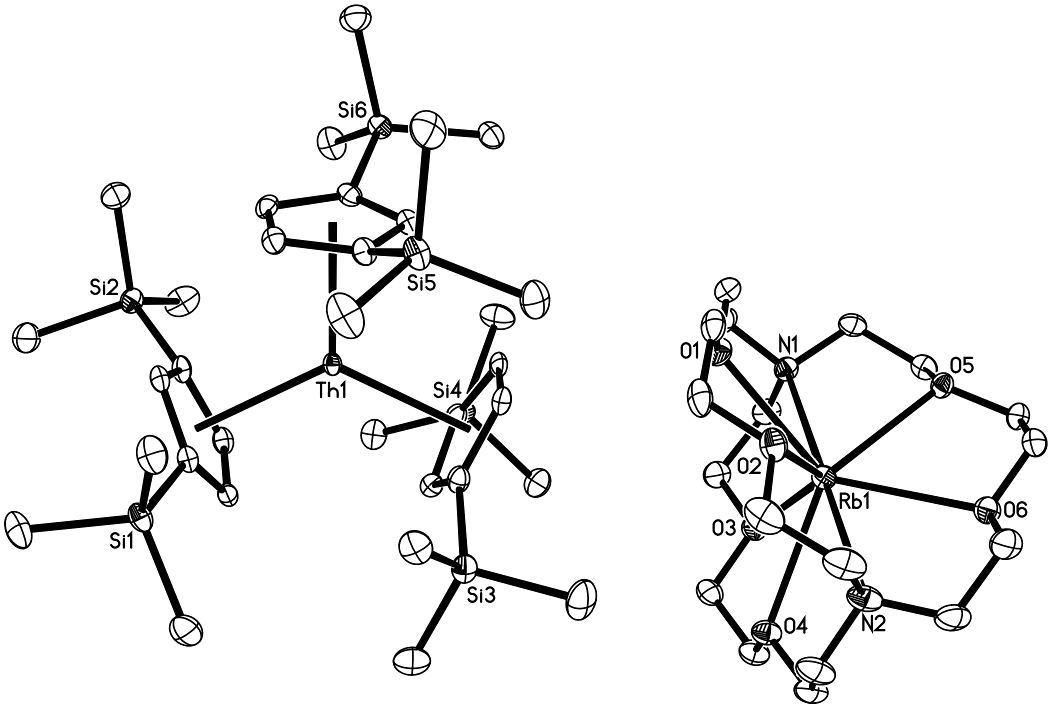

Figure S69: Thermal ellipsoid plot of [Rb(crypt)][Cp″$_3$Th^{II}] drawn at the 50% probability level. Hydrogen atoms have been removed for clarity.

Table S6. Crystal data and structure refinement for [Rb(crypt)][Cp″$_3$Th].

Identification code	nrr6 (Nick Rightmire)	
Empirical formula	$C_{51} H_{99} N_2 O_6 Rb Si_6 Th$	
Formula weight	1322.37	
Temperature	88(2) K	
Wavelength	0.71073 Å	
Crystal system	Triclinic	
Space group	$P\bar{1}$	
Unit cell dimensions	a = 12.1971(13) Å	α = 100.6648(13)°.
	b = 12.7473(13) Å	β = 104.4725(13)°.
	c = 22.242(2) Å	γ = 95.7340(13)°.
Volume	3251.6(6) Å^3	
Z	2	

Density (calculated)	1.351 Mg/m^3
Absorption coefficient	3.189 mm^{-1}
F(000)	1356
Crystal color	blue
Crystal size	0.435 x 0.316 x 0.296 mm^3
Theta range for data collection	1.645 to 29.044°
Index ranges	$-16 \leq h \leq 16$, $-17 \leq k \leq 17$, $-30 \leq l \leq 30$
Reflections collected	40057
Independent reflections	15844 [R(int) = 0.0213]
Completeness to theta = 25.242°	99.9 %
Absorption correction	Numerical
Max. and min. transmission	0.4085 and 0.2845
Refinement method	Full-matrix least-squares on F^2
Data / restraints / parameters	15844 / 0 / 622
Goodness-of-fit on F^2	1.019
Final R indices [I>2sigma(I) = 14524 data]	R1 = 0.0212, wR2 = 0.0462
R indices (all data, ? Å)	R1 = 0.0256, wR2 = 0.0475
Largest diff. peak and hole	1.117 and -0.690 e.$Å^{-3}$

Table S7. Bond lengths [Å] and angles [°] for [Rb(crypt)][Cp″$_3$Th].

Th(1)-Cnt1	2.536	Th(1)-C(27)	2.853(2)
Th(1)-Cnt2	2.509	Th(1)-C(4)	2.8567(19)
Th(1)-Cnt3	2.522	Th(1)-C(23)	2.860(2)
Th(1)-C(13)	2.739(2)	Si(1)-C(1)	1.848(2)
Th(1)-C(25)	2.7453(19)	Si(1)-C(8)	1.868(2)
Th(1)-C(24)	2.7556(19)	Si(1)-C(7)	1.873(2)
Th(1)-C(2)	2.7607(19)	Si(1)-C(6)	1.886(2)
Th(1)-C(12)	2.770(2)	Si(2)-C(3)	1.849(2)
Th(1)-C(1)	2.7774(19)	Si(2)-C(9)	1.868(2)
Th(1)-C(26)	2.7825(19)	Si(2)-C(11)	1.877(2)
Th(1)-C(16)	2.8029(19)	Si(2)-C(10)	1.885(2)
Th(1)-C(14)	2.8087(19)	Si(3)-C(12)	1.842(2)
Th(1)-C(5)	2.8139(19)	Si(3)-C(19)	1.873(2)
Th(1)-C(15)	2.8208(19)	Si(3)-C(18)	1.874(2)
Th(1)-C(3)	2.8502(19)	Si(3)-C(17)	1.880(2)

Si(4)-C(14)	1.850(2)	O(1)-C(36)	1.434(3)
Si(4)-C(20)	1.869(2)	O(2)-C(38)	1.425(3)
Si(4)-C(21)	1.870(2)	O(2)-C(37)	1.428(3)
Si(4)-C(22)	1.880(2)	O(3)-C(41)	1.421(3)
Si(5)-C(23)	1.852(2)	O(3)-C(42)	1.430(3)
Si(5)-C(30)	1.867(2)	O(4)-C(43)	1.423(3)
Si(5)-C(29)	1.870(2)	O(4)-C(44)	1.430(3)
Si(5)-C(28)	1.882(2)	O(5)-C(47)	1.427(2)
Si(6)-C(25)	1.848(2)	O(5)-C(48)	1.428(2)
Si(6)-C(32)	1.869(2)	O(6)-C(50)	1.423(3)
Si(6)-C(33)	1.875(2)	O(6)-C(49)	1.432(3)
Si(6)-C(31)	1.878(2)	N(1)-C(34)	1.469(3)
C(1)-C(5)	1.439(3)	N(1)-C(40)	1.473(3)
C(1)-C(2)	1.440(3)	N(1)-C(46)	1.479(3)
C(2)-C(3)	1.432(3)	N(2)-C(51)	1.468(3)
C(3)-C(4)	1.426(3)	N(2)-C(39)	1.475(3)
C(4)-C(5)	1.401(3)	N(2)-C(45)	1.480(3)
C(12)-C(16)	1.437(3)	C(34)-C(35)	1.505(3)
C(12)-C(13)	1.447(3)	C(36)-C(37)	1.496(3)
C(13)-C(14)	1.434(3)	C(38)-C(39)	1.490(4)
C(14)-C(15)	1.430(3)	C(40)-C(41)	1.511(3)
C(15)-C(16)	1.401(3)	C(42)-C(43)	1.506(3)
C(23)-C(27)	1.426(3)	C(44)-C(45)	1.506(3)
C(23)-C(24)	1.431(3)	C(46)-C(47)	1.505(3)
C(24)-C(25)	1.439(3)	C(48)-C(49)	1.497(3)
C(25)-C(26)	1.443(3)	C(50)-C(51)	1.514(3)
C(26)-C(27)	1.401(3)		
Rb(1)-O(1)	2.8662(15)	Cnt1-Th(1)-Cnt2	122.7
Rb(1)-O(2)	2.8717(15)	Cnt1-Th(1)-Cnt3	119.7
Rb(1)-O(5)	2.8743(14)	Cnt2-Th(1)-Cnt3	117.6
Rb(1)-O(6)	2.8822(15)	C(13)-Th(1)-C(25)	132.68(6)
Rb(1)-O(3)	2.8890(15)	C(13)-Th(1)-C(24)	118.07(6)
Rb(1)-O(4)	2.8918(15)	C(25)-Th(1)-C(24)	30.32(6)
Rb(1)-N(1)	3.0535(17)	C(13)-Th(1)-C(2)	121.78(6)
Rb(1)-N(2)	3.0564(19)	C(25)-Th(1)-C(2)	100.74(6)
O(1)-C(35)	1.424(3)	C(24)-Th(1)-C(2)	120.05(6)

C(13)-Th(1)-C(12)	30.46(6)
C(25)-Th(1)-C(12)	125.95(6)
C(24)-Th(1)-C(12)	99.49(6)
C(2)-Th(1)-C(12)	131.61(6)
C(13)-Th(1)-C(1)	98.38(6)
C(25)-Th(1)-C(1)	128.26(6)
C(24)-Th(1)-C(1)	136.88(6)
C(2)-Th(1)-C(1)	30.13(6)
C(12)-Th(1)-C(1)	101.64(6)
C(13)-Th(1)-C(26)	162.92(6)
C(25)-Th(1)-C(26)	30.24(6)
C(24)-Th(1)-C(26)	48.36(6)
C(2)-Th(1)-C(26)	73.21(6)
C(12)-Th(1)-C(26)	145.89(6)
C(1)-Th(1)-C(26)	98.43(6)
C(13)-Th(1)-C(16)	48.53(6)
C(25)-Th(1)-C(16)	96.17(6)
C(24)-Th(1)-C(16)	71.67(6)
C(2)-Th(1)-C(16)	159.86(6)
C(12)-Th(1)-C(16)	29.87(6)
C(1)-Th(1)-C(16)	129.99(6)
C(26)-Th(1)-C(16)	120.00(6)
C(13)-Th(1)-C(14)	29.92(6)
C(25)-Th(1)-C(14)	104.82(6)
C(24)-Th(1)-C(14)	100.12(6)
C(2)-Th(1)-C(14)	134.53(6)
C(12)-Th(1)-C(14)	50.36(6)
C(1)-Th(1)-C(14)	122.26(6)
C(26)-Th(1)-C(14)	134.01(6)
C(16)-Th(1)-C(14)	48.79(6)
C(13)-Th(1)-C(5)	73.75(6)
C(25)-Th(1)-C(5)	145.41(6)
C(24)-Th(1)-C(5)	166.59(6)
C(2)-Th(1)-C(5)	48.14(6)
C(12)-Th(1)-C(5)	88.29(6)
C(1)-Th(1)-C(5)	29.82(6)
C(26)-Th(1)-C(5)	121.20(6)
C(16)-Th(1)-C(5)	117.63(6)
C(14)-Th(1)-C(5)	93.26(6)
C(13)-Th(1)-C(15)	48.20(6)
C(25)-Th(1)-C(15)	85.26(6)
C(24)-Th(1)-C(15)	72.33(6)
C(2)-Th(1)-C(15)	163.26(6)
C(12)-Th(1)-C(15)	49.05(6)
C(1)-Th(1)-C(15)	146.42(6)
C(26)-Th(1)-C(15)	115.13(6)
C(16)-Th(1)-C(15)	28.86(6)
C(14)-Th(1)-C(15)	29.42(6)
C(5)-Th(1)-C(15)	120.65(6)
C(13)-Th(1)-C(3)	109.19(6)
C(25)-Th(1)-C(3)	97.55(6)
C(24)-Th(1)-C(3)	126.30(6)
C(2)-Th(1)-C(3)	29.52(5)
C(12)-Th(1)-C(3)	133.89(6)
C(1)-Th(1)-C(3)	49.73(6)
C(26)-Th(1)-C(3)	79.79(6)
C(16)-Th(1)-C(3)	156.80(6)
C(14)-Th(1)-C(3)	109.28(6)
C(5)-Th(1)-C(3)	48.17(6)
C(15)-Th(1)-C(3)	134.68(6)
C(13)-Th(1)-C(27)	154.35(6)
C(25)-Th(1)-C(27)	49.12(6)
C(24)-Th(1)-C(27)	47.64(6)
C(2)-Th(1)-C(27)	75.44(6)
C(12)-Th(1)-C(27)	124.05(6)
C(1)-Th(1)-C(27)	89.64(6)
C(26)-Th(1)-C(27)	28.76(6)
C(16)-Th(1)-C(27)	108.51(6)
C(14)-Th(1)-C(27)	147.75(6)
C(5)-Th(1)-C(27)	118.97(6)
C(15)-Th(1)-C(27)	119.18(6)
C(3)-Th(1)-C(27)	94.61(6)

C(13)-Th(1)-C(4)	80.63(6)	C(3)-Si(2)-C(10)	106.88(10)
C(25)-Th(1)-C(4)	122.02(6)	C(9)-Si(2)-C(10)	108.62(12)
C(24)-Th(1)-C(4)	152.34(6)	C(11)-Si(2)-C(10)	103.03(11)
C(2)-Th(1)-C(4)	47.63(6)	C(12)-Si(3)-C(19)	107.34(10)
C(12)-Th(1)-C(4)	105.39(6)	C(12)-Si(3)-C(18)	114.96(10)
C(1)-Th(1)-C(4)	48.65(6)	C(19)-Si(3)-C(18)	111.38(10)
C(26)-Th(1)-C(4)	108.58(6)	C(12)-Si(3)-C(17)	109.78(10)
C(16)-Th(1)-C(4)	129.09(6)	C(19)-Si(3)-C(17)	106.66(12)
C(14)-Th(1)-C(4)	86.90(6)	C(18)-Si(3)-C(17)	106.43(11)
C(5)-Th(1)-C(4)	28.60(6)	C(14)-Si(4)-C(20)	110.80(10)
C(15)-Th(1)-C(4)	116.04(6)	C(14)-Si(4)-C(21)	114.44(9)
C(3)-Th(1)-C(4)	28.93(6)	C(20)-Si(4)-C(21)	108.68(11)
C(27)-Th(1)-C(4)	121.70(6)	C(14)-Si(4)-C(22)	107.51(9)
C(13)-Th(1)-C(23)	127.02(6)	C(20)-Si(4)-C(22)	107.34(11)
C(25)-Th(1)-C(23)	50.02(6)	C(21)-Si(4)-C(22)	107.80(10)
C(24)-Th(1)-C(23)	29.44(5)	C(23)-Si(5)-C(30)	112.94(10)
C(2)-Th(1)-C(23)	103.04(6)	C(23)-Si(5)-C(29)	113.14(10)
C(12)-Th(1)-C(23)	98.57(6)	C(30)-Si(5)-C(29)	110.06(11)
C(1)-Th(1)-C(23)	109.80(6)	C(23)-Si(5)-C(28)	106.83(10)
C(26)-Th(1)-C(23)	48.32(6)	C(30)-Si(5)-C(28)	105.97(11)
C(16)-Th(1)-C(23)	79.64(6)	C(29)-Si(5)-C(28)	107.44(11)
C(14)-Th(1)-C(23)	122.22(6)	C(25)-Si(6)-C(32)	117.87(10)
C(5)-Th(1)-C(23)	138.83(6)	C(25)-Si(6)-C(33)	109.48(10)
C(15)-Th(1)-C(23)	92.81(6)	C(32)-Si(6)-C(33)	106.61(10)
C(3)-Th(1)-C(23)	123.39(6)	C(25)-Si(6)-C(31)	106.99(10)
C(27)-Th(1)-C(23)	28.91(5)	C(32)-Si(6)-C(31)	107.11(11)
C(4)-Th(1)-C(23)	150.35(6)	C(33)-Si(6)-C(31)	108.47(10)
C(1)-Si(1)-C(8)	110.90(10)	C(5)-C(1)-C(2)	104.38(17)
C(1)-Si(1)-C(7)	112.46(10)	C(5)-C(1)-Si(1)	127.10(15)
C(8)-Si(1)-C(7)	112.23(11)	C(2)-C(1)-Si(1)	124.23(15)
C(1)-Si(1)-C(6)	108.11(10)	C(5)-C(1)-Th(1)	76.50(11)
C(8)-Si(1)-C(6)	106.35(11)	C(2)-C(1)-Th(1)	74.29(10)
C(7)-Si(1)-C(6)	106.42(10)	Si(1)-C(1)-Th(1)	132.21(9)
C(3)-Si(2)-C(9)	111.74(10)	C(3)-C(2)-C(1)	111.03(17)
C(3)-Si(2)-C(11)	114.91(9)	C(3)-C(2)-Th(1)	78.71(11)
C(9)-Si(2)-C(11)	111.04(10)	C(1)-C(2)-Th(1)	75.58(11)

C(4)-C(3)-C(2)	105.17(17)
C(4)-C(3)-Si(2)	126.10(15)
C(2)-C(3)-Si(2)	124.74(15)
C(4)-C(3)-Th(1)	75.79(11)
C(2)-C(3)-Th(1)	71.77(10)
Si(2)-C(3)-Th(1)	134.76(9)
C(5)-C(4)-C(3)	109.74(17)
C(5)-C(4)-Th(1)	74.01(11)
C(3)-C(4)-Th(1)	75.28(11)
C(4)-C(5)-C(1)	109.69(17)
C(4)-C(5)-Th(1)	77.40(11)
C(1)-C(5)-Th(1)	73.69(11)
C(16)-C(12)-C(13)	104.37(16)
C(16)-C(12)-Si(3)	127.27(15)
C(13)-C(12)-Si(3)	126.14(15)
C(16)-C(12)-Th(1)	76.32(11)
C(13)-C(12)-Th(1)	73.56(11)
Si(3)-C(12)-Th(1)	127.91(9)
C(14)-C(13)-C(12)	110.98(17)
C(14)-C(13)-Th(1)	77.75(11)
C(12)-C(13)-Th(1)	75.98(11)
C(15)-C(14)-C(13)	104.98(17)
C(15)-C(14)-Si(4)	125.52(15)
C(13)-C(14)-Si(4)	126.21(15)
C(15)-C(14)-Th(1)	75.76(11)
C(13)-C(14)-Th(1)	72.34(11)
Si(4)-C(14)-Th(1)	132.60(9)
C(16)-C(15)-C(14)	109.90(17)
C(16)-C(15)-Th(1)	74.86(11)
C(14)-C(15)-Th(1)	74.82(11)
C(15)-C(16)-C(12)	109.77(17)
C(15)-C(16)-Th(1)	76.28(11)
C(12)-C(16)-Th(1)	73.82(11)
C(27)-C(23)-C(24)	105.04(17)
C(27)-C(23)-Si(5)	126.28(15)
C(24)-C(23)-Si(5)	123.24(14)
C(27)-C(23)-Th(1)	75.26(11)
C(24)-C(23)-Th(1)	71.24(11)
Si(5)-C(23)-Th(1)	138.55(10)
C(23)-C(24)-C(25)	111.52(17)
C(23)-C(24)-Th(1)	79.32(11)
C(25)-C(24)-Th(1)	74.44(11)
C(24)-C(25)-C(26)	103.87(17)
C(24)-C(25)-Si(6)	122.62(14)
C(26)-C(25)-Si(6)	129.28(15)
C(24)-C(25)-Th(1)	75.24(11)
C(26)-C(25)-Th(1)	76.30(11)
Si(6)-C(25)-Th(1)	131.11(9)
C(27)-C(26)-C(25)	109.94(17)
C(27)-C(26)-Th(1)	78.40(11)
C(25)-C(26)-Th(1)	73.45(11)
C(26)-C(27)-C(23)	109.63(17)
C(26)-C(27)-Th(1)	72.84(11)
C(23)-C(27)-Th(1)	75.83(11)
O(1)-Rb(1)-O(2)	61.10(4)
O(1)-Rb(1)-O(5)	95.33(4)
O(2)-Rb(1)-O(5)	117.99(4)
O(1)-Rb(1)-O(6)	135.12(4)
O(2)-Rb(1)-O(6)	95.55(4)
O(5)-Rb(1)-O(6)	60.48(4)
O(1)-Rb(1)-O(3)	96.57(4)
O(2)-Rb(1)-O(3)	136.43(4)
O(5)-Rb(1)-O(3)	99.82(4)
O(6)-Rb(1)-O(3)	122.98(4)
O(1)-Rb(1)-O(4)	120.16(4)
O(2)-Rb(1)-O(4)	97.57(4)
O(5)-Rb(1)-O(4)	139.35(4)
O(6)-Rb(1)-O(4)	99.32(4)
O(3)-Rb(1)-O(4)	59.79(4)
O(1)-Rb(1)-N(1)	59.84(5)
O(2)-Rb(1)-N(1)	120.20(5)
O(5)-Rb(1)-N(1)	60.40(4)

O(6)-Rb(1)-N(1)	120.02(5)	C(39)-N(2)-C(45)	109.86(19)
O(3)-Rb(1)-N(1)	59.96(4)	C(51)-N(2)-Rb(1)	108.43(13)
O(4)-Rb(1)-N(1)	119.09(4)	C(39)-N(2)-Rb(1)	108.67(13)
O(1)-Rb(1)-N(2)	120.45(5)	C(45)-N(2)-Rb(1)	108.40(13)
O(2)-Rb(1)-N(2)	60.17(5)	N(1)-C(34)-C(35)	113.95(18)
O(5)-Rb(1)-N(2)	119.91(5)	O(1)-C(35)-C(34)	108.94(18)
O(6)-Rb(1)-N(2)	60.19(5)	O(1)-C(35)-Rb(1)	44.03(9)
O(3)-Rb(1)-N(2)	119.48(5)	C(34)-C(35)-Rb(1)	81.67(12)
O(4)-Rb(1)-N(2)	60.36(5)	O(1)-C(36)-C(37)	108.68(18)
N(1)-Rb(1)-N(2)	179.43(5)	O(1)-C(36)-Rb(1)	46.58(9)
C(35)-O(1)-C(36)	112.31(17)	C(37)-C(36)-Rb(1)	76.80(12)
C(35)-O(1)-Rb(1)	115.76(12)	O(2)-C(37)-C(36)	109.94(17)
C(36)-O(1)-Rb(1)	112.11(12)	O(2)-C(37)-Rb(1)	48.15(9)
C(38)-O(2)-C(37)	111.11(17)	C(36)-C(37)-Rb(1)	79.47(12)
C(38)-O(2)-Rb(1)	114.71(13)	O(2)-C(38)-C(39)	109.77(19)
C(37)-O(2)-Rb(1)	110.12(12)	O(2)-C(38)-Rb(1)	44.81(10)
C(41)-O(3)-C(42)	111.21(16)	C(39)-C(38)-Rb(1)	82.10(13)
C(41)-O(3)-Rb(1)	115.91(12)	N(2)-C(39)-C(38)	114.5(2)
C(42)-O(3)-Rb(1)	112.69(12)	N(1)-C(40)-C(41)	114.17(18)
C(43)-O(4)-C(44)	111.87(17)	O(3)-C(41)-C(40)	109.26(17)
C(43)-O(4)-Rb(1)	113.56(12)	O(3)-C(41)-Rb(1)	44.07(9)
C(44)-O(4)-Rb(1)	114.19(12)	C(40)-C(41)-Rb(1)	80.80(11)
C(47)-O(5)-C(48)	111.66(16)	O(3)-C(42)-C(43)	109.13(17)
C(47)-O(5)-Rb(1)	112.71(11)	O(3)-C(42)-Rb(1)	46.33(9)
C(48)-O(5)-Rb(1)	113.69(11)	C(43)-C(42)-Rb(1)	78.73(12)
C(50)-O(6)-C(49)	111.15(17)	O(4)-C(43)-C(42)	109.05(18)
C(50)-O(6)-Rb(1)	115.59(13)	O(4)-C(43)-Rb(1)	45.79(9)
C(49)-O(6)-Rb(1)	110.58(12)	C(42)-C(43)-Rb(1)	77.73(12)
C(34)-N(1)-C(40)	110.42(17)	O(4)-C(44)-C(45)	108.91(19)
C(34)-N(1)-C(46)	110.20(17)	O(4)-C(44)-Rb(1)	45.25(9)
C(40)-N(1)-C(46)	109.25(17)	C(45)-C(44)-Rb(1)	81.38(12)
C(34)-N(1)-Rb(1)	109.23(12)	N(2)-C(45)-C(44)	114.33(19)
C(40)-N(1)-Rb(1)	109.09(12)	N(1)-C(46)-C(47)	113.85(17)
C(46)-N(1)-Rb(1)	108.61(12)	O(5)-C(47)-C(46)	108.76(16)
C(51)-N(2)-C(39)	110.90(19)	O(5)-C(47)-Rb(1)	46.26(9)
C(51)-N(2)-C(45)	110.51(19)	C(46)-C(47)-Rb(1)	83.05(11)

O(5)-C(48)-C(49)	109.25(18)	C(48)-C(49)-Rb(1)	79.96(12)
O(5)-C(48)-Rb(1)	45.54(9)	O(6)-C(50)-C(51)	109.36(18)
C(49)-C(48)-Rb(1)	76.49(12)	O(6)-C(50)-Rb(1)	44.25(10)
O(6)-C(49)-C(48)	109.76(18)	C(51)-C(50)-Rb(1)	80.64(12)
O(6)-C(49)-Rb(1)	47.82(9)	N(2)-C(51)-C(50)	113.90(19)

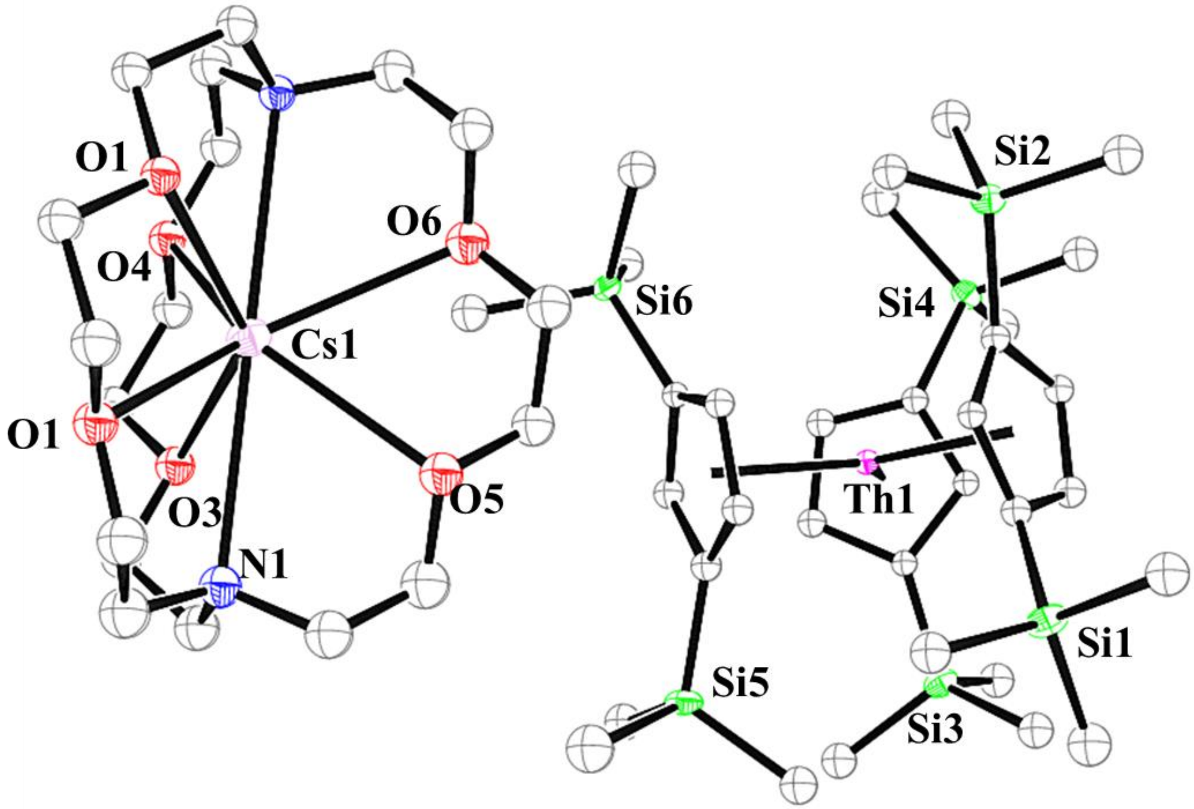

Figure S70: Thermal ellipsoid plot of [Cs(crypt)][Cp″$_3$Th^{II}] plotted at the 50% probability level. Hydrogen atoms have been removed for clarity.

X-ray Data Collection, Structure Solution and Refinement for [Cs(crypt)][Cp″$_3$Th].

A blue crystal of approximate dimensions 0.082 x 0.110 x 0.182 mm was mounted in a cryoloop and transferred to a Bruker SMART APEX II diffractometer. The APEX2[32] program package was used to determine the unit-cell parameters and for data collection (120 sec/frame scan time). The raw frame data was processed using SAINT[33] and SADABS[34] to yield the reflection

data file. Subsequent calculations were carried out using the SHELXTL[35] program package. There were no systematic absences nor any diffraction symmetry other than the Friedel condition. The centrosymmetric triclinic space group $P\bar{1}$ was assigned and later determined to be correct.

The structure was solved by direct methods and refined on F^2 by full-matrix least-squares techniques. The analytical scattering factors[36] for neutral atoms were used throughout the analysis. Hydrogen atoms were included using a riding model.

Least-squares analysis yielded wR2 = 0.0866 and Goof = 1.024 for 622 variables refined against 16125 data (0.75 Å), R1 = 0.0382 for those 13147 data with I > 2.0σ(I).

Table S. Crystal data and structure refinement for [Cs(crypt)][Cp″$_3$Th].

Identification code	jcw48 (Justin Wedal)	
Empirical formula	$C_{51} H_{99} Cs N_2 O_6 Si_6 Th$	
Formula weight	1369.81	
Temperature	133(2) K	
Wavelength	0.71073 Å	
Crystal system	Triclinic	
Space group	$P\bar{1}$	
Unit cell dimensions	a = 12.1953(8) Å	α = 100.6711(10)°.
	b = 12.7501(8) Å	β = 104.3758(9)°.
	c = 22.2212(14) Å	γ = 95.7421(10)°.
Volume	3250.2(4) Å^3	
Z	2	
Density (calculated)	1.400 Mg/m^3	
Absorption coefficient	2.998 mm^{-1}	
F(000)	1392	
Crystal color	blue	
Crystal size	0.182 x 0.110 x 0.082 mm^3	
Theta range for data collection	1.645 to 28.322°	
Index ranges	$-16 \leq h \leq 16$, $-17 \leq k \leq 17$, $-29 \leq l \leq 29$	
Reflections collected	43962	
Independent reflections	16125 [R(int) = 0.0464]	
Completeness to theta = 25.242°	100.0 %	

Absorption correction	Semi-empirical from equivalents
Max. and min. transmission	0.6471 and 0.5186
Refinement method	Full-matrix least-squares on F^2
Data / restraints / parameters	16125 / 0 / 622
Goodness-of-fit on F^2	1.024
Final R indices [I>2sigma(I) = 13147 data]	R1 = 0.0382, wR2 = 0.0809
R indices (all data, 0.75 Å)	R1 = 0.0556, wR2 = 0.0866
Largest diff. peak and hole	1.613 and -2.543 e.Å^{-3}

Table S9. Bond lengths [Å] and angles [°] for [Cs(crypt)][Cp″$_3$Th].

Th(1)-Cnt1	2.534	Si(2)-C(11)	1.880(5)
Th(1)-Cnt2	2.522	Si(3)-C(12)	1.846(4)
Th(1)-Cnt3	2.507	Si(3)-C(19)	1.867(5)
Th(1)-C(24)	2.731(4)	Si(3)-C(17)	1.873(5)
Th(1)-C(12)	2.749(4)	Si(3)-C(18)	1.876(5)
Th(1)-C(13)	2.756(4)	Si(4)-C(14)	1.851(4)
Th(1)-C(2)	2.759(4)	Si(4)-C(22)	1.866(5)
Th(1)-C(25)	2.769(4)	Si(4)-C(20)	1.868(5)
Th(1)-C(3)	2.776(4)	Si(4)-C(21)	1.887(5)
Th(1)-C(16)	2.782(4)	Si(5)-C(23)	1.847(4)
Th(1)-C(26)	2.795(4)	Si(5)-C(28)	1.853(5)
Th(1)-C(23)	2.804(4)	Si(5)-C(29)	1.862(5)
Th(1)-C(4)	2.807(4)	Si(5)-C(30)	1.883(5)
Th(1)-C(27)	2.816(4)	Si(6)-C(25)	1.837(5)
Th(1)-C(15)	2.843(4)	Si(6)-C(33)	1.862(5)
Th(1)-C(1)	2.848(4)	Si(6)-C(32)	1.865(5)
Th(1)-C(5)	2.853(4)	Si(6)-C(31)	1.870(5)
Th(1)-C(14)	2.854(4)	C(1)-C(5)	1.424(6)
Si(1)-C(1)	1.844(4)	C(1)-C(2)	1.425(6)
Si(1)-C(8)	1.867(5)	C(2)-C(3)	1.438(6)
Si(1)-C(7)	1.872(5)	C(3)-C(4)	1.439(6)
Si(1)-C(6)	1.887(5)	C(4)-C(5)	1.398(6)
Si(2)-C(3)	1.843(4)	C(12)-C(16)	1.438(6)
Si(2)-C(9)	1.860(5)	C(12)-C(13)	1.440(6)
Si(2)-C(10)	1.866(5)	C(13)-C(14)	1.421(6)

C(14)-C(15)	1.423(6)	C(40)-C(41)	1.508(7)
C(15)-C(16)	1.389(6)	C(42)-C(43)	1.501(7)
C(23)-C(27)	1.418(6)	C(44)-C(45)	1.502(7)
C(23)-C(24)	1.432(6)	C(46)-C(47)	1.508(7)
C(24)-C(25)	1.435(6)	C(48)-C(49)	1.490(8)
C(25)-C(26)	1.433(6)	C(50)-C(51)	1.497(8)
C(26)-C(27)	1.392(6)		
Cs(1)-O(6)	2.931(3)	Cnt1-Th(1)-Cnt2	119.7
Cs(1)-O(5)	2.933(3)	Cnt1-Th(1)-Cnt3	122.7
Cs(1)-O(1)	2.933(3)	Cnt2-Th(1)-Cnt3	117.6
Cs(1)-O(2)	2.941(3)	C(24)-Th(1)-C(12)	132.66(12)
Cs(1)-O(3)	2.942(3)	C(24)-Th(1)-C(13)	117.94(12)
Cs(1)-O(4)	2.947(3)	C(12)-Th(1)-C(13)	30.33(12)
Cs(1)-N(2)	3.073(4)	C(24)-Th(1)-C(2)	121.81(13)
Cs(1)-N(1)	3.073(4)	C(12)-Th(1)-C(2)	100.86(13)
O(1)-C(35)	1.414(5)	C(13)-Th(1)-C(2)	120.14(13)
O(1)-C(36)	1.426(5)	C(24)-Th(1)-C(25)	30.24(13)
O(2)-C(37)	1.426(6)	C(12)-Th(1)-C(25)	126.02(13)
O(2)-C(38)	1.429(6)	C(13)-Th(1)-C(25)	99.55(13)
O(3)-C(42)	1.420(5)	C(2)-Th(1)-C(25)	131.41(13)
O(3)-C(41)	1.421(6)	C(24)-Th(1)-C(3)	98.37(12)
O(4)-C(44)	1.418(6)	C(12)-Th(1)-C(3)	128.34(13)
O(4)-C(43)	1.427(6)	C(13)-Th(1)-C(3)	136.89(12)
O(5)-C(47)	1.421(6)	C(2)-Th(1)-C(3)	30.11(13)
O(5)-C(48)	1.431(6)	C(25)-Th(1)-C(3)	101.48(13)
O(6)-C(49)	1.425(6)	C(24)-Th(1)-C(16)	162.80(12)
O(6)-C(50)	1.426(6)	C(12)-Th(1)-C(16)	30.15(12)
N(1)-C(34)	1.469(6)	C(13)-Th(1)-C(16)	48.30(12)
N(1)-C(46)	1.471(6)	C(2)-Th(1)-C(16)	73.40(12)
N(1)-C(40)	1.474(6)	C(25)-Th(1)-C(16)	145.92(13)
N(2)-C(39)	1.473(7)	C(3)-Th(1)-C(16)	98.59(12)
N(2)-C(51)	1.475(7)	C(24)-Th(1)-C(26)	48.13(13)
N(2)-C(45)	1.476(6)	C(12)-Th(1)-C(26)	96.27(13)
C(34)-C(35)	1.515(7)	C(13)-Th(1)-C(26)	71.84(13)
C(36)-C(37)	1.501(7)	C(2)-Th(1)-C(26)	159.78(13)
C(38)-C(39)	1.514(7)	C(25)-Th(1)-C(26)	29.85(12)

C(3)-Th(1)-C(26)	129.90(13)	C(26)-Th(1)-C(15)	108.78(12)
C(16)-Th(1)-C(26)	120.12(12)	C(23)-Th(1)-C(15)	147.57(12)
C(24)-Th(1)-C(23)	29.96(12)	C(4)-Th(1)-C(15)	119.20(12)
C(12)-Th(1)-C(23)	104.84(12)	C(27)-Th(1)-C(15)	119.24(12)
C(13)-Th(1)-C(23)	100.12(12)	C(24)-Th(1)-C(1)	109.50(12)
C(2)-Th(1)-C(23)	134.52(12)	C(12)-Th(1)-C(1)	97.55(12)
C(25)-Th(1)-C(23)	50.35(12)	C(13)-Th(1)-C(1)	126.27(12)
C(3)-Th(1)-C(23)	122.23(12)	C(2)-Th(1)-C(1)	29.39(12)
C(16)-Th(1)-C(23)	133.93(12)	C(25)-Th(1)-C(1)	133.90(13)
C(26)-Th(1)-C(23)	48.49(12)	C(3)-Th(1)-C(1)	49.70(12)
C(24)-Th(1)-C(4)	73.85(12)	C(16)-Th(1)-C(1)	79.73(12)
C(12)-Th(1)-C(4)	145.38(12)	C(26)-Th(1)-C(1)	156.69(12)
C(13)-Th(1)-C(4)	166.64(12)	C(23)-Th(1)-C(1)	109.48(12)
C(2)-Th(1)-C(4)	48.05(12)	C(4)-Th(1)-C(1)	48.11(12)
C(25)-Th(1)-C(4)	88.25(13)	C(27)-Th(1)-C(1)	134.72(12)
C(3)-Th(1)-C(4)	29.87(12)	C(15)-Th(1)-C(1)	94.44(12)
C(16)-Th(1)-C(4)	121.28(12)	C(24)-Th(1)-C(5)	80.91(12)
C(26)-Th(1)-C(4)	117.51(13)	C(12)-Th(1)-C(5)	121.91(12)
C(23)-Th(1)-C(4)	93.21(12)	C(13)-Th(1)-C(5)	152.24(13)
C(24)-Th(1)-C(27)	47.97(12)	C(2)-Th(1)-C(5)	47.54(12)
C(12)-Th(1)-C(27)	85.41(12)	C(25)-Th(1)-C(5)	105.45(13)
C(13)-Th(1)-C(27)	72.52(12)	C(3)-Th(1)-C(5)	48.73(12)
C(2)-Th(1)-C(27)	163.09(12)	C(16)-Th(1)-C(5)	108.50(12)
C(25)-Th(1)-C(27)	49.06(12)	C(26)-Th(1)-C(5)	128.95(12)
C(3)-Th(1)-C(27)	146.21(12)	C(23)-Th(1)-C(5)	86.98(12)
C(16)-Th(1)-C(27)	115.19(12)	C(4)-Th(1)-C(5)	28.59(12)
C(26)-Th(1)-C(27)	28.73(12)	C(27)-Th(1)-C(5)	115.91(12)
C(23)-Th(1)-C(27)	29.22(12)	C(15)-Th(1)-C(5)	121.64(12)
C(4)-Th(1)-C(27)	120.43(12)	C(1)-Th(1)-C(5)	28.93(12)
C(24)-Th(1)-C(15)	154.28(13)	C(24)-Th(1)-C(14)	126.84(13)
C(12)-Th(1)-C(15)	48.83(12)	C(12)-Th(1)-C(14)	49.87(12)
C(13)-Th(1)-C(15)	47.46(12)	C(13)-Th(1)-C(14)	29.28(12)
C(2)-Th(1)-C(15)	75.59(12)	C(2)-Th(1)-C(14)	103.15(12)
C(25)-Th(1)-C(15)	124.23(13)	C(25)-Th(1)-C(14)	98.67(13)
C(3)-Th(1)-C(15)	89.87(12)	C(3)-Th(1)-C(14)	109.92(12)
C(16)-Th(1)-C(15)	28.57(12)	C(16)-Th(1)-C(14)	48.25(13)

C(26)-Th(1)-C(14)	79.88(12)	C(29)-Si(5)-C(30)	106.8(2)
C(23)-Th(1)-C(14)	122.13(12)	C(25)-Si(6)-C(33)	115.2(2)
C(4)-Th(1)-C(14)	139.06(12)	C(25)-Si(6)-C(32)	107.3(2)
C(27)-Th(1)-C(14)	92.92(12)	C(33)-Si(6)-C(32)	111.0(2)
C(15)-Th(1)-C(14)	28.92(12)	C(25)-Si(6)-C(31)	109.9(2)
C(1)-Th(1)-C(14)	123.25(12)	C(33)-Si(6)-C(31)	106.4(2)
C(5)-Th(1)-C(14)	150.32(12)	C(32)-Si(6)-C(31)	106.7(3)
C(1)-Si(1)-C(8)	115.2(2)	C(5)-C(1)-C(2)	105.2(4)
C(1)-Si(1)-C(7)	111.6(2)	C(5)-C(1)-Si(1)	125.8(3)
C(8)-Si(1)-C(7)	110.8(2)	C(2)-C(1)-Si(1)	124.9(3)
C(1)-Si(1)-C(6)	106.6(2)	C(5)-C(1)-Th(1)	75.7(2)
C(8)-Si(1)-C(6)	102.7(2)	C(2)-C(1)-Th(1)	71.8(2)
C(7)-Si(1)-C(6)	109.3(2)	Si(1)-C(1)-Th(1)	134.8(2)
C(3)-Si(2)-C(9)	112.4(2)	C(1)-C(2)-C(3)	111.4(4)
C(3)-Si(2)-C(10)	110.8(2)	C(1)-C(2)-Th(1)	78.8(2)
C(9)-Si(2)-C(10)	112.7(2)	C(3)-C(2)-Th(1)	75.6(2)
C(3)-Si(2)-C(11)	108.2(2)	C(2)-C(3)-C(4)	104.0(4)
C(9)-Si(2)-C(11)	105.8(2)	C(2)-C(3)-Si(2)	124.2(3)
C(10)-Si(2)-C(11)	106.6(2)	C(4)-C(3)-Si(2)	127.4(3)
C(12)-Si(3)-C(19)	117.8(2)	C(2)-C(3)-Th(1)	74.3(2)
C(12)-Si(3)-C(17)	106.6(2)	C(4)-C(3)-Th(1)	76.3(2)
C(19)-Si(3)-C(17)	107.2(2)	Si(2)-C(3)-Th(1)	132.5(2)
C(12)-Si(3)-C(18)	109.5(2)	C(5)-C(4)-C(3)	109.9(4)
C(19)-Si(3)-C(18)	106.8(2)	C(5)-C(4)-Th(1)	77.5(2)
C(17)-Si(3)-C(18)	108.7(2)	C(3)-C(4)-Th(1)	73.9(2)
C(14)-Si(4)-C(22)	113.0(2)	C(4)-C(5)-C(1)	109.5(4)
C(14)-Si(4)-C(20)	113.2(2)	C(4)-C(5)-Th(1)	73.9(2)
C(22)-Si(4)-C(20)	110.0(3)	C(1)-C(5)-Th(1)	75.3(2)
C(14)-Si(4)-C(21)	106.7(2)	C(16)-C(12)-C(13)	103.8(4)
C(22)-Si(4)-C(21)	106.0(2)	C(16)-C(12)-Si(3)	129.6(3)
C(20)-Si(4)-C(21)	107.5(3)	C(13)-C(12)-Si(3)	122.6(3)
C(23)-Si(5)-C(28)	114.9(2)	C(16)-C(12)-Th(1)	76.2(2)
C(23)-Si(5)-C(29)	110.7(2)	C(13)-C(12)-Th(1)	75.1(2)
C(28)-Si(5)-C(29)	109.1(2)	Si(3)-C(12)-Th(1)	130.75(19)
C(23)-Si(5)-C(30)	107.5(2)	C(14)-C(13)-C(12)	111.4(4)
C(28)-Si(5)-C(30)	107.4(2)	C(14)-C(13)-Th(1)	79.2(2)

C(12)-C(13)-Th(1)	74.5(2)	O(5)-Cs(1)-O(1)	95.39(9)
C(13)-C(14)-C(15)	104.9(4)	O(6)-Cs(1)-O(2)	95.79(9)
C(13)-C(14)-Si(4)	123.3(3)	O(5)-Cs(1)-O(2)	135.79(9)
C(15)-C(14)-Si(4)	126.2(3)	O(1)-Cs(1)-O(2)	60.38(9)
C(13)-C(14)-Th(1)	71.6(2)	O(6)-Cs(1)-O(3)	137.09(9)
C(15)-C(14)-Th(1)	75.1(2)	O(5)-Cs(1)-O(3)	96.65(9)
Si(4)-C(14)-Th(1)	138.6(2)	O(1)-Cs(1)-O(3)	100.03(9)
C(16)-C(15)-C(14)	110.1(4)	O(2)-Cs(1)-O(3)	122.23(9)
C(16)-C(15)-Th(1)	73.3(2)	O(6)-Cs(1)-O(4)	97.59(9)
C(14)-C(15)-Th(1)	76.0(2)	O(5)-Cs(1)-O(4)	119.05(9)
C(15)-C(16)-C(12)	109.8(4)	O(1)-Cs(1)-O(4)	140.46(9)
C(15)-C(16)-Th(1)	78.2(2)	O(2)-Cs(1)-O(4)	99.77(9)
C(12)-C(16)-Th(1)	73.7(2)	O(3)-Cs(1)-O(4)	59.79(9)
C(27)-C(23)-C(24)	104.7(4)	O(6)-Cs(1)-N(2)	60.24(11)
C(27)-C(23)-Si(5)	125.6(3)	O(5)-Cs(1)-N(2)	120.16(11)
C(24)-C(23)-Si(5)	126.4(3)	O(1)-Cs(1)-N(2)	119.84(10)
C(27)-C(23)-Th(1)	75.9(2)	O(2)-Cs(1)-N(2)	60.42(11)
C(24)-C(23)-Th(1)	72.2(2)	O(3)-Cs(1)-N(2)	119.56(10)
Si(5)-C(23)-Th(1)	132.6(2)	O(4)-Cs(1)-N(2)	60.60(10)
C(23)-C(24)-C(25)	111.6(4)	O(6)-Cs(1)-N(1)	119.77(10)
C(23)-C(24)-Th(1)	77.8(2)	O(5)-Cs(1)-N(1)	59.81(10)
C(25)-C(24)-Th(1)	76.3(2)	O(1)-Cs(1)-N(1)	60.35(9)
C(26)-C(25)-C(24)	103.6(4)	O(2)-Cs(1)-N(1)	119.75(10)
C(26)-C(25)-Si(6)	127.4(3)	O(3)-Cs(1)-N(1)	60.28(9)
C(24)-C(25)-Si(6)	126.8(3)	O(4)-Cs(1)-N(1)	119.22(9)
C(26)-C(25)-Th(1)	76.1(2)	N(2)-Cs(1)-N(1)	179.80(11)
C(24)-C(25)-Th(1)	73.4(2)	C(35)-O(1)-C(36)	112.5(3)
Si(6)-C(25)-Th(1)	127.8(2)	C(35)-O(1)-Cs(1)	110.0(2)
C(27)-C(26)-C(25)	110.3(4)	C(36)-O(1)-Cs(1)	111.6(3)
C(27)-C(26)-Th(1)	76.5(2)	C(37)-O(2)-C(38)	111.7(4)
C(25)-C(26)-Th(1)	74.1(2)	C(37)-O(2)-Cs(1)	108.7(3)
C(26)-C(27)-C(23)	109.8(4)	C(38)-O(2)-Cs(1)	112.3(3)
C(26)-C(27)-Th(1)	74.8(2)	C(42)-O(3)-C(41)	112.3(4)
C(23)-C(27)-Th(1)	74.9(2)	C(42)-O(3)-Cs(1)	110.8(3)
O(6)-Cs(1)-O(5)	60.88(10)	C(41)-O(3)-Cs(1)	112.9(3)
O(6)-Cs(1)-O(1)	117.03(9)	C(44)-O(4)-C(43)	112.8(4)

C(44)-O(4)-Cs(1)	110.5(3)	N(2)-C(39)-Cs(1)	50.1(2)
C(43)-O(4)-Cs(1)	111.3(3)	C(38)-C(39)-Cs(1)	75.5(3)
C(47)-O(5)-C(48)	113.1(4)	N(1)-C(40)-C(41)	114.8(4)
C(47)-O(5)-Cs(1)	113.1(3)	N(1)-C(40)-Cs(1)	50.1(2)
C(48)-O(5)-Cs(1)	110.0(3)	C(41)-C(40)-Cs(1)	75.8(3)
C(49)-O(6)-C(50)	111.6(4)	O(3)-C(41)-C(40)	109.9(4)
C(49)-O(6)-Cs(1)	108.2(3)	O(3)-C(41)-Cs(1)	46.6(2)
C(50)-O(6)-Cs(1)	112.0(3)	C(40)-C(41)-Cs(1)	81.1(3)
C(34)-N(1)-C(46)	110.3(4)	O(3)-C(42)-C(43)	109.7(4)
C(34)-N(1)-C(40)	110.0(4)	O(3)-C(42)-Cs(1)	48.2(2)
C(46)-N(1)-C(40)	110.5(4)	C(43)-C(42)-Cs(1)	79.0(2)
C(34)-N(1)-Cs(1)	108.5(3)	O(4)-C(43)-C(42)	109.3(4)
C(46)-N(1)-Cs(1)	109.1(3)	O(4)-C(43)-Cs(1)	47.7(2)
C(40)-N(1)-Cs(1)	108.3(3)	C(42)-C(43)-Cs(1)	77.6(2)
C(39)-N(2)-C(51)	111.0(4)	O(4)-C(44)-C(45)	109.7(4)
C(39)-N(2)-C(45)	110.6(4)	O(4)-C(44)-Cs(1)	48.4(2)
C(51)-N(2)-C(45)	110.6(4)	C(45)-C(44)-Cs(1)	82.2(3)
C(39)-N(2)-Cs(1)	108.3(3)	N(2)-C(45)-C(44)	114.9(4)
C(51)-N(2)-Cs(1)	108.6(3)	N(2)-C(45)-Cs(1)	50.6(2)
C(45)-N(2)-Cs(1)	107.6(3)	C(44)-C(45)-Cs(1)	74.7(3)
N(1)-C(34)-C(35)	114.0(4)	N(1)-C(46)-C(47)	114.2(4)
N(1)-C(34)-Cs(1)	50.0(2)	N(1)-C(46)-Cs(1)	49.6(2)
C(35)-C(34)-Cs(1)	73.3(2)	C(47)-C(46)-Cs(1)	75.1(3)
O(1)-C(35)-C(34)	109.5(4)	O(5)-C(47)-C(46)	109.3(4)
O(1)-C(35)-Cs(1)	48.72(19)	O(5)-C(47)-Cs(1)	46.4(2)
C(34)-C(35)-Cs(1)	83.4(3)	C(46)-C(47)-Cs(1)	81.8(3)
O(1)-C(36)-C(37)	109.6(4)	O(5)-C(48)-C(49)	109.1(4)
O(1)-C(36)-Cs(1)	47.4(2)	O(5)-C(48)-Cs(1)	48.6(2)
C(37)-C(36)-Cs(1)	76.6(3)	C(49)-C(48)-Cs(1)	76.8(3)
O(2)-C(37)-C(36)	109.9(4)	O(6)-C(49)-C(48)	110.5(4)
O(2)-C(37)-Cs(1)	49.6(2)	O(6)-C(49)-Cs(1)	50.0(2)
C(36)-C(37)-Cs(1)	79.9(3)	C(48)-C(49)-Cs(1)	79.7(3)
O(2)-C(38)-C(39)	110.0(4)	O(6)-C(50)-C(51)	110.0(4)
O(2)-C(38)-Cs(1)	46.9(2)	O(6)-C(50)-Cs(1)	47.2(2)
C(39)-C(38)-Cs(1)	81.3(3)	C(51)-C(50)-Cs(1)	82.3(3)
N(2)-C(39)-C(38)	113.8(4)	N(2)-C(51)-C(50)	114.8(4)

N(2)-C(51)-Cs(1)	49.9(2)
C(50)-C(51)-Cs(1)	74.7(3)

References

(1) MacDonald, M. R.; Fieser, M. E.; Bates, J. E.; Ziller, J. W.; Furche, F.; Evans, W. J. Identification of the +2 Oxidation State for Uranium in a Crystalline Molecular Complex, [K(2.2.2-Cryptand)][$(C_5H_4SiMe_3)_3U$]. *J. Am. Chem. Soc.* **2013**, *135*, 13310–13313, DOI: 10.1021/ja406791t.

(2) Windorff, C. J.; MacDonald, M. R.; Meihaus, K. R.; Ziller, J. W.; Long, J. R.; Evans, W. J. Expanding the Chemistry of Molecular U^{2+} Complexes: Synthesis, Characterization, and Reactivity of the $\{[C_5H_3(SiMe_3)_2]_3U\}^-$ Anion. *Chem. Eur. J.* **2016**, *22*, 772–782, DOI: 10.1002/chem.201503583.

(3) Evans, W. J.; Kozimor, S. A.; Ziller, J. W.; Fagin, A. A.; Bochkarev, M. N. Facile Syntheses of Unsolvated UI_3 and Tetramethylcyclopentadienyl Uranium Halides. *Inorg. Chem.* **2005**, *44*, 3993–4000, DOI: 10.1021/ic0482685.

(4) Andersen, R. A. Tris ((Hexamethyldisilyl) Amido) Uranium (III): Preparation and Coordination Chemistry. *Inorg. Chem.* **1979**, *18*, 1507–1509, DOI: 10.1021/ic50196a021.

(5) Blake, P. C.; Edelstein, N. M.; Hitchcock, P. B.; Kot, W. K.; Lappert, M. F.; Shalimoff, G. V.; Tian, S. Synthesis, Properties and Structures of the Tris(Cyclopentadienyl)Thorium(III) Complexes [Th{η^5–$C_5H_3(SiMe_2R)_2$-1,3}$_3$] (R=Me or ^{t}Bu). *J. Organomet. Chem.* **2001**, *636*, 124–129, DOI: 10.1016/S0022-328X(01)00860-9.

(6) Weydert, M.; Brennan, J. G.; Andersen, R. A.; Bergman, R. G. Reactions of Uranium(IV)

Tertiary Alkyl Bond: Facile Ligand-Assisted Reduction and Insertion of Ethylene and Carbon Monoxide. *Organometallics* **1995**, *14*, 3942–3951, DOI: 10.1021/om00008a046.

(7) Siladke, N. A.; Webster, C. L.; Walensky, J. R.; Takase, M. K.; Ziller, J. W.; Grant, D. J.; Gagliardi, L.; Evans, W. J. Actinide Metallocene Hydride Chemistry: C–H Activation in Tetramethylcyclopentadienyl Ligands to Form [μ–η^5–$C_5Me_3H(CH_2)$–κC]$^{2-}$ Tuck-over Ligands in a Tetrathorium Octahydride Complex. *Organometallics* **2013**, *32*, 6522–6531, DOI: 10.1021/om4008482.

(8) Blake, P. C.; Lappert, M. F.; Atwood, J. L.; Zhang, H. The Synthesis and Characterisation, Including X-Ray Diffraction Study, of [Th{η–$C_5H_3(SiMe_3)_2$}$_3$]; the First Thorium(III) Crystal Structure. *J. Chem. Soc., Chem. Commun.* **1986**, *453*, 1148–1149, DOI: 10.1039/C39860001148.

(9) Langeslay, R. R.; Fieser, M. E.; Ziller, J. W.; Furche, F.; Evans, W. J. Synthesis, Structure, and Reactivity of Crystalline Molecular Complexes of the {[$C_5H_3(SiMe_3)_2$]$_3$Th}$^{1-}$ Anion Containing Thorium in the Formal +2 Oxidation State. *Chem. Sci.* **2015**, *6*, 517–521, DOI: 10.1039/C4SC03033H.

(10) Peterson, J. K.; MacDonald, M. R.; Ziller, J. W.; Evans, W. J. Synthetic Aspects of $(C_5H_4SiMe_3)_3$Ln Rare-Earth Chemistry: Formation of $(C_5H_4SiMe_3)_3$Lu via [$(C_5H_4SiMe_3)_2$Ln]$^+$ Metallocene Precursors. *Organometallics* **2013**, *32*, 2625–2631, DOI: 10.1021/om400116d.

(11) Windorff, C. J.; MacDonald, M. R.; Ziller, J. W.; Evans, W. J. Trimethylsilylcyclopentadienyl (Cp′) Uranium Chemistry: Synthetic and Structural Studies of Cp′$_4$U and Cp′$_3$UX (X = Cl, I, Me). *Z. Anorg. Allg. Chem.* **2017**, *643*, 2011–2018, DOI: 10.1002/zaac.201700323.

(12) del Mar Conejo, M.; Parry, J. S.; Carmona, E.; Schultz, M.; Brennann, J. G.; Beshouri, S. M.; Andersen, R. A.; Rogers, R. D.; Coles, S.; Hursthouse, M. Carbon Monoxide and Isocyanide Complexes of Trivalent Uranium Metallocenes. *Chem. Eur. J.* **1999**, *5*, 3000–3009.

(13) Hitchcock, P. B.; Lappert, M. F.; Maron, L.; Protchenko, A. V. Lanthanum Does Form Stable Molecular Compounds in the +2 Oxidation State. *Angew. Chem. Int. Ed.* **2008**, *47*, 1488–1491, DOI: 10.1002/anie.200704887.

(14) Clappe, C.; Leveugle, D.; Hauchard, D.; Durand, G. Electrochemical Studies of Tricyclopentadienyl Uranium IV Chloride Complexes: $(RCp)_3UCl$ (RCp = RC_5H_4 with R = H; Me: CH_3; ^tBu: $(CH_3)_3C$; TMS: $(CH_3)_3Si$) Evidence of a Disproportionation Mechanism in Oxidation. *J. Electroanal. Chem.* **1998**, *448*, 95–103, DOI: 10.1016/S0022-0728(98)00029-1.

(15) Hauchard, D.; Cassir, M.; Chivot, J.; Ephritikhine, M. Electrochemical Study of Uranium(IV) and Uranium(IV) Organometallic Compounds in Tetrahydrofuran by Means of Conventional Microelectrodes and Ultramicroelectrodes. Part I. Application to the Na(Hg) Reduction of Cp3UCl (Cp = η–C_5H_5). *J. Electroanal. Chem.* **1991**, *313*, 227–241, DOI: 10.1016/0022-0728(91)85182-O.

(16) La Pierre, H. S.; Kameo, H.; Halter, D. P.; Heinemann, F. W.; Meyer, K. Coordination and Redox Isomerization in the Reduction of a Uranium(III) Monoarene Complex. *Angew. Chem. Int. Ed.* **2014**, *53*, 7154–7157, DOI: 10.1002/anie.201402048.

(17) Guo, F. S.; Tsoureas, N.; Huang, G. Z.; Tong, M. L.; Mansikkamäki, A.; Layfield, R. A. Isolation of a Perfectly Linear Uranium(II) Metallocene. *Angew. Chem. Int. Ed.* **2020**, *59*, 2299–2303, DOI: 10.1002/anie.201912663.

(18) Evans, W. J.; Davis, B. L. Chemistry of Tris (Pentamethylcyclopentadienyl) f-Element Complexes, $(C_5Me_5)_3M$. *Chem. Rev.* **2002**, *102*, 2119–2136, DOI: 10.1021/cr010298r.

(19) Zachmanoglou, C. E.; Docrat, A.; Bridgewater, B. M.; Parkin, G.; Brandow, C. G.; Bercaw, J. E.; Jardine, C. N.; Lyall, M.; Green, J. C.; Keister, J. B. The Electronic Influence of Ring Substituents and Ansa Bridges in Zirconocene Complexes as Probed by Infrared Spectroscopic, Electrochemical, and Computational Studies. *J . Am. Chem. Soc.* **2002**, *124*, 9525–9546, DOI: 10.1021/ja020236y.

(20) Kotyk, C. M.; MacDonald, M. R.; Ziller, J. W.; Evans, W. J. Reactivity of the Ln^{2+} Complexes [K(2.2.2-Cryptand)][$(C_5H_4SiMe_3)_3Ln$]: Reduction of Naphthalene and Biphenyl. *Organometallics* **2015**, *34*, 2287–2295, DOI: 10.1021/om501063h.

(21) Rinehart, J. D.; Fang, M.; Evans, W. J.; Long, J. R. A N_2^{3-}Radical-Bridged Terbium Complex Exhibiting Magnetic Hysteresis at 14 K. *J. Am. Chem. Soc.* **2011**, *133*, 14236–14239, DOI: 10.1021/ja206286h.

(22) Korobkov, I.; Arunachalampillai, A.; Gambarotta, S. Cyclometalation and Solvent Deoxygenation during Reduction of a Homoleptic $Th(OAr)_4$ Complex: Serendipitous Formation of a Terminally Bonded Th-OH Function. *Organometallics* **2004**, *23*, 6248–6252, DOI: 10.1021/om049369d.

(23) Pereira, L. C. J.; Camp, C.; Coutinho, J. T.; Chatelain, L.; Maldivi, P.; Almeida, M.; Mazzanti, M. Single-Molecule-Magnet Behavior in Mononuclear Homoleptic Tetrahedral Uranium(III) Complexes. *Inorg. Chem.* **2014**, *53*, 11809–11811, DOI: 10.1021/ic501520c.

(24) Müller, I.; Schneider, C.; Pietzonka, C.; Kraus, F.; Werncke, C. G. Reduction of 2,2’-Bipyridine by Quasi-Linear 3d-Metal(I) Silylamides-A Structural and Spectroscopic

Study. *Inorganics* **2019**, *7*, DOI: 10.3390/inorganics7100117.

(25) Arnold, P. L.; Wang, K.; Gray, S. J.; Moreau, L. M.; Booth, C. H.; Curcio, M.; Wells, J. A. L.; Slawin, A. M. Z. Dicerium Letterbox-Shaped Tetraphenolates: f-Block Complexes Designed for Two-Electron Chemistry. *Dalton Trans.* **2020**, *49*, 877–884, DOI: 10.1039/c9dt03291f.

(26) Werncke, C. G.; Müller, I. The Ambiguous Behaviour of Diphosphines towards the Quasilinear Iron(I) Complex $[Fe(N(SiMe_3)_2)_2]^-$ – between Inertness, P-C Bond Cleavage and C-C Double Bond Isomerisation. *Chem. Commun.* **2020**, *56*, 2268–2271, DOI: 10.1039/c9cc08968c.

(27) Balatoni, I.; Hlina, J.; Zitz, R.; Pöcheim, A.; Baumgartner, J.; Marschner, C. Disilene Fluoride Adducts versus *β*-Halooligosilanides. *Inorg. Chem.* **2019**, *58*, 14185–14192, DOI: 10.1021/acs.inorgchem.9b02223.

(28) Morris, D. E.; Da Re, R. E.; Jantunen, K. C.; Castro-Rodriguez, I.; Kiplinger, J. L. Trends in Electronic Structure and Redox Energetics for Early-Actinide Pentamethylcyclopentadienyl Complexes. *Organometallics* **2004**, *23*, 5142–5153, DOI: 10.1021/om049634v.

(29) Inman, C. J.; Cloke, F. G. N. The Experimental Determination of Th(IV)/Th(III) Redox Potentials in Organometallic Thorium Complexes. *Dalton Trans.* **2019**, *48*, 10782–10784, DOI: 10.1039/c9dt01553a.

(30) Kuehl, C. J.; Da Re, R. E.; Scott, B. L.; Morris, D. E.; John, K. D. Toward New Paradigms in Mixed-Valency: Ytterbocene-Terpyridine Charge-Transfer Complexes. *Chem. Commun.* **2003**, *3*, 2336–2337, DOI: 10.1039/b306484k.

(31) Da Re, R. E.; Kuehl, C. J.; Brown, M. G.; Rocha, R. C.; Bauer, E. D.; John, K. D.; Morris,

D. E.; Shreve, A. P.; Sarrao, J. L. Electrochemical and Spectroscopic Characterization of the Novel Charge-Transfer Ground State in Diimine Complexes of Ytterbocene. *Inorg. Chem.* **2003**, *42*, 5551–5559, DOI: 10.1021/ic030069i.

(32) APEX2 Version 2014.11-0, Bruker AXS, Inc.; Madison, WI 2014.

(33) SAINT Version 8.34a, Bruker AXS, Inc.; Madison, WI 2013.

(34) Sheldrick, G. M. SADABS, Version 2014/5, Bruker AXS, Inc.; Madison, WI 2014.

(35) Sheldrick, G. M. SHELXTL, Version 2014/7, Bruker AXS, Inc.; Madison, WI 2014.

(36) International Tables for Crystallography 1992, Vol. C., Dordrecht: Kluwer Academic Publishers.

Definitions:

$wR2 = [\Sigma[w(F_o^2-F_c^2)^2] / \Sigma[w(F_o^2)^2]]^{1/2}$

$R1 = \Sigma||F_o|-|F_c|| / \Sigma|F_o|$

Goof = S = $[\Sigma[w(F_o^2-F_c^2)^2] / (n-p)]^{1/2}$ where n is the number of reflections and p is the total number of parameters refined.
